# Synthesis of Non-Steroidal Anti-Inflammatory Drugs Pelubiprofen, Loxoprofen, and Carprofen Through Batch and Continuous-Flow Photo-Favorskii Rearrangement

**DOI:** 10.3390/molecules31162910

**Published:** 2026-08-20

**Authors:** Sara Ferrario, Paolo Celestini, Gabriele Rebuzzini, Sergio Rossi, Maurizio Benaglia

**Affiliations:** 1 Dipartimento di Chimica, Università degli Studi di Milano, Via C. Golgi 19, 20133 Milan, MI, Italy; sara.ferrario@unimi.it (S.F.); sergio.rossi@unimi.it (S.R.); 2 Cosma S.p.A., Via B. Colleoni 15–17, 24040 Ciserano, BG, Italy; p.celestini@cosma.it (P.C.); g.rebuzzini@cosma.it (G.R.)

**Keywords:** photochemistry, aryl propionic acids, photo-Favorskii rearrangement, flow chemistry, non-steroidal anti-inflammatory drugs

## Abstract

Novel and efficient total syntheses of the nonsteroidal anti-inflammatory drugs Pelubiprofen and Loxoprofen via a photo-Favorskii rearrangement are reported herein. The key photochemical transformation was optimized under both batch and continuous-flow conditions using a suitably functionalized chloro-phenylpropan-1-one derivative, affording the target 2-arylpropionic acid in excellent yield. Implementation under continuous-flow conditions increased process productivity and enabled gram-scale operation. Aerobic oxidation of the benzylic position to the corresponding aldehyde, followed by Claisen–Schmidt condensation with cyclohexanone, afforded Pelubiprofen in 35% overall yield. Alternatively, condensation with cyclopentanone afforded the corresponding α,β-unsaturated enone intermediate, whose selective reduction under flow conditions enabled access to Loxoprofen in 28% overall yield. The versatility of the methodology was further demonstrated through the synthesis of Carprofen, highlighting the broader applicability of the photo-Favorskii rearrangement to the synthesis of APIs through previously unreported synthetic routes.

## 1. Introduction

**Scheme 1:**
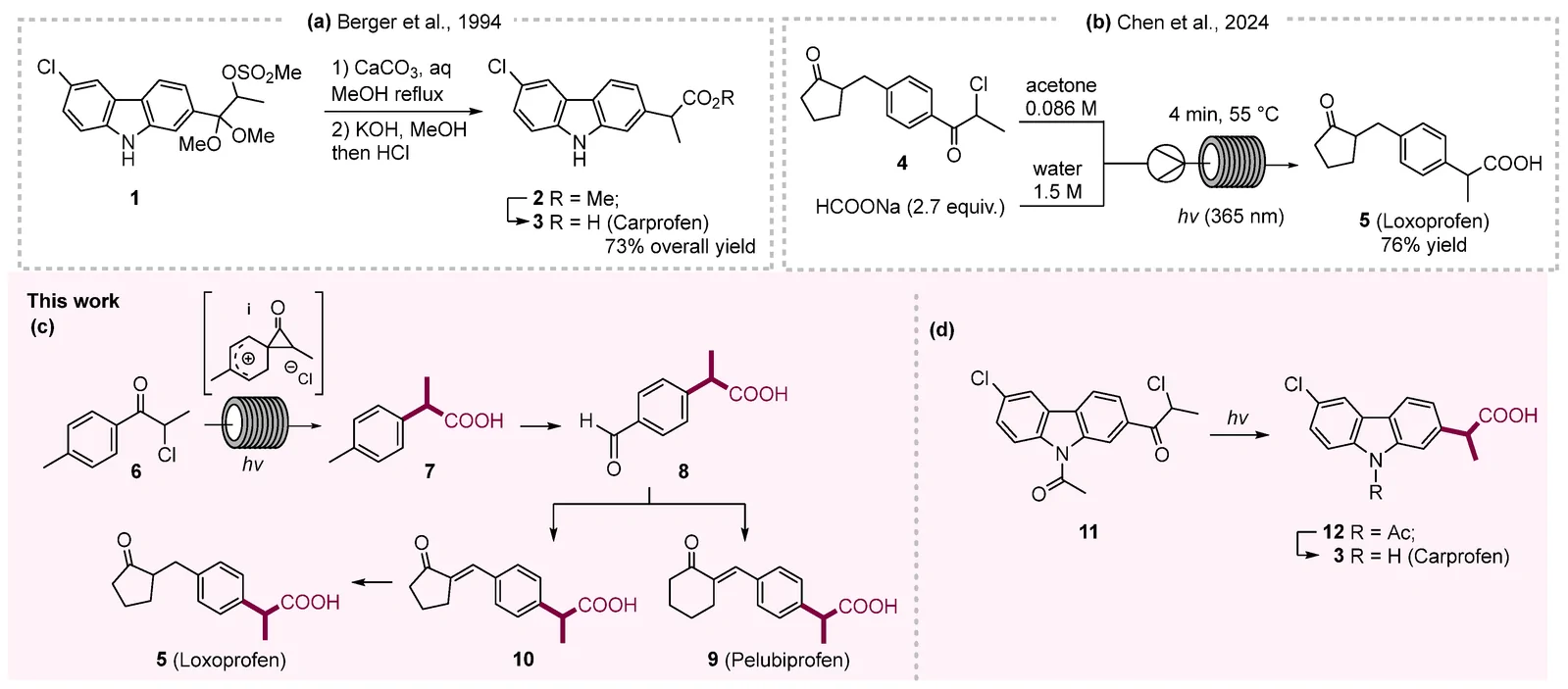
(**a**) Synthesis of Carprofen via Favorskii rearrangement under batch conditions reported by Berger [2]; (**b**) synthesis of Loxoprofen via photo-Favorskii reaction in flow reported by Chen [16]; (**c**) novel total synthesis of Loxoprofen and Pelubiprofen via photo-Favorskii rearrangement under flow conditions; (**d**) first synthesis of Carprofen via photo-Favorskii rearrangement.

Non-steroidal anti-inflammatory drugs (NSAIDs) are extensively employed for the treatment of pain and inflammation, particularly in rheumatoid arthritis [1]. Among NSAIDs, the most common class is represented by 2-arylpropionic acids [2], comprising pharmaceutically active molecules such as Ibuprofen, Naproxen, Ketoprofen, Carprofen, Loxoprofen and Pelubiprofen. Over the past decades, the base-induced Favorskii rearrangement, first reported by Aleksej Evgrafovič Favorskij in 1894 [3,4], has found valuable applications in organic synthesis [5], including the preparation of NSAIDs. A notable example was reported by Berger in 1994 and involved the conversion of mesylate intermediate **1** into methyl carprofen ester **2**, which was subsequently transformed into Carprofen **3** through a modified Favorskii rearrangement (Scheme 1a) [2].

After Favorskii’s original discovery, the photochemical rearrangement of α-haloketones was investigated in further studies [6,7,8]. In this context, Ayyangar and co-workers reported stereoselective synthesis of Ibuprofen and Ketoprofen, extending the reaction scope to different para-substituted α-chloropropiophenones bearing alkyl, halogen, or alkoxy groups and affording the corresponding α-arylpropionic acids in moderate to excellent yields upon UV light irradiation [9]. Notably, although stereoselective approaches have been developed, metabolic inversion of the (*R*)-enantiomer to the corresponding (*S*)-enantiomer has been reported in vivo for several 2-arylpropionic acids [10,11,12,13].

However, the first synthesis of Ibuprofen via a photo-Favorskii rearrangement under continuous-flow conditions was reported only in 2016 by Baxendale et al. [14], whereas in 2023, Yamaguchi developed a telescoped synthetic approach for the same anti-inflammatory drug [15]. One year later, Chen demonstrated the broad applicability of the photo-Favorskii reaction in continuo, including its successful extension to the pharmaceutically relevant compound Loxoprofen, starting from the corresponding α-chloro derivative **4** to obtain the final drug **5** in high yield (Scheme 1b) [16].

Based on these precedents and on the demonstrated suitability of photochemistry for continuous-flow processing and scale-up [17,18,19,20,21,22,23,24,25], an alternative strategy for Loxoprofen **5** and Pelubiprofen **9** was developed herein. Unlike the direct photo-Favorskii synthesis of Loxoprofen reported by Chen et al., which relies on a drug-specific advanced α-chloro precursor, the present strategy starts from toluene and introduces the rearrangement at an earlier stage of the synthesis.

According to the synthetic procedure reported in Scheme 1c, toluene was first converted into 2-chloro-1-(4-methylphenyl)propan-1-one **6**, whose photo-Favorskii rearrangement afforded 2-(p-tolyl)propanoic acid **7**. Subsequent benzylic oxidation provided aldehyde **8** as a common branching intermediate for the synthesis of either Pelubiprofen **9** or Loxoprofen **5**, thus enabling access to two different NSAIDs through a single shared synthetic sequence. The scope of the transformation was further extended to the synthesis of Carprofen **3** by the conversion of *N*-acetyl-protected α-chloropropiophenone derivative **11** into the corresponding rearranged propionic acid intermediate **12** (Scheme 1d).

## 2. Results and Discussion

### 2.1. Pelubiprofen

As an initial approach, the strategy reported by Chen and co-workers for the synthesis of Loxoprofen was applied to Pelubiprofen [16]. Accordingly, the corresponding α-chloro and acetoxy derivatives of Pelubiprofen were prepared and subjected to photo-Favorskii rearrangement. However, both substrates proved to be unreactive under a range of irradiation sources and solvent systems, leading to complete recovery of the unreacted starting materials. These results suggest suppression of the rearrangement by the *para*-alkenyl substituent (see Supporting Information for further details). Therefore, an alternative strategy was developed, enabling the efficient synthesis of both anti-inflammatory drugs **5** and **9** through a common intermediate in four synthetic steps for Pelubiprofen and five steps for Loxoprofen, combining batch and continuous-flow protocols.

According to a reported procedure, Friedel–Crafts acylation of toluene afforded 2-chloro-1-(4-methylphenyl)propan-1-one **6** in near-quantitative yield (99%, see Supporting Information), and the crude product was used directly in the subsequent step without further purification [26]. Then, with inspiration taken from literature precedents [9,14,16], its photo-Favorskii rearrangement to arylpropionic acid **7** was investigated under UVA irradiation. In analogy with the pathway previously proposed for Ibuprofen [14], the transformation is suggested to proceed through spirocyclopropenone intermediate **i**, followed by rearomatization to give 2-(*p*-tolyl)propanoic acid **7** (Scheme 1).

The reaction was initially carried out in an acetone/water mixture (9:1) under irradiation with a 370 nm Kessil lamp. In the absence of external cooling, the reaction temperature reached 45 °C. Reaction time screening (entries 1–3, Table 1) showed that 1 h of irradiation led to the formation of appreciable amounts of 4-methylbenzoic acid **14** which proved particularly difficult to separate from product **7** because of the similar polarity and acidity of the two compounds. Extending the irradiation time to 1.5 h provided optimal conditions and enabled the isolation of **7** in 50% yield. In addition to the desired product, reduced byproduct **13**, commonly referred to as a Norrish type-I product [8,14,27], was consistently detected. Varying the temperature from 45 to 25 °C, by employing a dual-fan cooling system, had no significant impact on the reaction outcome (entry 4). In contrast, reducing the lamp intensity by 50% resulted in a lower yield (entry 5), highlighting the importance of high irradiation intensity (399 mW/cm^2^, 100% output, 45 W power consumption) for efficient conversion. As the solvent system, acetone/water (9:1) provided better results compared to acetonitrile/water mixtures (entry 6). A concentration of 0.1 M was selected to enhance productivity in view of the subsequent development of the transformation under continuous-flow conditions (entries 7–8).

Additionally, the effect of water content was examined by varying the acetone/water ratio, with the 9:1 mixture proving to be the optimal solvent composition (see Table S4 in the Supporting Information). Further optimization showed that the addition of propylene oxide (2.9 equiv.) as an acid scavenger for the hydrochloric acid generated during the reaction significantly improved the outcome, affording product **7** in 83% isolated yield after 1.5 h of irradiation at 45 °C (entry 9). Its addition also reduced the formation of byproduct **13** from 20% to 4%, consistent with suppression of a competing photochemical pathway [9,14,28]. Control experiments in the absence of light confirmed that UV irradiation is essential for the rearrangement (entry 10).

**Scheme 2:**
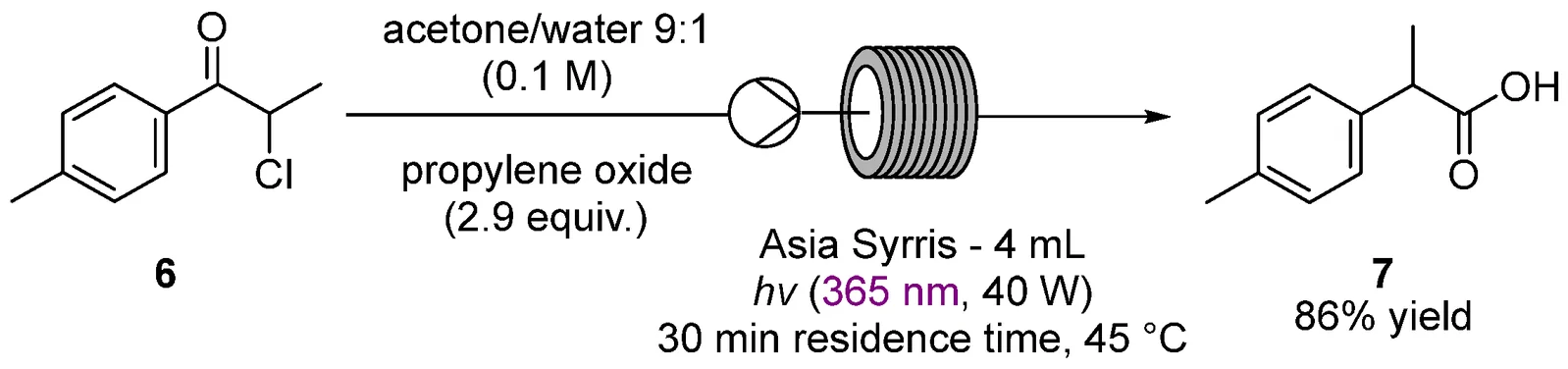
Optimized reaction conditions for the photo-Favorskii rearrangement of 2-chloro-1-(4-methylphenyl)propan-1-one **6** to 2-(*p*-tolyl)propanoic acid **7** under continuous-flow conditions.

At this stage, the photo-Favorskii rearrangement of **6** was investigated under continuous-flow conditions using an Asia Premium Flow Chemistry System (Syrris) equipped with 365 nm LEDs (40 W). Optimization of the reaction under flow conditions identified a 30 min residence time at 45 °C in acetone/water (9:1) at 0.1 M concentration as the optimal parameters, affording 2-(*p*-tolyl)propanoic acid **7** in 86% isolated yield with only trace amounts of byproduct **13**. Notably, no formation of 4-methylbenzoic acid **14** was detected under these conditions (Scheme 2).

**Scheme 3:**
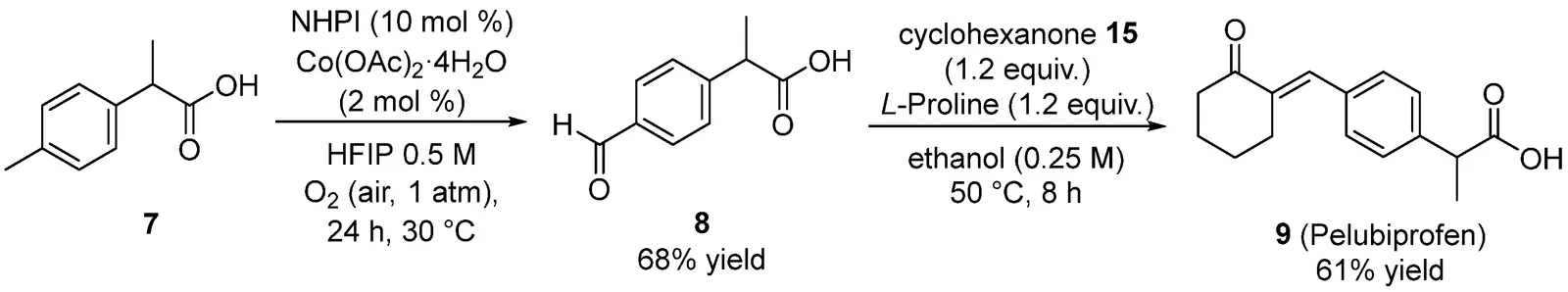
Synthesis of Pelubiprofen **9**.

To prevent overheating during prolonged irradiation experiments (up to 20 h) in which the reactor temperature could rise from 45 to as high as 60 °C, an air-cooling system was used to maintain the temperature at 25 °C and ensure reproducible reaction conditions. With this experimental set-up, product **7** was obtained in 62% yield with a residence time of only 5 min, corresponding to a productivity of 490 mg · h^−1^ and to a 14-fold enhancement relative to batch conditions (see Tables S8 and S9 for further details). A gram-scale experiment (25 mmol) was then conducted using a residence time of 15 min, with the reactor operated continuously for a total processing time of 15.6 h without any operational issues. After a simple acid–base extraction, 2-(*p*-tolyl)propanoic acid **7** was isolated in 58% yield (2.38 g), corresponding to a productivity of 153 mg · h^−1^ with no chromatographic purification required. Finally, aerobic autoxidation of compound **7** to the corresponding unreported benzylic aldehyde **8** was investigated at 30 °C, employing *N*-hydroxyphthalimide (NHPI) as a phthalimido-*N*-oxyl (PINO) radical precursor and cobalt(II) acetate tetrahydrate as a redox metal catalyst in hexafluoroisopropanol (HFIP)—a fluorinated, mildly acidic, and highly polar solvent whose strong hydrogen-bond-donor ability promotes interactions with carbonyl-containing species [29,30]. The process typically exhibits improved efficiency and selectivity, favoring aldehyde formation over further oxidation to the corresponding acid and affording the desired aldehyde **8** in 68% yield (Scheme 3). As the final step in the synthesis of Pelubiprofen, a modified literature procedure was applied to the *L*-proline-catalyzed Claisen–Schmidt condensation of aldehyde **8** with cyclohexanone **15** in ethanol at 50 °C [31]. After 8 h, Pelubiprofen **9** was isolated in 61% yield.

Considering the complete four-step sequence, the newly developed route afforded Pelubiprofen in 34% overall yield when the photo-Favorskii rearrangement was performed under batch conditions and in 35% overall yield when the photochemical step was conducted under continuous-flow conditions.

### 2.2. Loxoprofen

**Scheme 4:**
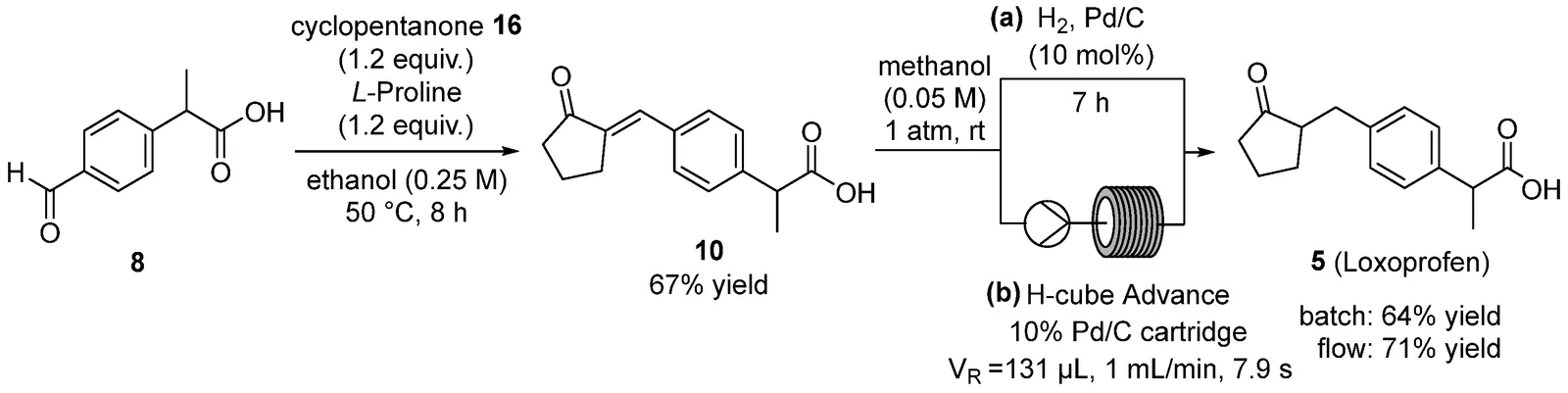
Synthesis of Loxoprofen **5**; (**a**) batch hydrogenation with H_2_/Pd/C and (**b**) continuous-flow hydrogenation using an H-Cube Advance reactor equipped with a 10% Pd/C cartridge.

The synthesis of Loxoprofen was completed starting from aldehyde **8**, which was obtained via benzylic oxidation as described above of the photo-Favorskii rearrangement product **7**. The *L*-proline-catalyzed Claisen–Schmidt condensation of aldehyde **8** with cyclopentanone **16** in ethanol at 50 °C afforded intermediate **10** in 67% yield after 8 h. Catalytic hydrogenation of **10** with 10 mol% Pd/C in methanol at room temperature under 1 atm of H_2_ afforded Loxoprofen **5** in 64% yield under batch conditions (pathway a, Scheme 4). As an alternative, the hydrogenation was performed under continuous-flow conditions using a ThalesNano H-Cube Advance reactor operated in full-hydrogen mode and equipped with a reusable pre-packed 10% Pd/C catalyst cartridge. Notably, Loxoprofen **5** was obtained in 71% yield with a residence time of 7.9 s only at a flow rate of 1 mL · min^−1^ (pathway b, Scheme 4). The flow process achieved a productivity of 525 mg · h^−1^ and a space-time yield of 4008 g · h^−1^ · L^−1^, corresponding to 48- and 3644-fold increases compared to the batch protocol, respectively (see Table S10 in the Supporting Information).

Considering the newly developed complete five-step sequence, Loxoprofen was synthesized in 24% overall yield when both the photo-Favorskii rearrangement and the hydrogenation were performed under batch conditions. Implementation of both transformations under continuous-flow conditions increased the overall yield to 28%.

### 2.3. Carprofen

Encouraged by the successful syntheses of Loxoprofen **5** and Pelubiprofen **9**, the photo-Favorskii rearrangement was further applied to the preparation of Carprofen **3**. Although the synthesis of Carprofen through a conventional Favorskii rearrangement has previously been reported [2], its photoinduced counterpart remains unexplored. Accordingly, known Carprofen intermediate **11** was initially irradiated with a 370 nm Kessil lamp in an acetone/water 9:1 mixture for 5 h at 45 °C. In the absence of propylene oxide, no formation of product **12** was observed (Table 2, entry 1), whereas its addition as an HCl scavenger afforded only trace amounts of the desired product (entry 2). This poor reactivity was attributed, at least in part, to the limited solubility of substrate **11** in acetone/water. DMF/water and DMA/water mixtures (91:9) were therefore evaluated to ensure complete substrate dissolution while retaining the high water content required for the photo-Favorskii rearrangement [32]. In the presence of propylene oxide, the same transformation performed in DMF/water reaction medium afforded product **12** in 16% yield (entry 3), whereas the use of DMA/water increased the yield to 23%. Sodium formate and epichlorohydrin were subsequently evaluated as alternative HCl scavengers, providing arylpropionic acid **12** in 12% and 17% yield, respectively (entries 5 and 6).

Prolonged irradiation times did not improve the reaction outcome, with 16 h irradiation affording **12** in 24% yield (entry 7), while shortening the reaction time to 2.5 h resulted in a significant decrease in yield (entry 8). Lamp screening further revealed inferior performance when a 254 nm UVC lamp was used (entry 9). Since degradation products were consistently observed under all irradiation conditions, and considering that photochemical decomposition pathways are known to compete with intermolecular rearrangements under UV irradiation [33], a 390 nm Kessil lamp was selected. Under these conditions, rearrangement product **12** was obtained in comparable yield (entry 10) after 5 h of irradiation at 40 °C, with no further improvement upon extension of the irradiation time to 16 h (entry 11). Consistently, a control experiment confirmed that light irradiation was essential for the radical transformation (entry 12).

Overall, the moderate yields observed under all conditions may arise from the less favorable rearomatization step of the carbazole moiety required to complete the photo-Favorskii rearrangement. Despite optimization of the solvent composition, irradiation wavelength, and reaction time, the transformation is characterized by moderate conversion and competing photodegradation; these probably reflect the different electronic properties of the heteroaromatic carbazole system, which may lead to a less favorable rearomatization step than that in a simple aromatic ring. This outcome is further consistent with the relatively low efficiency observed under flow conditions, where the reaction was performed under 365 nm irradiation in DMA/water (91:9) at 0.1 M concentration, with 11% yield after a 30 min residence time. Although the chemical yield was lower than that in batch, the reduction of the reaction time from 16 h to 30 min, together with the fivefold higher productivity, provides a complementary measure of the performance of the flow process (see Supporting Information).

**Scheme 5:**
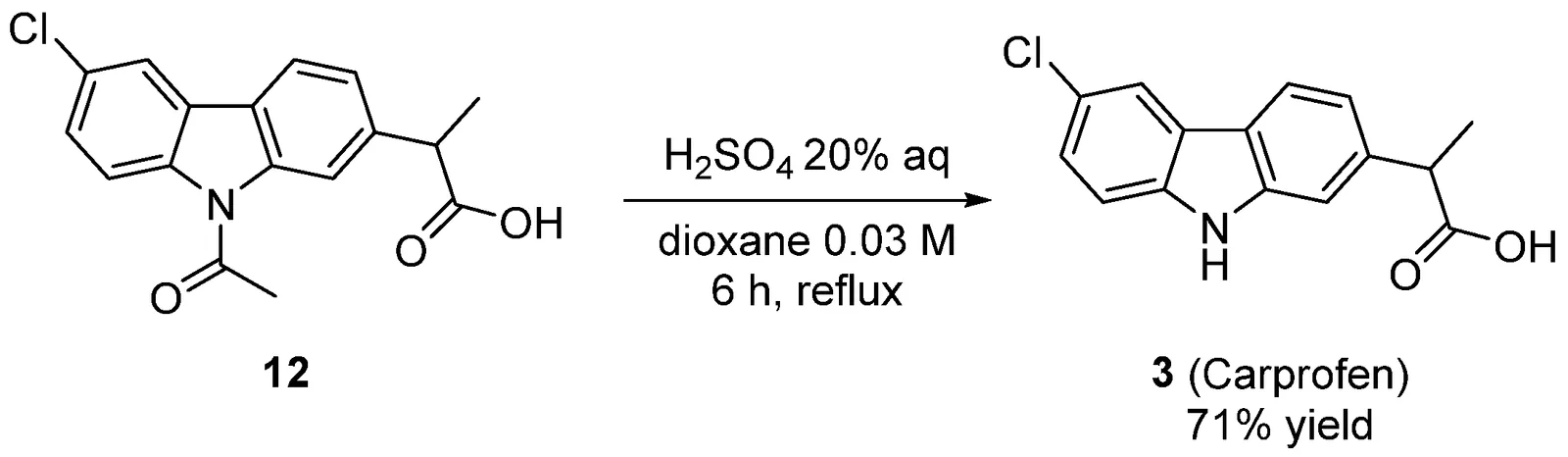
Synthesis of Carprofen **3**.

As the final step, carbazole intermediate **12** was *N*-deprotected under conditions adapted from a reported procedure [34]. Treatment with 20% aqueous sulfuric acid in dioxane (0.03 M) under reflux for 6 h afforded Carprofen **3** in 71% isolated yield (Scheme 5). The complete two-step sequence provided Carprofen in 17% overall yield under batch conditions.

## 3. Materials and Methods

Reagents were purchased at the highest commercial quality and used as received. Carbazole intermediate **11** was kindly provided by Cosma S.p.A. (Ciserano, Italy) and used without further purification. Anhydrous solvents were purchased in AcroSeal^®^ bottles and used as received. Reactions were monitored by thin-layer chromatography (TLC) on Macherey-Nagel pre-coated silica gel plates (0.25 mm) and visualized by UV irradiation at 254 nm. For aryl propionic acid detection, 2 drops of formic acid were added to each 10 mL of eluent mixture. Flash chromatography was performed via standard flash column chromatography on Merck silica gel 60 (particle size: 0.04–0.063 mm). Petroleum ether, hexane, ethyl acetate, dichloromethane and methanol were used as standard eluent solvents.

^1^H-NMR spectra were recorded on spectrometers operating at 300 MHz (Bruker Avance 300, Bruker BioSpin GmbH & Co. KG, Ettlingen, Germany) or at 400 MHz (Bruker NEO400 Avance, Bruker BioSpin GmbH & Co. KG). The chemical shifts are reported in ppm (δ), with the solvent reference relative to tetramethyl silane (TMS) employed as the internal standard (CDCl_3_ δ = 7.26 ppm, MeOD δ = 3.31 ppm, DMSO δ = 2.50 ppm). ^1^H NMR data are reported as follows: chemical shift (ppm), multiplicity (s = singlet, br. s. = broad singlet, d = doublet, t = triplet, q = quartet, quint = quintet, sext = sextet, hept = heptet, dd = doublet of doublets, ddd = doublet of doublets of doublets, td = triplet of doublets, qd = quartet of doublets, m = multiplet), coupling constants (Hz), and numbers of protons. ^13^C-NMR spectra were recorded at 25 °C on a 300 MHz or 400 MHz spectrometer (Bruker Avance 300, Bruker NEO400 Avance) operating at 75 or 101 MHz, with complete proton decoupling. Carbon chemical shifts are reported in ppm (δ) relative to TMS with the respective solvent resonance as the internal standard (CDCl_3_ δ = 77.16 ppm, MeOD δ = 49.00 ppm, DMSO δ = 39.52 ppm). Structural assignments were made with additional information from gCOSY, gHSQC, and gHMBC experiments. Deuterated solvents acquired from Merck (Darmstadt, Germany) were used as supplied.

High-resolution mass spectra (HRMS) were obtained from the Unitech COSPECT center, University of Milan, on a Synapt G2-Si HDMS (Waters Corporation, Milford, MA, USA) using an Acquity UPLC I-Class photodiode array (PDA) detector (Waters Corporation). The samples were ionized in positive ion mode using ESI, ESCI or APCI ionization sources.

UV-Vis spectra were recorded using a Nicolet Evolution 500 (Thermo Fisher Scientific, Waltham, MA, USA) and quartz cuvettes with path length of 1 mm in ACN at the appropriate concentrations, using a bandwidth of 1 nm and a scan rate of 120 nm min^−1^.

Batch reactions were performed using either quartz vials (for 254 nm light irradiation) or glass vials (for 370 and 390 nm light irradiation).

Continuous-flow photochemical reactions were performed using an Asia Premium Flow Chemistry System (Syrris Ltd., Royston, UK) equipped with high-power LEDs (365 nm, 41.8 W) and fluoropolymer tubing as the reactor coil of 4 mL volume.

Continuous-flow hydrogenation reactions were performed using a ThalesNano H-Cube Advance reactor (ThalesNano Inc., Budapest, Hungary), distributed by StepBio S.r.l. (Bologna, Italy) equipped with an integrated in situ hydrogen generator based on water electrolysis and a pre-packed 10% Pd/C catalyst cartridge (CatCart^®^ technology, Edinburgh, UK) with a reactor volume of 0.131 mL.

### 3.1. General Procedure for the Synthesis of 2-(p-Tolyl)propanoic Acid (***7***) via a Photo-Favorskii Rearrangement in Batch (GP1)

A 10 mL vial was charged with 2-chloro-1-(*p*-tolyl)propan-1-one **6** (73 mg, 0.4 mmol, 0.1 M) and the scavenger (typically propylene oxide, 80 µL, 1.14 mmol, 2.9 equiv.) dissolved in the desired solvent (typically a 9:1 solvent mixture of acetone/water, 4 mL). The yellowish reaction mixture was irradiated using a 370 nm Kessil lamp for the desired time at the desired temperature (typically 1.5 h, 45 °C). After that, the reaction mixture was concentrated under reduced pressure. A 5 µL volume of dibromomethane as the internal standard was added to the resulting crude for the ^1^H-NMR yield. 2-(*p*-tolyl)propanoic acid **7** was obtained by column chromatography on silica gel (hexane/ethyl acetate from 9:1 to 7:3 + 0.25 vol% formic acid) in excellent yields (up to 83%) as a yellowish solid. For larger-scale experiments, chromatographic purification was replaced by acid–base extraction. The crude mixture was dissolved in EtOAc and extracted with 0.125 M aqueous NaHCO_3_. The combined aqueous layers were acidified with 4 M HCl and extracted with EtOAc. The combined organic layers were dried over anhydrous Na_2_SO_4_, filtered, and concentrated under reduced pressure to afford the desired product.

### 3.2. General Procedure for the Synthesis of 2-(p-Tolyl)propanoic Acid (***7***) via a Photo-Favorskii Rearrangement in Flow (GP2)

A vial was charged with 2-chloro-1-(*p*-tolyl)propan-1-one **6** (73 mg, 0.4 mmol, 0.1 M) and propylene oxide (80 µL, 1.14 mmol, 2.9 equiv.) dissolved in a 9:1 mixture of acetone/water (4 mL). The yellowish reaction mixture was fed inside the coil reactor (0.5 mm ID, total volume of 4 mL) at the desired flow rate (typically 133 µL· min^−1^, corresponding to a 30 min residence time) and irradiated with 365 nm LEDs using the Asia System (Syrris) at the desired temperature. After the irradiation, the reaction mixture was concentrated under reduced pressure. A 5 µL volume of dibromomethane as the internal standard was added to the resulting crude to calculate the ^1^H-NMR yield. 2-(*p*-tolyl)propanoic acid **7** was obtained by column chromatography on silica gel (hexane/ethyl acetate from 9:1 to 7:3, adding 0.25 vol% formic acid) in good yields (up to 86%) as a yellowish solid (up to 56.5 mg). For larger-scale experiments, chromatographic purification was replaced by acid–base extraction. The crude mixture was dissolved in EtOAc and extracted with 0.125 M aqueous NaHCO_3_. The combined aqueous layers were acidified with 4 M HCl and extracted with EtOAc. The combined organic layers were dried over anhydrous Na_2_SO_4_, filtered, and concentrated under reduced pressure to afford the desired product.

### 3.3. General Procedure for the Synthesis of 2-(9-Acetyl-6-chloro-9H-carbazol-2-yl)propanoic Acid (***12***) via a Photo-Favorskii Rearrangement in Batch (GP3)

A 10 mL glass vial was charged with 1-(9-acetyl-6-chloro-9H-carbazol-2-yl)-2-chloropropan-1-one **11** (133.7 mg, 0.4 mmol, 0.1 M) and the desired scavenger (typically, propylene oxide, 80 µL, 1.14 mmol, 2.9 equiv.) dissolved in the desired solvent (typically a 91:9 solvent mixture of DMA/water). The resulting reaction mixture was irradiated using the desired light source for the desired time at the desired temperature (typically, a 370 nm Kessil lamp was used at 45 °C for 5 h). The reaction mixture was then concentrated under reduced pressure, and the resulting residue was chromatographed on silica gel (hexane/ethyl acetate from 75:25 to 72:28, adding 0.25 vol% of formic acid) to obtain compound **12** in 23% yield as a yellowish solid (29 mg).

### 3.4. General Procedure for the Synthesis of 2-(9-Acetyl-6-chloro-9H-carbazol-2-yl)propanoic Acid (***12***) via a Photo-Favorskii Rearrangement in Flow (GP4)

A vial was charged with 1-(9-acetyl-6-chloro-9H-carbazol-2-yl)-2-chloropropan-1-one **11** (133.7 mg, 0.4 mmol, 0.1 M) and propylene oxide (80 µL, 1.14 mmol, 2.9 equiv.) dissolved in a solvent mixture of DMA/water (91:9). The resulting reaction mixture was irradiated with 365 nm LEDs using the Asia Premium Flow Chemistry System (Syrris) at 45 °C. The parameters were the following: 0.5 mm for the internal diameter, a reactor volume of 4 mL, 30 min as the residence time and 133 µL·min^−1^ was the set flow rate. After the irradiation, the reaction mixture was concentrated under reduced pressure, and the resulting residue was chromatographed on silica gel (hexane/ethyl acetate from 75:25 to 72:28, adding 0.25 vol% of formic acid) to obtain compound **12** in 11% yield as a yellowish solid (14 mg).

## 4. Conclusions

In conclusion, new routes for the synthesis of the non-steroidal anti-inflammatory drugs (NSAIDs) Pelubiprofen, Loxoprofen, and Carprofen were successfully developed through photo-Favorskii rearrangement, and the methodology was extended for the first time to the Carprofen scaffold. Pelubiprofen and Loxoprofen were accessed through the common key intermediate 2-(*p*-tolyl)propanoic acid, through a photo-Favorskii rearrangement, in 83% and 86% yield under batch and continuous-flow conditions, respectively. The target arylpropionic acid was obtained with a productivity of 490 mg h^−1^ and a residence time of only 5 min, while straightforward product isolation was achieved through a simple work-up. Implementation of the process on a commercially available Asia Syrris flow platform enabled scale-up to 25 mmol, providing more than 2 g of product and demonstrating the synthetic practicality and potential industrial applicability of the methodology. Subsequent mild aerobic oxidation, followed by *L*-proline-catalyzed Claisen–Schmidt condensation with cyclohexanone, provided Pelubiprofen in up to 35% overall yield. Condensation with cyclopentanone instead gave the corresponding α,β-unsaturated enone, which was selectively reduced under both batch and continuous-flow conditions to furnish Loxoprofen in up to 28% overall yield. Notably, the flow process, with a residence time of only 7.9 s, achieved a productivity of 525 mg · h^−1^ and a space-time yield of 4008 g · h^−1^ · L^−1^. Finally, the synthetic approach was extended to Carprofen, although in lower yield, marking the first reported application of a photo-Favorskii rearrangement to this scaffold.

These results demonstrate that the photo-Favorskii rearrangement is a versatile and scalable strategy for NSAID synthesis that combines photochemical activation with continuous-flow processing to enable practical access to a broad range of pharmaceutical targets.

## Figures and Tables

**Table 1:** Photo-Favorskii rearrangement optimization of 2-chloro-1-(4-methylphenyl)propan-1-one **6** under UVA irradiation in batch.

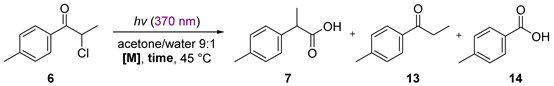					
Entry	Time (h)	Concentration [M]	7, ^1^H-NMR Yield (%) ^1^	13, ^1^H-NMR Yield (%) ^1^	14, ^1^H-NMR Yield (%) ^1^
1	4	0.1	52	20	-
2 ^2^	1.5	0.1	51 (50)	20	-
3	1	0.1	52	21	5
4 ^3^	1.5	0.1	50	22	-
5 ^4^	1.5	0.1	42	18	31
6 ^5^	1.5	0.1	38	15	28
7	1.5	0.2	41	33	5
8	1.5	0.05	59	15	-
9 ^2,6^	1.5	0.1	84 (83)	4	8
10 ^6,7^	4	0.1	-	-	-

^1^ Yields determined by ^1^H-NMR using dibromomethane as IS; ^2^ isolated yields reported in brackets; ^3^ reaction performed at 25 °C; ^4^ lamp operated at 50% intensity; ^5^ reaction performed in acetonitrile/water 9:1; ^6^ propylene oxide (2.9 equiv.) added as scavenger; ^7^ reaction performed in dark.

**Table 2:** Photo-Favorskii rearrangement of **11** to aryl propionic acid **12**.

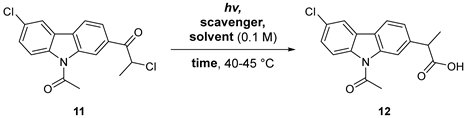					
Entry	hν (nm)	Solvent	Scavenger	Time (h)	Yield (%)
1	370	acetone/H_2_O 9:1		5	-
2	370	acetone/H_2_O 9:1	propylene oxide (2.9 equiv.)	5	traces
3	370	DMF/H_2_O 91:9	propylene oxide (2.9 equiv.)	5	16
4	370	DMA/H_2_O 91:9	propylene oxide (2.9 equiv.)	5	23
5	370	DMA/H_2_O 91:9	HCOONa (1.6 equiv.)	5	12
6	370	DMA/H_2_O 91:9	epichlorohydrin (2.5 equiv.)	5	17
7	370	DMA/H_2_O 91:9	propylene oxide (2.9 equiv.)	16	24
8	370	DMA/H_2_O 91:9	propylene oxide (2.9 equiv.)	2.5	10
9	254	DMA/H_2_O 91:9	propylene oxide (2.9 equiv.)	5	9
10	390	DMA/H_2_O 91:9	propylene oxide (2.9 equiv.)	5	22
11	390	DMA/H_2_O 91:9	propylene oxide (2.9 equiv.)	16	18
12	-	DMA/H_2_O 91:9	propylene oxide (2.9 equiv.)	16	-

## Data Availability

Data are contained within the article and Supplementary Materials.

## Supplementary Materials

The following supporting information can be downloaded at https://www.mdpi.com/article/10.3390/molecules31162910/s1, containing: synthetic procedures for starting materials, experimental details for photochemical reactions, characterization data for products, and NMR spectra for all described compounds. Refs. [35,36,37,38,39,40] are cited in the Supplementary Materials.

## Abbreviations

The following abbreviations are used in this manuscript:

API	Active pharmaceutical ingredient
IS	Internal standard
DMA	*N,N*-Dimethylacetamide
DMF	*N,N*-Dimethylformamide
HFIP	Hexafluoroisopropanol
DCE	1,2-Dichloroethane
LED	Light-emitting diode
NHPI	*N*-Hydroxyphthalimide
NMR	Nuclear magnetic resonance
NSAID	Non-steroidal anti-inflammatory drug
PINO	Phthalimide-*N*-oxyl radical
UVA	Ultraviolet A
UVC	Ultraviolet C
