# Supplementary material for: Synthesis of Non-Steroidal Anti-Inflammatory Drugs Pelubiprofen, Loxoprofen, and Carprofen Through Batch and Continuous-Flow Photo-Favorskii Rearrangement
Source: Molecules. 2026 Aug 20;31(16):2910. doi: 10.3390/molecules31162910 (PMC13515219; doi:10.3390/molecules31162910)
Supplement: Supplementary file 1 [file molecules-31-02910-s001.zip › molecules-4491007-supplementary.pdf]

# Supporting Information

# Synthesis of Non-Steroidal Anti-Inflammatory Drugs Pelubiprofen, Loxoprofen, and Carprofen through Batch and Continuous-Flow Photo-Favorskii Rearrangement

by

Sara Ferrario,[1] Paolo Celestini,[2] Gabriele Rebuzzini,[2] Sergio Rossi[1] and Maurizio Benaglia*[1]

[1] Dipartimento di Chimica, Università degli Studi di Milano, Via Camillo Golgi 19, 20133 Milano, Italy.

[2] Cosma S.p.A., Via B. Colleoni 15-17, 24040 Ciserano (BG), Italy

# Table of Contents


1. **General information**……………………………………………………… S3
2. **Description of the reactors**……………………………………………… S4
   2.1. UVC Sankyo Denki 254 nm lamp…………………………………………… S4
   2.2. Kessil lamp set-up……………………………………………………… S4
   2.3. Asia Premium Flow Chemistry System (Syrris)………………………… S5
   2.4. ThalesNano H-Cube Advance reactor…………………………………… S6
3. **Synthesis of 2-chloro-1-(*p*-tolyl)propan-1-one 6**……………………… S7
4. **Synthesis of compound 7 via photo-Favorskii rearrangement**………… S7
   4.1. General procedure for the synthesis of 2-(*p*-tolyl)propanoic acid (**7**) via a photo-Favorskii rearrangement under batch conditions (GP1)……………… S7
   4.2. General procedure for the synthesis of 2-(*p*-tolyl)propanoic acid (**7**) via a photo-Favorskii rearrangement in flow (GP2)………………………… S10
   4.3. Control experiments…………………………………………………… S11
   4.4. Gram-scale reaction under continuous flow conditions………………… S11
   4.5. Comparison between batch and flow approaches………………………… S12
5. **Synthesis of compound 8**………………………………………………… S13
6. **Synthesis of Pelubiprofen 9**……………………………………………… S13
   6.1. Alternative unsuccessful synthesis of Pelubiprofen **9**……………… S14
7. **Synthesis of compound 10**………………………………………………… S16
8. **Synthesis of Loxoprofen 5**………………………………………………… S16
   8.1. Catalytic hydrogenation to Loxoprofen under batch conditions……… S16
   8.2. Catalytic hydrogenation to Loxoprofen under flow conditions………… S17
   8.3. Comparison between batch and flow approaches………………………… S17
9. **Synthesis of compound 12 via photo-Favorskii rearrangement**………… S18
   9.1. General procedure for the synthesis of 2-(9-acetyl-6-chloro-9H-carbazol-2-yl) propanoic acid (**12**) via a photo-Favorskii rearrangement in batch (GP3)……… S18
   9.2. General procedure for the synthesis of 2-(9-acetyl-6-chloro-9H-carbazol-2-yl) propanoic acid (**12**) via a photo-Favorskii rearrangement in flow (GP4)………S20
   9.3. Control experiments…………………………………………………… S20
   9.4. Comparison between batch and flow approaches………………………… S21
10. **Synthesis of Carprofen 3**………………………………………………… S21
11. **NMR spectra**……………………………………………………………… S22
12. **UV-Vis spectra**…………………………………………………………… S38
13. **References**………………………………………………………………… S39

# 1. General information

Reagents were purchased at the highest commercial quality and used as received. Carbazole intermediate **11** was kindly provided by Cosma S.p.A. and used without further purification. Anhydrous solvents were purchased in AcroSeal® bottles and used as received.

Reactions were monitored by thin layer chromatography (TLC) on Macherey-Nagel pre-coated silica gel plates (0.25 mm) and visualized by UV irradiation at 254 nm. For aryl propionic acids detection, formic acid (0.25 vol%) was added to the eluent mixture. Whenever necessary, a permanganic solution (3 g potassium permanganate, 20 g sodium carbonate, 300 mL of deionized water, sodium hydroxide 5 % solution) was used as stains for developing TLC plates. Flash chromatography was performed on standard flash column chromatography on Merck silica gel 60 (particle size: 0.04-0.063 mm). Petroleum ether (PE), hexane, ethyl acetate (EtOAc), dichloromethane (DCM), methanol (MeOH), were used as standard eluent solvents.

$^1$H-NMR spectra were recorded on spectrometers operating at 300 MHz (Bruker Avance 300), at 400 MHz (Bruker NEO400 Avance). The chemical shifts are reported in ppm (δ), with the solvent reference relative to tetramethyl silane (TMS) employed as the internal standard ($CDCl_3$ δ = 7.26 ppm, MeOD δ = 3.31 ppm, DMSO δ = 2.50 ppm). $^1$H NMR data are reported as follows: chemical shift (ppm), multiplicity (s = singlet, br s = broad singlet, d = doublet, t = triplet, q = quartet, quint = quintet, sext = sextet, hept = heptet, dd = doublet of doublets, ddd = doublet of doublets of doublets, td = triplet of doublets, qd = quartet of doublets, m = multiplet), coupling constants (Hz), and numbers of protons. $^{13}$C-NMR spectra were recorded at 25 °C on a 300 MHz, a 400 MHz, spectrometers (Bruker Avance 300, Bruker NEO400 Avance) operating at 75 or 101 MHz, with complete proton decoupling. Carbon chemical shifts are reported in ppm (δ) relative to TMS with the respective solvent resonance as the internal standard ($CDCl_3$ δ = 77.16 ppm, MeOD δ = 49.00 ppm, DMSO δ = 39.52 ppm). Structural assignments were made with additional information from gCOSY, gHSQC, gHMBC experiments. Deuterated solvents acquired from Sigma-Aldrich were used as supplied.

Mass spectra (MS) were obtained from an Advion Expression CMS. The samples were ionized in positive or negative ion mode using a ESI or APCI ionization sources. High-resolution mass spectra (HRMS) were obtained from the Unitech COSPECT centre, University of Milan, on a Synapt G2-Si HDMS (Waters) using an Acquity UPLC I-Class photodiode array (PDA) detector. The samples were ionized in positive ion mode using an ESI, ESCI or APCI ionization sources.
Gas chromatography (GC) analyses were performed on an Agilent 6890N gas chromatograph equipped with an Agilent 7683 series autosampler and coupled to an Agilent 5973N mass selective detector (MSD) operating in electron ionization (EI) mode (70 eV).

UV-Vis spectra were recorded using a Nicolet Evolution 500 (a ThermoFisher spectrophotometer) and quartz cuvettes with path length of 1 mm in acetonitrile at the appropriate concentrations, using a bandwidth of 1 nm and a scan rate of 120 nm $min^{-1}$.

Batch reactions were performed using either quartz vials (for 254 nm light irradiation) or glass vials (for 370 and 390 nm light irradiation).

Continuous-flow photochemical reactions were performed using an Asia Premium Flow Chemistry System (Syrris), equipped with high-power LEDs (365 nm, 41.8 W) and a fluoropolymer tubing as the reactor coil of 4 mL volume.

Continuous-flow hydrogenation reactions were performed using a ThalesNano H-Cube Advance reactor (distributed by StepBio), equipped with an integrated in situ hydrogen generator based on water electrolysis and a pre-packed 10 % Pd/C catalyst cartridge (CatCart® technology) with a reactor volume of 0.131 mL.

## 2. Description of the reactors

### 2.1. UVC Sankyo Denki 254 nm lamp

The Sankyo Denki lamp 253.7 nm (**Figure S1**) is a commercially available UVC lamp. The home-made lamp holder is made of stainless steel and aluminum, with an orange radiation shielding tube in PVC. The specific light intensity of the lamp (28.55 mW/cm$^2$) was determined using a compact CCD spectrometer (model CCS200/M) connected to a multimode optical fiber, purchased by Thorlabs. The distance between the lamp and the vial was calculated to be 1.3 cm using a five-position vial holder.

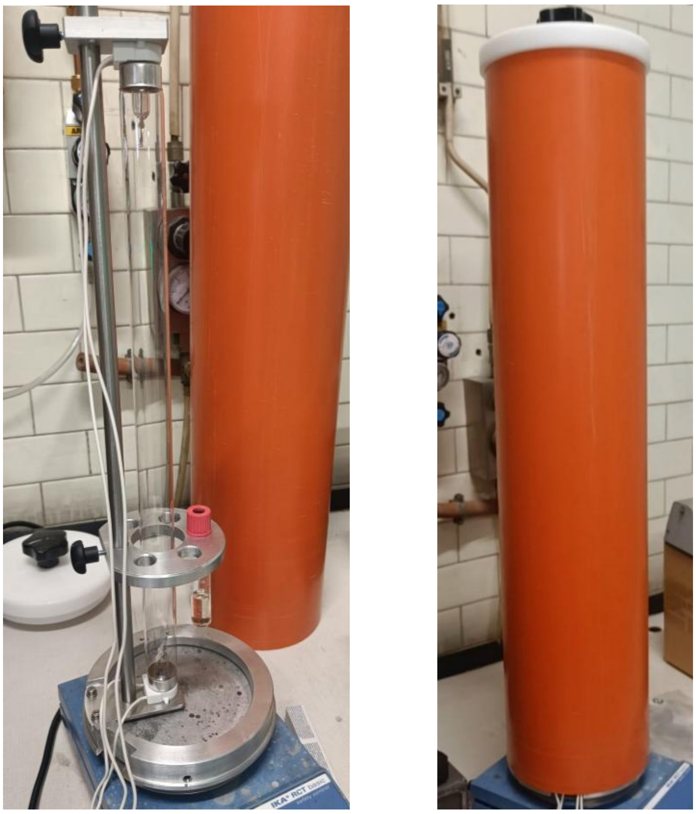

**Figure S1**: Home-made irradiation apparatus equipped with a Sankyo Denki UVC lamp.
Left: apparatus with the radiation-shielding tube removed; right: fully assembled and shielded configuration.

### 2.2. Kessil lamp set-up

The set-up used with Kessil lamps 370 Gen 2 and 390 nm is illustrated in **Figure S2**. The vial is placed 3 cm far from the source of light, above a stirrer and laterally irradiated. It is possible to cool the reaction vial by using a fan.
Lamp specifications: all the technical specifications are obtained directly from Kessil. The LEDs wavelength emission profile is 370 and 390 (± 15) nm. The power consumption is maximum 44 or 52 W, respectively, while the average intensity of the lamps is 399 mW/cm$^2$ (measured from 1 cm distance).

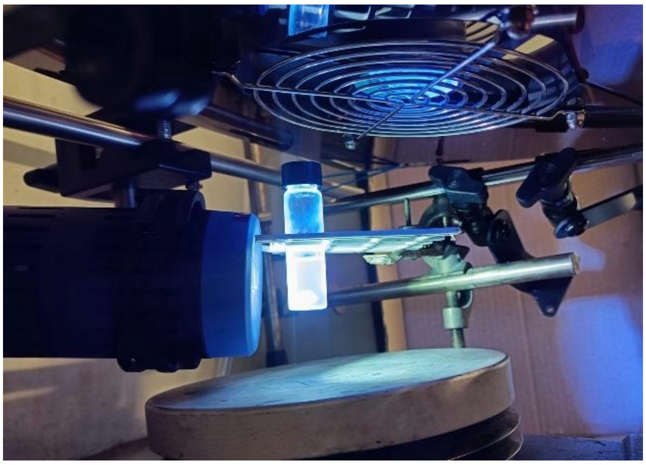

**Figure S2**: Kessil lamp set-up.

## 2.3. Asia Premium Flow Chemistry System (Syrris)

The Asia Premium Flow Chemistry System (**Figure S3**) is a Syrris reactor, equipped with high-power LEDs (365 nm, 41.8 W) and a fluoropolymer tubing as reactor coil of 4 mL volume and 0.5 mm internal diameter. The reaction mixture is fluxed from a V flask or a vial by syringes pumps at the set flow rate.

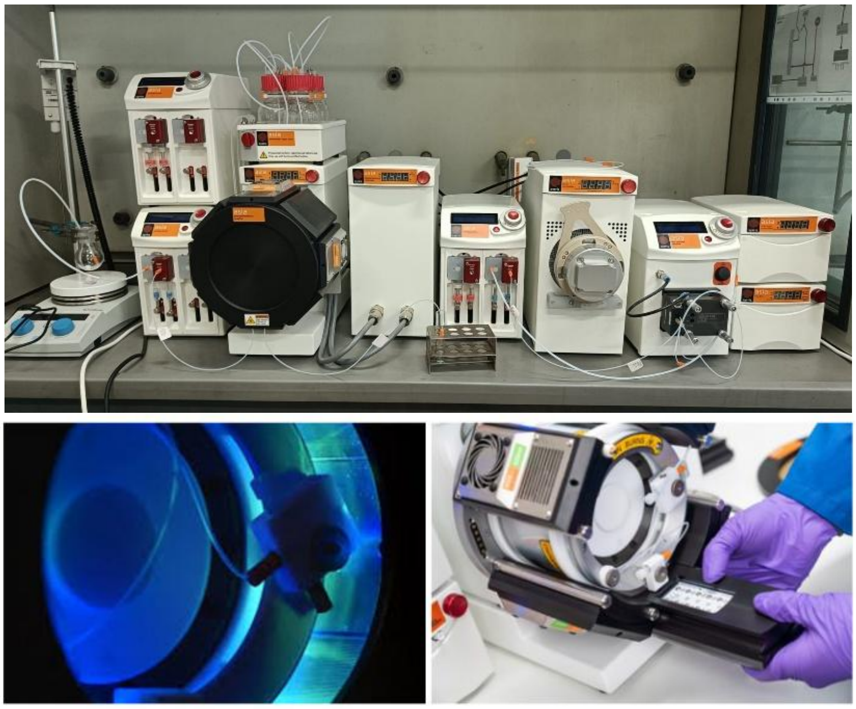

**Figure S3**: Asia Premium Flow Chemistry System.

## 2.4. ThalesNano H-Cube Advance reactor

The ThalesNano H-Cube® Advance reactor (**Figure S4**), is a compact continuous-flow hydrogenation system distributed by StepBio. It is equipped with an integrated in situ hydrogen generator based on water electrolysis and a pre-packed 10 % Pd/C catalyst cartridge (CatCart® technology) with a reactor volume of 0.131 mL. Reaction parameters including temperature, pressure, and flow rate can be precisely controlled.

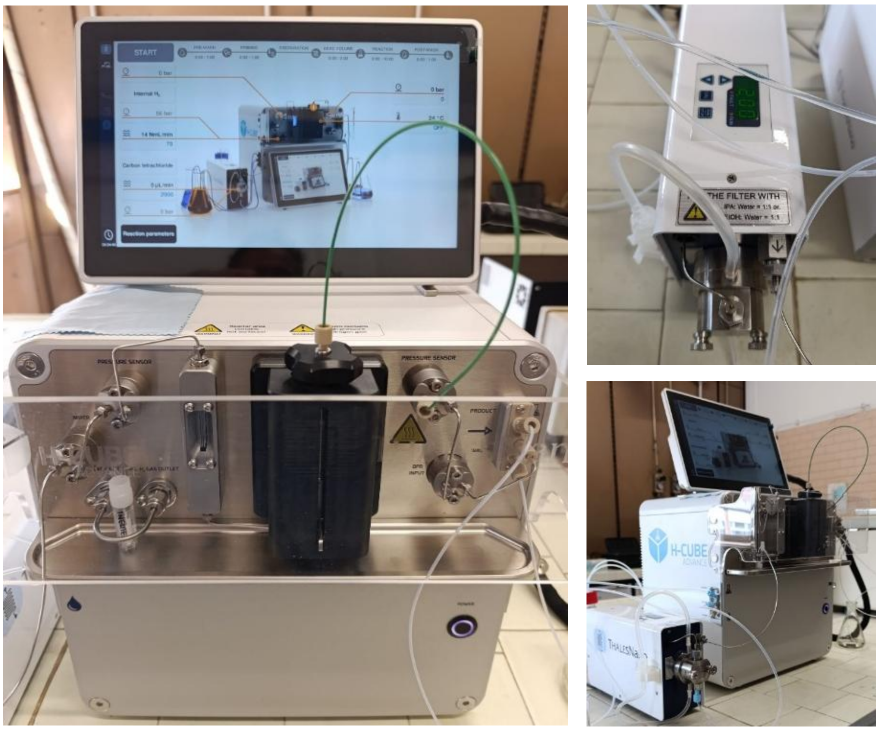


**Figure S4**: ThalesNano H-Cube Advance reactor.

## 3. Synthesis of 2-chloro-1-(*p*-tolyl)propan-1-one 6

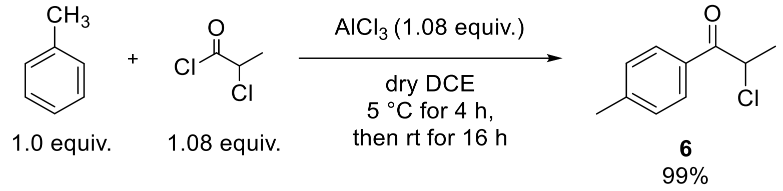


Compound **6** was synthesized according to a literature procedure.[1]

Under a nitrogen atmosphere, to a stirred pale-yellow suspension of $AlCl_3$ (7.2 g, 54 mmol, 1.08 equiv.) in anhydrous DCE (27.5 mL), maintained at 0-5 °C, were sequentially added toluene (5.31 mL, 50 mmol, 1.00 equiv.) and a solution of 2-chloropropionyl chloride (5.24 mL, 54 mmol, 1.08 equiv.) in anhydrous DCE (10 mL), resulting in a substrate concentration of 1.33 M. The colour of the reaction mixture changed from light yellow to red. The mixture was stirred at 5-15 °C for 4 h and then at room temperature for an additional 16 h. After that time, the chilled reaction mixture was quenched with ice and HCl 4 M (25 mL), and diluted with DCM (30 mL) and water (35 mL). The organic phase was then washed with water (3 × 70 mL) and brine (1 × 70mL), dried over anhydrous sodium sulphate and concentrated under reduced pressure to give 2-chloro-1-(*p*-tolyl)propan-1-one **6** in 99% yield as a brown oil (9.04 g). All the analytical data are in accordance with the literature.[2]

**$^1$H NMR** (300 MHz, $CDCl_3$) δ 7.92 (d, $J$ = 8.3 Hz, 2H), 7.29 (d, $J$ = 8.2 Hz, 2H), 5.24 (q, $J$ = 6.7 Hz, 1H), 2.43 (s, 3H), 1.74 (d, $J$ = 6.7 Hz, 3H).

## 4. Synthesis of compound 7 via photo-Favorskii rearrangement

### 4.1. General procedure for the synthesis of 2-(*p*-tolyl)propanoic acid (7) via a photo-Favorskii rearrangement under batch conditions (GP1)

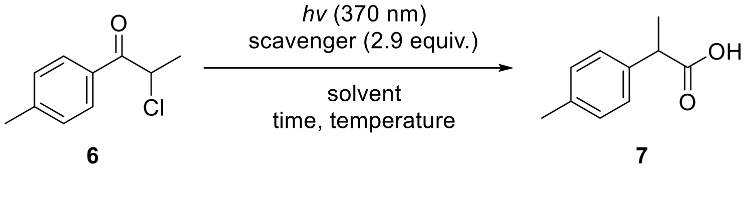


A 10 mL vial was charged with 2-chloro-1-(*p*-tolyl)propan-1-one **6** (73 mg, 0.4 mmol, 0.1 M) and the scavenger (typically propylene oxide, 80 µL, 1.14 mmol, 2.9 equiv.) and dissolved in the desired solvent (typically 9:1 solvent mixture of acetone/water, 4 mL). The yellowish reaction mixture was irradiated using a 370 nm Kessil lamp for the desired time at the desired temperature (typically 1.5 h, 45 °C). After that, the reaction mixture was concentrated under reduced pressure. Dibromomethane (5 µL) was added as the internal standard to the resulting crude for the $^1$H-NMR yield. 2-(*p*-tolyl)propanoic acid **7** was obtained by column chromatography on silica gel (hexane/ethyl acetate from 9:1 to 7:3 + 0.25 vol% formic acid) in excellent yields (up to 83%) as a yellowish solid (up to 54.5 mg). All the analytical data are in agreement with the literature.[3]

**Rf** = 0.38 (hexane/EtOAc 7:3 + 0.25 vol% of formic acid). **$^1$H NMR** (300 MHz, $CDCl_3$) δ 7.24 (d, $J$ = 8.1 Hz, 2H), 7.17 (d, $J$ = 7.9 Hz, 2H), 3.73 (q, $J$ = 7.1 Hz, 1H), 2.36 (s, 3H), 1.53 (dd, $J$ = 7.2, 1.6 Hz, 3H). **$^{13}$C NMR** (75 MHz, $CDCl_3$) δ 181.2, 137.2, 136.9, 129.50 (2C), 127.59 (2C), 45.1, 21.2, 18.2. **GC-MS (EI)** m/z: [M] Calcd for $C_{10}H_{12}O_2$ 164.08; Found 164.09, mass error +60.95 ppm.

### 4.1.1 REACTION TIME SCREENING

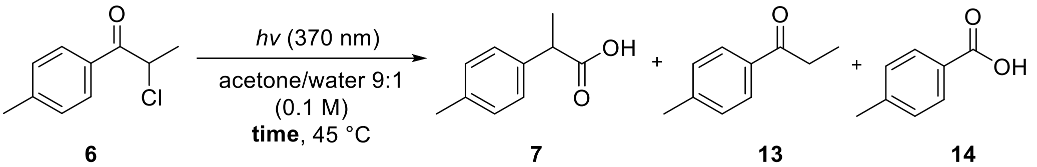


| ENTRY | TIME (h) | 7, ¹H-NMR YIELD (%)* | 13, ¹H-NMR YIELD (%)* | 14, ¹H-NMR YIELD (%)* |
|---|---|---|---|---|
| **1** | 4 | 52 | 20 | - |
| **2** | 3 | 50 | 21 | - |
| **3** | 1.5 | 51 (50) | 20 | - |
| **4** | 1 | 52 | 21 | 5 |

* yields determined by ¹H NMR using dibromomethane as internal standard. Isolated yields are in brackets.

**Table S1**: Time screening for the photo-Favorskii rearrangement on 2-chloro-1-(*p*-tolyl)propan-1-one **6**.

### 4.1.2 TEMPERATURE SCREENING

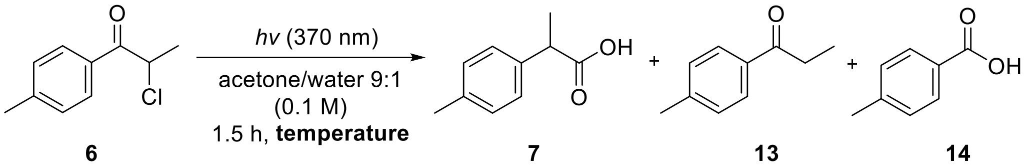


| ENTRY | TEMPERATURE (°C) | 7, ¹H-NMR YIELD (%)* | 13, ¹H-NMR YIELD (%)* | 14, ¹H-NMR YIELD (%)* |
|---|---|---|---|---|
| **1** | 45 | 51 (50) | 20 | - |
| **2** | 25 | 50 | 22 | - |

* yields determined by ¹H NMR using dibromomethane as internal standard. Isolated yields are in brackets.

**Table S2**: Temperature screening for the photo-Favorskii rearrangement on 2-chloro-1-(*p*-tolyl)propan-1-one **6**.

### 4.1.3 LAMP INTENSITY SCREENING

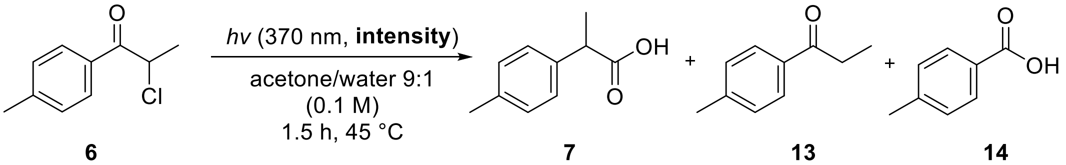


| ENTRY | LAMP INTENSITY (%) | 7, ¹H-NMR YIELD (%)* | 13, ¹H-NMR YIELD (%)* | 14, ¹H-NMR YIELD (%)* |
|---|---|---|---|---|
| **1** | 100 | 51 (50) | 20 | - |
| **2** | 50 | 42 | 18 | 31 |

* yields determined by ¹H NMR using dibromomethane as internal standard. Isolated yields are in brackets.

**Table S3**: Lamp intensity screening for the photo-Favorskii rearrangement on 2-chloro-1-(p-tolyl)propan-1-one **6**.

### 4.1.4 SOLVENT SCREENING AND WATER CONTENT EVALUATION

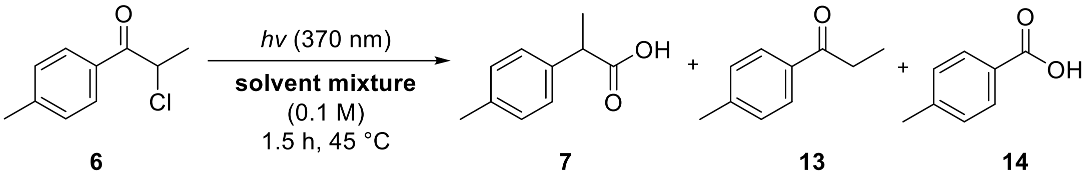


6 7 13 14

| ENTRY | SOLVENT MIXTURE | 7, $^1$H-NMR YIELD (%)* | 13, $^1$H-NMR YIELD (%)* | 14, $^1$H-NMR YIELD (%)* |
|---|---|---|---|---|
| **1** | acetone/water 9:1 | 51 (50) | 20 | - |
| **2** | acetonitrile/water 9:1 | 38 | 15 | 28 |
| **3** | acetone/water 3:1 | 27 | n.a. | 5 |
| **4** | acetonitrile/water 3:1 | 26 | 20 | 24 |

* yields determined by $^1$H NMR using dibromomethane as internal standard. Isolated yields are in brackets.

**Table S4**: Solvent mixture screening for the photo-Favorskii rearrangement on 2-chloro-1-(*p*-tolyl)propan-1-one **6.**

### 4.1.5 MOLARITY SCREENING

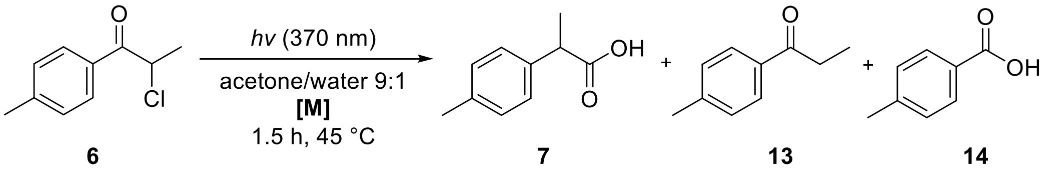


6 7 13 14

| ENTRY | MOLARITY (M) | 7, $^1$H-NMR YIELD (%)* | 13, $^1$H-NMR YIELD (%)* | 14, $^1$H-NMR YIELD (%)* |
|---|---|---|---|---|
| **1** | 0.2 | 41 | 33 | 5 |
| **2** | 0.1 | 51 (50) | 20 | - |
| **3** | 0.05 | 59 | 15 | - |

* yields determined by $^1$H NMR using dibromomethane as internal standard. Isolated yields are in brackets.

**Table S5**: Molarity screening for the photo-Favorskii rearrangement on 2-chloro-1-(*p*-tolyl)propan-1-one **6.**

### 4.1.6 SCAVENGER EVALUATION

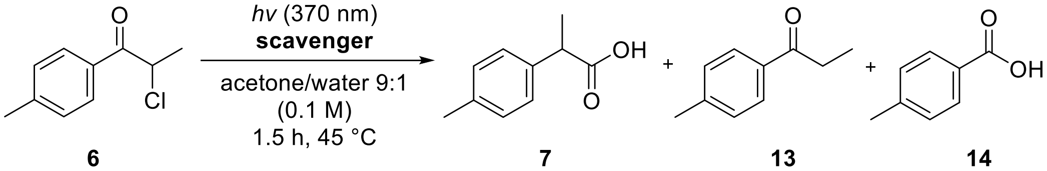


6 7 13 14

| ENTRY | SCAVENGER | 7, $^1$H-NMR YIELD (%)* | 13, $^1$H-NMR YIELD (%)* | 14, $^1$H-NMR YIELD (%)* |
|---|---|---|---|---|
| **1** | - | 51 (50) | 20 | - |
| **2** | propylene oxide (2.9 equiv.) | **84 (83)** | 4 | 8 |

* yields determined by $^1$H NMR using dibromomethane as internal standard. Isolated yields are in brackets.

**Table S6**: Evaluation of the scavenger use for the photo-Favorskii rearrangement on 2-chloro-1-(*p*-tolyl)propan-1-one **6.**

## 4.2. General procedure for the synthesis of 2-(*p*-tolyl)propanoic acid (**7**) via a photo-Favorskii rearrangement in flow (GP2)

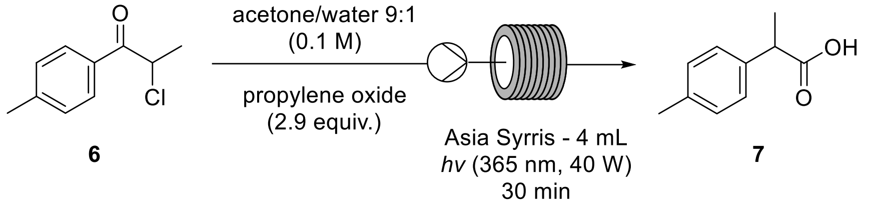


A vial was charged with 2-chloro-1-(*p*-tolyl)propan-1-one **6** (73 mg, 0.4 mmol, 0.1 M) and propylene oxide (80 µL, 1.14 mmol, 2.9 equiv.) and dissolved in a 9:1 mixture of acetone/water (4 mL). The yellowish reaction mixture was fed inside the coil reactor (0.5 mm ID, total volume of 4 mL) at the desired flow rate (typically 133 µL· min$^{-1}$, corresponding to 30 min as residence time) and irradiated with 365 nm LEDs using the Asia Syrris System at the desired temperature. After the irradiation, the reaction mixture was concentrated under reduced pressure. Dibromomethane (5 µL) was added as the internal standard to the resulting crude to calculate the $^{1}$H-NMR yield. 2-(*p*-tolyl)propanoic acid **7** was obtained by column chromatography on silica gel (hexane/ethyl acetate from 9:1 to 7:3, adding 0.25 vol% formic acid) in good yields (up to 86%) as a yellowish solid (up to 56.5 mg). All the analytical data are in agreement with the literature.[3]

**Rf** = 0.38 (hexane/EtOAc 7:3 + 0.25 vol% of formic acid). **$^{1}$H NMR** (300 MHz, $CDCl_3$) δ 7.24 (d, *J* = 8.1 Hz, 2H), 7.17 (d, *J* = 7.9 Hz, 2H), 3.73 (q, *J* = 7.1 Hz, 1H), 2.36 (s, 3H), 1.53 (dd, *J* = 7.2, 1.6 Hz, 3H). **$^{13}$C NMR** (75 MHz, $CDCl_3$) δ 181.1, 137.1, 136.8, 129.40 (2C), 127.50 (2C), 45.0, 21.1, 18.1. **GC-MS (EI)** m/z: [M] Calcd for $C_{10}H_{12}O_2$ 164.08; Found 164.09, mass error +60.95 ppm.

### 4.2.1 RESIDENCE TIME SCREENING AT 45 °C

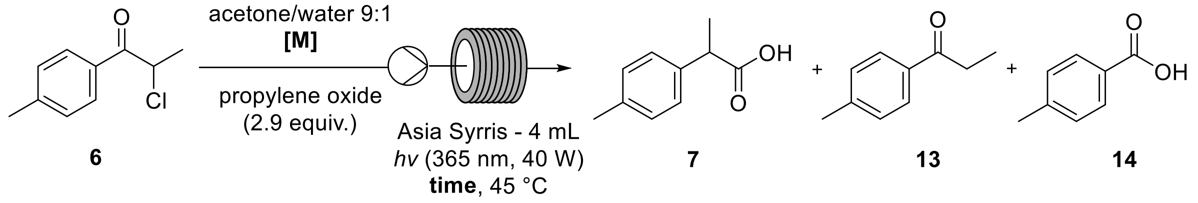


| ENTRY | RESIDENCE TIME (min) | MOLARITY (M) | 7, $^{1}$H-NMR YIELD (%)* | 13, $^{1}$H-NMR YIELD (%)* | 14, $^{1}$H-NMR YIELD (%)* |
|---|---|---|---|---|---|
| **1** | 30 | 0.1 | **94 (86)** | - | - |
| **2** | **15** | 0.1 | 71 | 24 | - |
| **3** | 30 | **0.2** | n.a. | n.a. | n.a. |

*yields determined by $^{1}$H NMR using dibromomethane as internal standard. Isolated yields are in brackets.

**Table S7**: Photo-Favorskii rearrangement optimization of 2-chloro-1-(*p*-tolyl)propan-1-one **6** under flow conditions.

### 4.2.2 RESIDENCE TIME SCREENING AT 25 °C

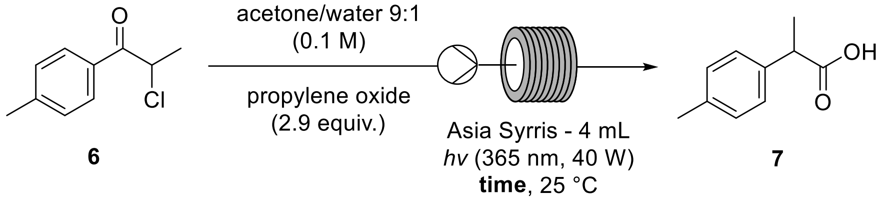


| ENTRY | RESIDENCE TIME (min) | 7, $^1$H-NMR YIELD (%)* | PRODUCTIVITY (mg·h$^{-1}$) |
|---|---|---|---|
| **1** | 30 | 73 (71) | 96 |
| **2** | 15 | 70 | 184 |
| **3** | 10 | 68 | 268 |
| **4** | 5 | **62** | **490** |

*yields determined by $^1$H NMR using dibromomethane as internal standard. Isolated yields are in brackets.

**Table S8**: Photo-Favorskii rearrangement optimization of 2-chloro-1-(*p*-tolyl)propan-1-one **6** to obtain arylpropionic acid **7** at 25 °C under UVA irradiation and continuous flow conditions.

## 4.3. Control experiments

Two control experiments were conducted in the absence of light at 25 and 45 °C, recovering only compound **6** and confirming that UV irradiation is required for the photochemical rearrangement.

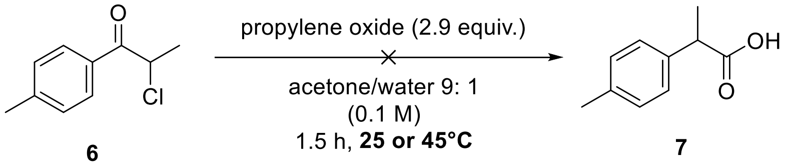


## 4.4. Gram-scale reaction under continuous flow conditions

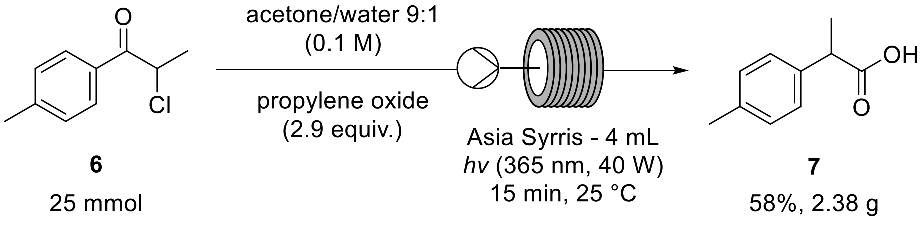


A V-flask was charged with 2-chloro-1-(*p*-tolyl)propan-1-one **6** (4.566 g, 25 mmol, 0.1 M) and propylene oxide (5 mL, 72.5 mmol, 2.9 equiv.) and dissolved in a 9:1 solvent mixture of acetone/water (250 mL). The yellowish reaction mixture was irradiated with 365 nm LEDs using the Asia Syrris System at 25 °C. The parameters were the following: 0.5 mm for the internal diameter, a reactor volume of 4 mL, 15 minutes as residence time and 267 µL · min$^{-1}$ was the set flow rate. After the irradiation, the reaction mixture was concentrated under reduced pressure, diluted with ethyl acetate (150 mL) and extracted using a solution 0.125 M of sodium bicarbonate (150 mL). HCl 4 M was added dropwise to the aqueous phase until pH~2 and the desired product was extracted with ethyl acetate (2 × 150 mL) from the aqueous phase. 2-(*p*-tolyl)propanoic acid **7** was obtained in 58% yield as a yellowish solid (2.38 g). All the analytical data are in agreement with the literature.[3]

**Rf** = 0.38 (hexane/EtOAc 7:3 + 0.25 vol% of formic acid). **$^1$H NMR** (300 MHz, $CDCl_3$) δ 7.24 (d, *J* = 8.1 Hz, 2H), 7.17 (d, *J* = 7.9 Hz, 2H), 3.73 (q, *J* = 7.1 Hz, 1H), 2.36 (s, 3H), 1.53 (dd, *J* = 7.2, 1.6 Hz, 3H). **$^{13}$C NMR** (75 MHz, $CDCl_3$) δ 181.1, 137.1, 136.8, 129.40 (2C), 127.50 (2C), 45.0, 21.1, 18.1. **GC-MS (EI)** m/z: [M] Calcd for $C_{10}H_{12}O_2$ 164.08; Found 164.09, mass error +60.95 ppm.

## 4.5. Comparison between batch and flow approaches

The performances of the experiments under continuous flow conditions were evaluated in terms of productivity and space-time yield. The productivity is defined as the amount of product per hour provided by the system, while the space-time yield (STY) as the amount of product per hour, per unit of volume.

$$\boldsymbol{Productivity} = \frac{\text{mol limiting reagent} \cdot \text{yield} \cdot \text{MW product}}{\text{time (h)}}$$

$$\boldsymbol{STY} = \frac{\text{mol limiting reagent} \cdot \text{yield} \cdot \text{MW product}}{\text{time (h)} \cdot \text{volume (L)}}$$

The productivity and the space-time yield for the photo-Favorskii rearrangement for both batch and continuous flow protocols were calculated considering the same substrate amount (0.4 mmol), concentration and solvent composition (9:1 solvent mixture of acetone/water at a concentration of 0.1 M), under UVA irradiation as illustrated in **Table S9**.

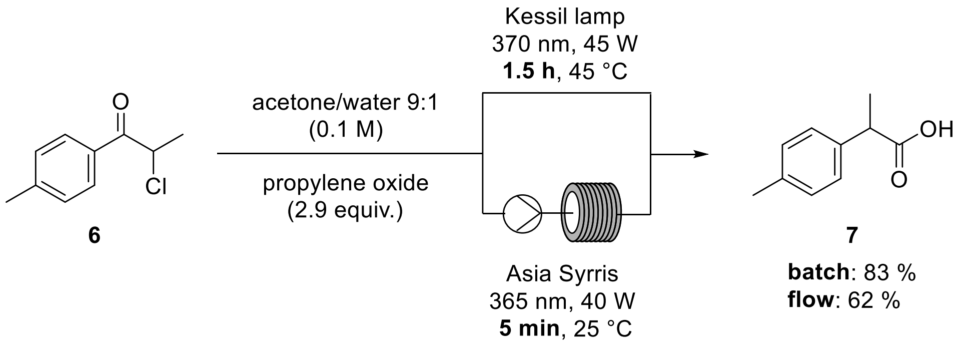


| | YIELD (%)* | TIME (min) | PRODUCTIVITY (mg · h$^{-1}$) | PRODUCTIVITY RELATIVE FACTOR | STY (g · h$^{-1}$ · L$^{-1}$) | STY RELATIVE FACTOR |
|---|---|---|---|---|---|---|
| **BATCH** | 84 | 90 | 36 | 1 | 9 | 1 |
| **FLOW** | 62 | 5 | 490 | **14** | 123 | **14** |

*yields determined by $^1$H NMR using dibromomethane as internal standard. Isolated yields are in brackets.

**Table S9:** Comparison between batch and flow conditions for the photo-Favorskii rearrangement on compound **6** to obtain aryl propionic acid **7**.

For the **gram-scale reaction**, the productivity was calculated to be 153 mg · h$^{-1}$; the space-time yield (STY) was calculated to be 38 g · h$^{-1}$ · L$^{-1}$.

## 5. Synthesis of compound 8

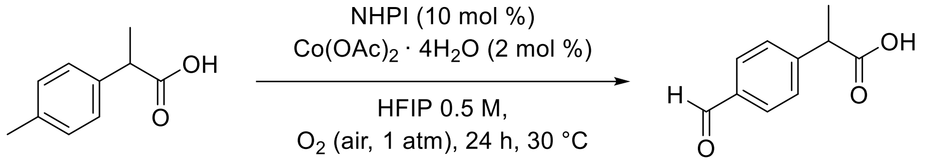


**7** **8** 68%

According to a modified literature procedure,[4] a round bottom flask was charged with 2-(*p*-tolyl)propanoic acid **7** (651 mg, 3.97 mmol, 1 equiv., 0.5 M in HFIP), NHPI (65 mg, 0.4 mmol, 0.1 equiv.), cobalt acetate tetrahydrate (19.8 mg, 0.08 mmol, 0.02 equiv.) and the reagents were dissolved in HFIP (8 mL) prior to sonicating the pink-orange reaction mixture. The reaction was stirred in the open air, at 30 °C for 24 hours. The solvent was then removed under reduced pressure and the product was obtained after purification through chromatographic column on silica gel (hexane/ethyl acetate from 85:15 to 75:25, adding 0.25 vol% of formic acid). 2-(4-formylphenyl)propanoic acid **8** was obtained in 68% yield as a colourless oil which solidified upon standing as a yellowish solid (481 mg).

**Rf** = 0.39 (hexane/ethyl acetate 4:6 + 0.25 vol% of formic acid). **$^1$H NMR** (300 MHz, $CDCl_3$) δ 9.99 (s, 1H), 7.85 (d, *J* = 8.1 Hz, 2H), 7.49 (d, *J* = 8.1 Hz, 2H), 3.84 (q, *J* = 7.1 Hz, 1H), 1.55 (d, *J* = 7.1 Hz, 3H). **$^{13}$C NMR** (75 MHz, $CDCl_3$) δ 192.0, 179.4, 146.7, 135.8, 130.29 (2C), 128.56 (2C), 45.7, 18.2. **HRMS (ESI-TOF)** *m/z* calcd for $C_{10}H_{11}O_3^+$: 179.0703 $[M + H]^+$; found: 179.0712, mass error +5.02 ppm; *m/z* calcd for $C_9H_9O^\bullet$: 133.0653 $[M - CO_2]^\bullet$; found: 133.0656, mass error +2.25 ppm.

## 6. Synthesis of Pelubiprofen 9

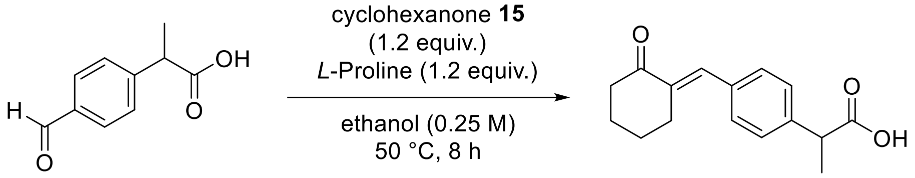


**8** **9** (Pelubiprofen) 61%

According to a modified literature procedure,[5] to a stirring mixture of **8** (89 mg, 0.5 mmol, 1 equiv., 0.25 M in ethanol) and *L*-proline (69 mg, 0.6 mmol, 1.2 equiv.) in anhydrous EtOH (2 mL) was added cyclohexanone **15** (62 µL, 0.6 mmol, 1.2 equiv.). The reaction mixture was stirred at 50 ºC for 8h. The solvent was then evaporated and the crude was purified by flash column chromatography on silica gel (hexane/ethyl acetate from 85:15 to 75:25, adding 0.2 vol% of formic acid). (*E*)-2-(4-((2-oxocyclohexylidene)methyl)phenyl)propanoic acid **9** (Pelubiprofen) was isolated in 61% yield as a pale yellow oil (79 mg). All the analytical data are in agreement with the literature.[6]

**Rf** = 0.39 (hexane/ethyl acetate 4:6 + 0.25 vol% of formic acid). **$^1$H NMR** (300 MHz, $CDCl_3$) δ 7.46 (s, 1H), 7.41 – 7.28 (m, 4H), 3.75 (q, J = 7.1 Hz, 1H), 2.82 (td, J = 6.4, 2.1 Hz, 2H), 2.53 (t, J = 6.7 Hz, 2H), 1.93 (p, J = 6.4 Hz, 2H), 1.76 (p, J = 6.2 Hz, 2H), 1.52 (d, J = 7.1 Hz, 3H). **$^{13}$C NMR** (75 MHz, $CDCl_3$) δ 202.0, 178.8, 140.4, 136.9, 135.3, 134.9, 130.81 (2C), 127.77 (2C), 45.2, 40.5, 29.1, 24.0, 23.5, 18.2.

## 6.1. Alternative unsuccessful synthesis of Pelubiprofen **9**

A reverse order of the reactions was also evaluated by introducing the α,β-unsaturated moiety before performing the photo-Favorskii rearrangement to obtain Pelubiprofen **9**. Therefore, the corresponding α-chloro and acetoxy- derivatives, respectively compounds **B** and **B'**, were prepared and subjected to a photo-Favorskii rearrangement, following the previously reported GP1. However, the resulting conjugated substrates proved unreactive under irradiation conditions, and the starting material was recovered unchanged in both the experiments.

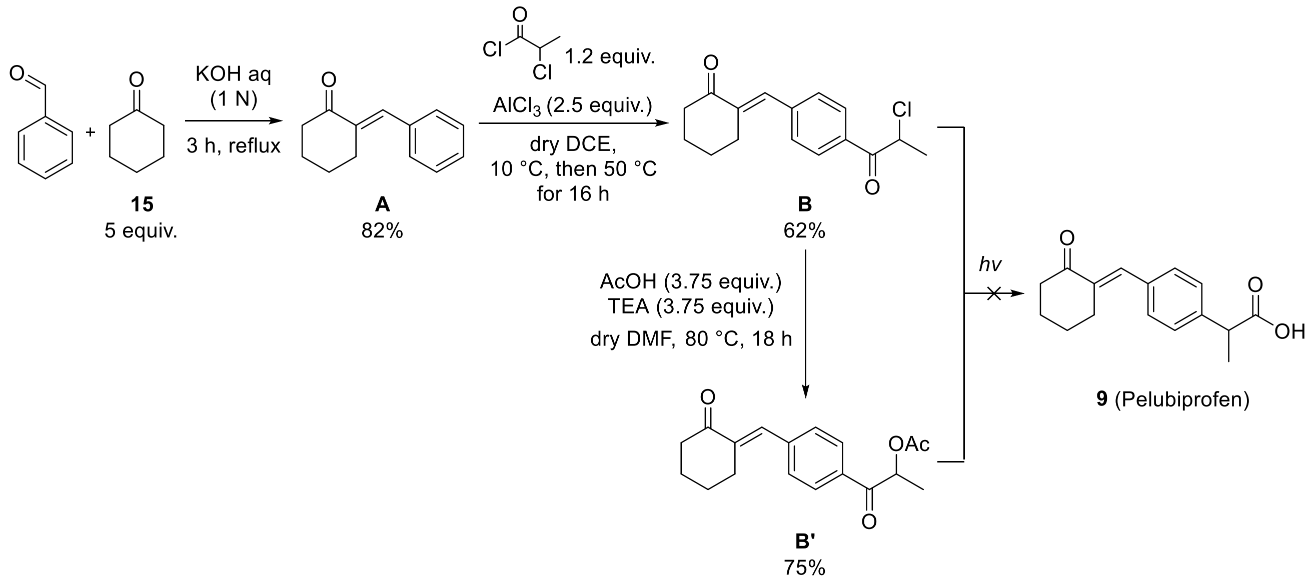


**Synthesis of (*E*)-2-benzylidenecyclohexan-1-one (A)**

According to a literature procedure,[7] a mixture of benzaldehyde (1 mL, 10 mmol, 1 equiv.), cyclohexanone **15** (5 mL, 50 mmol, 5 equiv.), and 1M KOH aqueous solution (12 mL) was refluxed for 3 h. After cooling to rt, diethyl ether (20 mL) was added, and the aqueous layer was extracted with ether (3 × 8 mL). The combined organic layers were then washed to neutrality with water (2 × 40 mL), dried over anhydrous $Na_2SO_4$ and concentrated under reduced pressure to give a yellow oil. The resulting crude was chromatographed on silica gel (PE/EtOAc from 97:3 to 96:4. (*E*)-2-benzylidenecyclohexan-1-one **A** was obtained in 82% yield as a yellow oil (1.53 g). All the analytical data are in agreement with the literature.[8]

**Rf** = 0.31 (hexane/ethyl acetate 92:8). **$^1$H NMR** (300 MHz, $CDCl_3$) δ 7.50 (t, *J* = 2.3 Hz, 1H), 7.43 – 7.28 (m, 5H), 2.84 (td, *J* = 6.4, 2.3 Hz, 2H), 2.54 (t, *J* = 6.7 Hz, 2H), 1.93 (p, *J* = 6.5, Hz, 2H), 1.77 (p, *J* = 6.2 Hz, 2H).

**Synthesis of (*E*)-2-(4-(2-chloropropanoyl)benzylidene)cyclohexan-1-one (B)**

According to a modified literature procedure,[1] under nitrogen atmosphere, to a stirred pale-yellow suspension of $AlCl_3$ (2.6 g, 19.5 mmol, 2.5 equiv.) in anhydrous DCE (6 mL), maintained at 10-15 °C, were sequentially added a solution of 2-chloro-propionyl chloride (908 µL, 9.36 mmol, 1.2 equiv.) in anhydrous DCE (2 mL) and a solution of (*E*)-2-benzylidenecyclohexan-1-one **A** (1.45 g, 7.8 mmol, 1 equiv.) in anhydrous DCE (2 mL), resulting in a substrate concentration of 0.975 M. The colour of the reaction mixture changed immediately from yellowish to red and then to brown. The mixture was stirred at 50 °C for 16 h, monitoring the reaction by TLC (hexane: ethyl acetate = 92:8) and by GC-MS. The chilled reaction mixture was quenched with ice, HCl 4M (9 mL) and diluted with DCM (10 mL) and water (15 mL). The organic phase was then washed with water

(3 × 20 mL) and brine (1 × 20 mL), dried over anhydrous sodium sulphate and concentrated under reduced pressure to give a brown oil. The crude was purified through column chromatography on silica gel (PE/EtOAc from 92:8 to 88:12). (*E*)-2-(4-(2-chloropropanoyl)benzylidene)cyclohexan-1-one **B** was obtained in 62% yield as a yellow oil (1.338 g).

**Rf** = 0.31 (hexane/ethyl acetate 8:2). **$^1$H NMR** (300 MHz, $CDCl_3$) δ 8.02 (dd, *J* = 8.5, 4.8 Hz, 2H), 7.64 – 7.35 (m, 3H), 5.29 – 5.17 (m, 1H), 2.84 (td, *J* = 6.4, 2.3 Hz, 2H), 2.56 (t, *J* = 6.7 Hz, 2H), 2.01 – 1.90 (m, 2H), 1.84 – 1.72 (m, 5H). **$^{13}$C NMR** (75 MHz, $CDCl_3$) δ 201.8, 193.1, 141.1, 139.1, 133.9, 133.5, 130.47 (2C), 129.11 (2C), 52.9, 40.5, 29.2, 23.9, 23.5, 20.0. **MS (ESI+)** m/z: $[M+H]^+$ calcd for $C_{16}H_{18}ClO_2^+$ 277.10; found 277.67, mass error +2057.02 ppm.

**Synthesis of (*E*)-1-oxo-1-(4-((2-oxocyclohexylidene)methyl)phenyl)propan-2-yl acetate (B')**

According to a procedure provided by Cosma S.p.A., under inert atmosphere, a double-neck flask was charged with acetic acid (504 µL, 8.8 mmol, 3.75 equiv.) and dry DMF (1.3 mL). Triethylamine (TEA, 1.15 mL, 8.23 mmol, 3.5 equiv.) was added dropwise to the resulting solution. Then, a solution of (*E*)-2-(4-(2-chloropropanoyl)benzylidene)cyclohexan-1-one **B** (650 mg, 2.35 mmol, 1 equiv., 0.2 M in DMF) in dry DMF (10.4 mL) was added to the stirring mixture. The reaction mixture was stirred at 80 °C for 5 h under static nitrogen atmosphere, then monitored by TLC (hexane/ethyl acetate 1:1) and stirred for further 13 h. After cooling to rt, DMF was removed by distillation. Then, the resulting crude was dissolved in ethyl acetate and the TEA salts were removed by a Buchner filtration. The filtrate was then concentrated under reduced pressure and the resulting residue was partitioned between EtOAc (20 mL) and water (20 mL). The organic phase was washed with water and brine (20 mL), dried over anhydrous sodium sulphate, filtered and the solvent removed under reduced pressure to give a yellow solid. The crude was purified by column (hexane/ethyl acetate from 9:1 to 8:2) affording (*E*)-1-oxo-1-(4-((2-oxocyclohexylidene) methyl)phenyl)propan-2-yl acetate **B'** in 75% yield as a yellow oil (526 mg).

**Rf** = 0.16 (hexane/ethyl acetate 8:2). **$^1$H NMR** (300 MHz, $CDCl_3$) δ 7.96 – 7.89 (m, 2H), 7.49 – 7.41 (m, 3H), 5.91 (q, *J* = 7.0 Hz, 1H), 2.80 (td, *J* = 6.4, 2.2 Hz, 2H), 2.52 (t, *J* = 6.7 Hz, 2H), 2.11 (s, 3H), 1.92 (p, *J* = 6.5 Hz, 2H), 1.75 (p, *J* = 6.3 Hz, 2H), 1.50 (d, *J* = 7.0 Hz, 3H). **$^{13}$C NMR** (75 MHz, $CDCl_3$) δ 201.6, 196.3, 170.5, 140.9, 134.0, 134.0, 133.8, 130.43 (2C), 128.49 (2C), 71.5, 40.4, 29.1, 23.8, 23.4, 20.8, 17.2, 17.1. **MS (ESI+)** m/z: $[M+Na]^+$ calcd for $C_{18}H_{20}O_4Na^+$ 323.13; found 323.59, mass error +1423.58 ppm.

# 7. Synthesis of compound 10

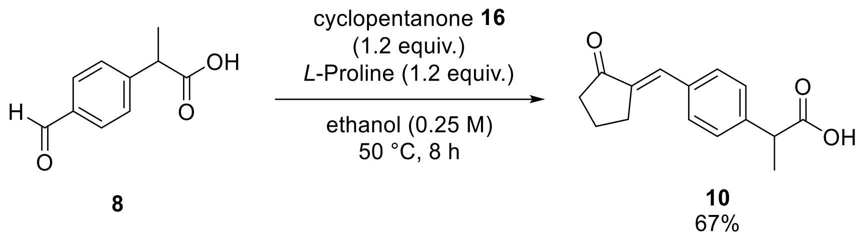


According to a modified literature procedure,[5] to a stirring mixture of **8** (89 mg, 0.5 mmol, 1 equiv., 0.25 M in ethanol) and *L*-proline (69 mg, 0.6 mmol, 1.2 equiv.) in anhydrous EtOH (2 mL) was added cyclopentanone **16** (53 µL, 0.6 mmol, 1.2 equiv.). The reaction mixture was stirred at 50 ºC for 8 h. The solvent was then evaporated and the crude was purified by flash column chromatography on silica gel (hexane/ethyl acetate from 85:15 to 75:25, adding 0.2 vol% of formic acid). Compound **10** was isolated in 67% yield as a pale yellow oil which solidified upon standing (82 mg). All the analytical data are in agreement with the literature.[6]

**Rf** = 0.42 (hexane/ethyl acetate 6:4 + 0.25 vol% of formic acid). **$^1$H NMR** (400 MHz, $CDCl_3$) δ 7.50 (d, *J* = 8.1 Hz, 2H), 7.37 (d, *J* = 8.3 Hz, 3H), 3.77 (q, *J* = 7.2 Hz, 1H), 2.96 (td, *J* = 7.2, 2.7 Hz, 2H), 2.41 (t, *J* = 7.9 Hz, 2H), 2.03 (p, *J* = 7.6 Hz, 2H), 1.53 (d, *J* = 7.2 Hz, 3H). **$^{13}$C NMR** (101 MHz, CDCl3) δ 208.5, 179.7, 141.2, 136.3, 134.9, 132.0, 130.97 (2C), 128.14 (2C), 45.3, 37.9, 29.5, 20.3, 18.1.

# 8. Synthesis of Loxoprofen 5

## 8.1 Catalytic hydrogenation to Loxoprofen under batch conditions

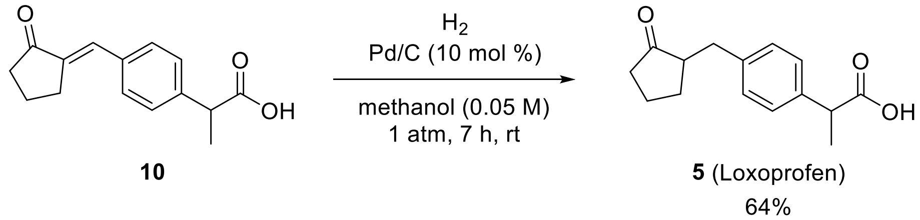


A solution of compound **10** (122 mg, 0.5 mmol, 1 equiv., 0.05 M in methanol) in methanol (10 mL) was treated with Pd/C 10% (53 mg, 0.05 mmol of Pd, 0.1 equiv.) and hydrogenated at 1 atm. After stirring for 7 h at 25 °C, the reaction mixture was filtered through a Celite pad and concentrated under reduced pressure. The resulting crude was chromatographed on silica gel (hexane/ethyl acetate from 82:18 to 76:24, adding 0.1 vol% of formic acid) to obtain Loxoprofen **5** as a colourless oil in 64% yield (79 mg). All the analytical data are in agreement with the literature.[6]

**Rf** = 0.43 (hexane/ethyl acetate 6:4 + 0.25 vol% of formic acid). **$^1$H NMR** (400 MHz, $CDCl_3$) δ 7.23 (d, *J* = 8.1 Hz, 2H), 7.12 (d, *J* = 8.1 Hz, 2H), 3.71 (q, *J* = 7.2 Hz, 1H), 3.12 (dd, *J* = 13.9, 4.1 Hz, 1H), 2.50 (dd, *J* = 13.9, 9.5 Hz, 1H), 2.41 – 2.24 (m, 2H), 2.16 – 2.03 (m, 2H), 2.02 – 1.87 (m, 1H), 1.84 – 1.62 (m, 1H), 1.61 – 1.42 (m, 4H). **$^{13}$C NMR** (101 MHz, $CDCl_3$) δ 220.5, 180.1, 139.3, 137.8, 129.34 (2C), 127.78 (2C), 51.1, 45.0, 38.3, 35.3, 29.4, 20.7, 18.3.

## 8.2 Catalytic hydrogenation to Loxoprofen under flow conditions

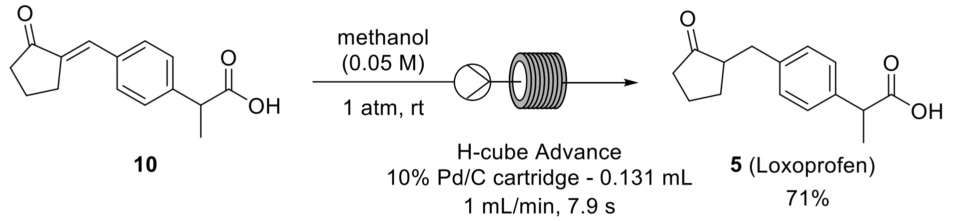


A solution of compound **10** (122 mg, 0.5 mmol, 1 equiv., 0.05 M in MeOH) in methanol (10 mL) was pumped through a ThalesNano H-Cube Advance reactor in full hydrogen mode equipped with a pre-packed 10 % Pd/C CatCart cartridge with a volume of 0.131 mL, setting a flow rate of 1 mL$\cdot$ min$^{-1}$ and a residence time of 7.9 s. The reaction mixture was pumped through the reactor for 10 min, at rt and 1 atm. Reaction progress was monitored by HPLC using a Zorbax Eclipse C18 column and a binary gradient elution system (ACN/phosphate buffer in water, 0.1% from 5/95 to 70/30) at a flow rate of 0.7 mL$\cdot$min$^{-1}$. After complete consumption of **10** within 10 min, the reaction mixture was filtered through a Celite pad and concentrated under reduced pressure. The crude was purified on silica gel (hexane/EtOAc from 82:18 to 76:24 + 0.1 vol% of formic acid) to obtain Loxoprofen **5** as a colourless oil in 71% yield (87 mg). The analytical data are in agreement with the literature.[6]

**Rf** = 0.43 (hexane/ethyl acetate 6:4 + 0.25 vol% of formic acid). **$^1$H NMR** (400 MHz, $CDCl_3$) δ 7.23 (d, *J* = 8.1 Hz, 2H), 7.12 (d, *J* = 8.1 Hz, 2H), 3.71 (q, *J* = 7.2 Hz, 1H), 3.12 (dd, *J* = 13.9, 4.1 Hz, 1H), 2.50 (dd, *J* = 13.9, 9.5 Hz, 1H), 2.41 – 2.24 (m, 2H), 2.16 – 2.03 (m, 2H), 2.02 – 1.87 (m, 1H), 1.84 – 1.62 (m, 1H), 1.61 – 1.42 (m, 4H). **$^{13}$C NMR** (101 MHz, $CDCl_3$) δ 220.5, 180.1, 139.3, 137.8, 129.34 (2C), 127.78 (2C), 51.1, 45.0, 38.3, 35.3, 29.4, 20.7, 18.3.

## 8.3 Comparison between batch and flow approaches

The performances of the experiment under continuous flow conditions were evaluated in terms of productivity and space-time yield. The productivity is defined as the amount of product per hour provided by the system, while the space-time yield (STY) as the amount of product per hour, per unit of volume (see section 4.5). The productivity and the space-time yield values for the hydrogenation reaction to Loxoprofen **5** were calculated considering the same reaction conditions for both batch and continuous flow protocols (0.5 mmol of compound **10** in methanol at a concentration of 0.05 M, rt, 1 atm) as illustrated in **Table S10**.

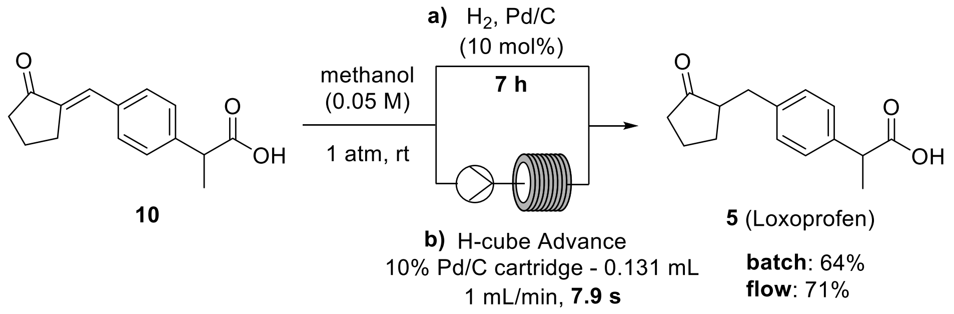


| ENTRY | YIELD (%) | TIME (min) | PRODUCTIVITY (mg · h$^{-1}$) | PRODUCTIVITY RELATIVE FACTOR | STY (g · h$^{-1}$ · L$^{-1}$) | STY RELATIVE FACTOR |
|---|---|---|---|---|---|---|
| **BATCH** | 64 | 420 | 11 | 1 | 1.1 | 1 |
| **FLOW** | 71 | 10 | 525 | **48** | 4008 | **3644** |

**Table S10:** Comparison between batch and flow conditions for the reduction of compound **10** to obtain Loxoprofen **5**.

# 9. Synthesis of compound 12 via photo-Favorskii rearrangement

## 9.1. General procedure for the synthesis of 2-(9-acetyl-6-chloro-9H-carbazol-2-yl)propanoic acid (12) via a photo-Favorskii rearrangement in batch (GP3)

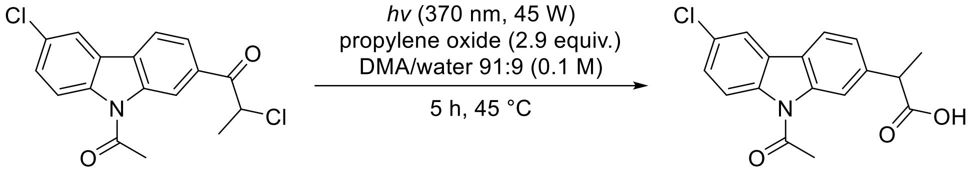


**11** **12**

A 10 mL glass vial was charged with 1-(9-acetyl-6-chloro-9H-carbazol-2-yl)-2-chloropropan-1-one **11** (133.7 mg, 0.4 mmol, 0.1 M), the desired scavenger (typically, propylene oxide, 80 µL, 1.14 mmol, 2.9 equiv.) and dissolved in the desired solvent (typically a 4 mL 91:9 solvent mixture of DMA/water). The resulting reaction mixture was irradiated using the desired light source for the desired time at the desired temperature (typically, a 370 nm Kessil lamp was used at 45 °C for 5 h). The reaction mixture was then concentrated under reduced pressure and the resulting residue was chromatographed on silica gel (hexane/ethyl acetate from 75:25 to 72:28 + 0.25 vol% of formic acid) to obtain compound **12** in 23% yield as a yellowish solid (29 mg). Alternatively, the crude can be dissolved in DCM (4 mL) and extracted with a solution of $NaHCO_3$ at a concentration of 0.125M (2 × 4 mL). The aqueous layers can be collected, acidified with HCl 1M until pH~2 and extracted with DCM (2 × 8 mL), dried over anhydrous sodium sulfate and concentrated under reduced pressure to afford the desired product as a white solid.

**Rf** = 0.21 (hexane/ethyl acetate 7:3 + 0.25 vol% of formic acid). **$^{1}$H NMR** (300 MHz, CDCl3) δ 8.17 (d, J = 4.9 Hz, 1H), 8.13 (s, 1H), 7.95 – 7.84 (m, 2H), 7.41 (dd, J = 9.0, 2.2 Hz, 2H), 3.95 (q, J = 7.1 Hz, 1H), 2.87 (s, 3H), 1.63 (d, J = 7.1 Hz, 3H). **$^{13}$C NMR** (75 MHz, CDCl3) δ 178.6, 169.9, 140.3, 139.5, 137.4, 129.6, 127.7, 127.5, 124.9, 123.6, 120.4, 119.8, 117.5, 115.8, 45.9, 27.8, 18.8. **HRMS (ESI-TOF)** *m/z* calcd for $C_{17}H_{14}ClNO_3Na^+$: 338.0554 $[M + Na]^+$; found: 338.0560, mass error +1.77 ppm.

### 9.1.1 SOLVENT SCREENING

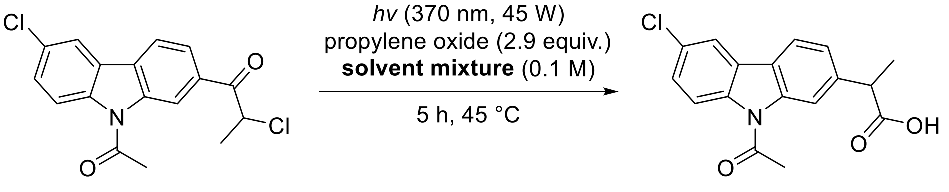


**11** **12**

| ENTRY | SOLVENT MIXTURE | YIELD (%) |
| --- | --- | --- |
| **1** | acetone/water 9:1 | traces |
| **2** | DMA/water 91:9 | **23** |
| **3** | DMF/ water 91:9 | 16 |

**Table S11**: Solvent mixture screening for the photo-Favorskii rearrangement on Carprofen derivative **11.**

## 9.1.2 SCAVENGER SCREENING

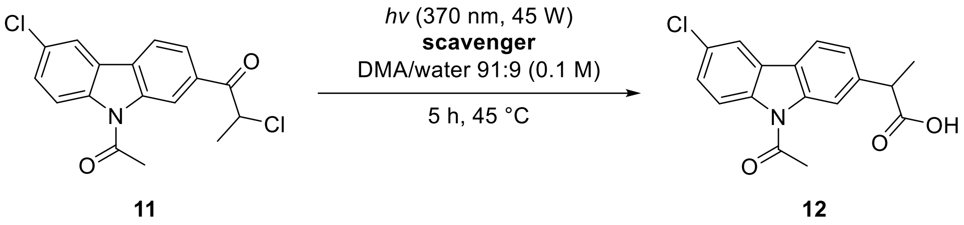


| ENTRY | SCAVENGER | YIELD (%) |
|---|---|---|
| **1** | propylene oxide (2.9 equiv.) | **23** |
| **2** | HCOONa (1.6 equiv.) | 12 |
| **3** | epichlorohydrin (2.5 equiv.) | 17 |
| **4** | - | < 10 |

**Table S12**: Scavenger screening for the photo-Favorskii rearrangement on Carprofen derivative **11.**

## 9.1.3 LIGHT SOURCE AND TIME SCREENING

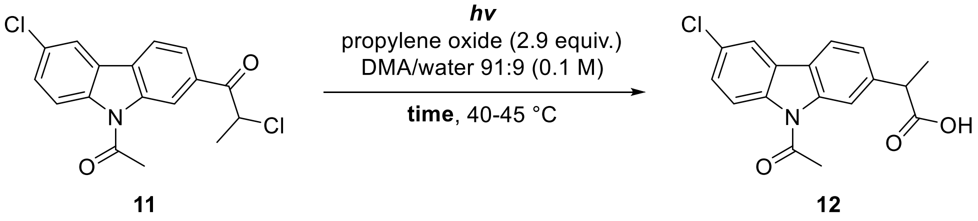


| ENTRY | LIGHT SOURCE (nm) | TIME (h) | YIELD (%) |
|---|---|---|---|
| **1** | 254 | 5 | 9 |
| **2** | 370 | 2.5 | 10 |
| **3** | 370 | 5 | **23** |
| **4** | 370 | 16 | **24** |
| **5** | 390 | 5 | **22** |
| **6** | 390 | 16 | 18 |
| **7*** | 390 | 5 | 8 |

* reaction performed without adding 2.9 equiv. of propylene oxide as scavenger.

**Table S13**: Light source (lamp) and reaction time screening for the photo-Favorskii rearrangement on Carprofen derivative **11.**

## 9.2. General procedure for the synthesis of 2-(9-acetyl-6-chloro-9H-carbazol-2-yl)propanoic acid (**12**) via photo-Favorskii rearrangement under flow conditions (GP4)

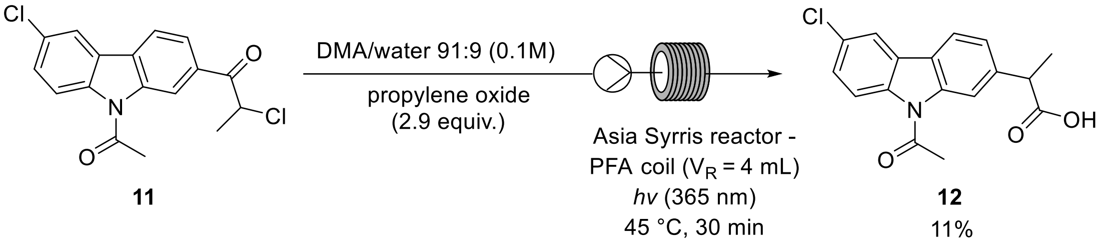


A 10 mL glass vial was charged with 1-(9-acetyl-6-chloro-9H-carbazol-2-yl)-2-chloropropan-1-one **11** (133.7 mg, 0.4 mmol, 0.1 M), propylene oxide (80 µL, 1.14 mmol, 2.9 equiv.) and dissolved in a solvent mixture DMA/water (91:9). The resulting reaction mixture was irradiated with 365 nm LEDs using the Asia Syrris System at 45 °C. The parameters are the following: 0.5 mm for the internal diameter, a reactor volume of 4 mL, 30 minutes as residence time and 133 µL · min$^{-1}$ was the set flow rate. After the irradiation, the reaction mixture was concentrated under reduced pressure and the resulting residue was chromatographed on silica gel (hexane/ethyl acetate from 75:25 to 72:28 + 0.25 vol% of formic acid) to obtain compound **12** in 11% yield as a yellowish solid (14 mg). Alternatively, the crude can be dissolved in DCM (4 mL) and extracted with a solution of $NaHCO_3$ at a concentration of 0.125M (2 × 4 mL). The aqueous layers can be collected, acidified with HCl 1M until pH~2 and extracted with DCM (2 × 8 mL), dried over anhydrous sodium sulfate and concentrated under reduced pressure to afford the desired product.

**Rf** = 0.21 (hexane/ethyl acetate 7:3 + 0.25 vol% of formic acid). **$^1$H NMR** (300 MHz, CDCl3) δ 8.17 (d, J = 4.9 Hz, 1H), 8.13 (s, 1H), 7.95 – 7.84 (m, 2H), 7.41 (dd, J = 9.0, 2.2 Hz, 2H), 3.95 (q, J = 7.1 Hz, 1H), 2.87 (s, 3H), 1.63 (d, J = 7.1 Hz, 3H). **$^{13}$C NMR** (75 MHz, CDCl3) δ 178.6, 169.9, 140.3, 139.5, 137.4, 129.6, 127.7, 127.5, 124.9, 123.6, 120.4, 119.8, 117.5, 115.8, 45.9, 27.8, 18.8. **HRMS (ESI-TOF)** *m/z* calcd for $C_{17}H_{14}ClNO_3Na^+$: 338.0554 [M + Na]$^+$; found: 338.0560, mass error +1.77 ppm.

## 9.3. Control experiments

Two control experiments were conducted in the absence of light at 25 and 60 °C, recovering only compound **11** after 16 h and confirming that UV irradiation is required for the photochemical rearrangement.

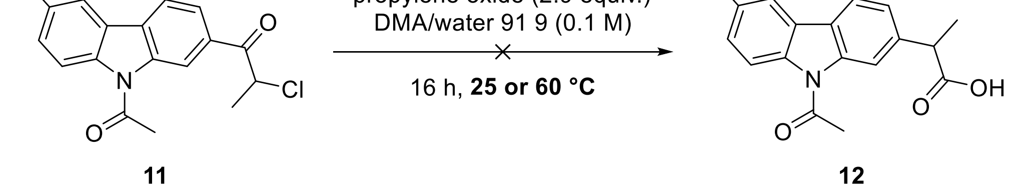

## 9.4. Comparison between batch and flow approaches

The performances of the experiment under continuous flow conditions were evaluated in terms of productivity and space-time yield (STY). The productivity is defined as the amount of product per hour provided by the system, while STY as the amount of product per hour, per unit of volume (see section 4.5). Productivity and STY values for the photo-Favorskii rearrangement to obtain aryl propionic acid **12** were calculated considering the same reaction conditions for both batch and continuous flow protocols (0.4 mmol of **11** in a 91:9 mixture of DMA/water at a concentration of 0.1 M, at 45 °C, under UVA irradiation) as illustrated in **Table S14**.

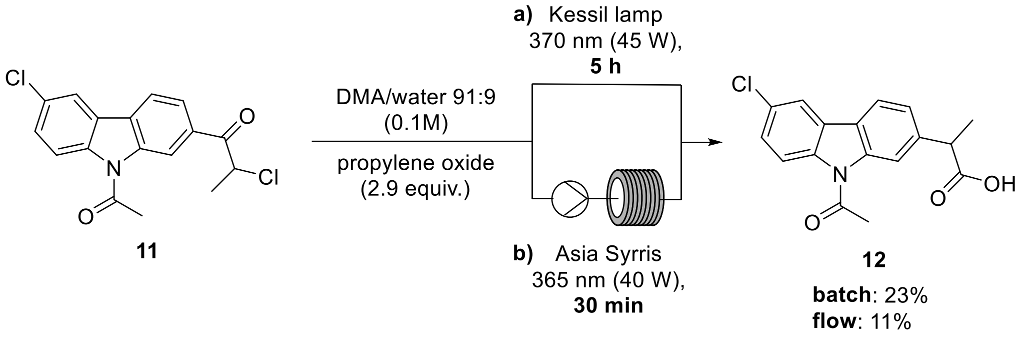


| | YIELD (%)* | TIME (h) | PRODUCTIVITY (mg·h$^{-1}$) | PRODUCTIVITY RELATIVE FACTOR | STY (g · h$^{-1}$ · L$^{-1}$) | STY RELATIVE FACTOR |
|---|---|---|---|---|---|---|
| **BATCH** | 23 | 5 | 6 | 1 | 1.5 | 1 |
| **FLOW** | 11 | 0.5 | 28 | **5** | 7 | **5** |

**Table S14:** Comparison between batch and flow conditions for the photo-Favorskii rearrangement on compound **11.**

# 10. Synthesis of Carprofen 3

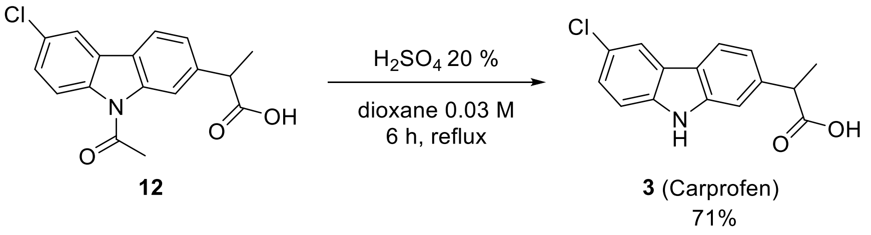


According to a modified literature procedure,[9] to a stirring solution of 2-(9-acetyl-6-chloro-9H-carbazol-2-yl)propanoic acid **12** (31.6 mg, 0.1 mmol, 1 equiv., 0.03 M in dioxane) in dioxane (3 mL), 1 mL of 20% sulfuric acid was added. The resulting mixture was heated under reflux for 6 h, until complete consumption of **12**. After cooling to rt, the reaction mixture was concentrated under reduced pressure. The crude was chromatographed on silica gel (hexane/ethyl acetate from 76:24 to 74:26, adding 0.25 vol% of formic acid). Carprofen **3** was isolated in 71% yield as a white solid (19.5 mg). All the analytical data are in accordance with the literature.[10]

**Rf** = 0.3 (hexane/ethyl acetate 65:35 + 0.25 vol% of formic acid). **$^1$H NMR** (400 MHz, $CDCl_3$) δ 8.03 (br s, 1H), 7.99 (d, $J$ = 1.8 Hz, 1H), 7.96 (d, $J$ = 8.1 Hz, 1H), 7.39 (s, 1H), 7.38 – 7.30 (m, 2H), 7.21 (dd, $J$ = 8.1, 1.5 Hz, 1H), 3.92 (q, $J$ = 7.1 Hz, 1H), 1.60 (d, $J$ = 7.1 Hz, 3H). **$^{13}$C NMR** (101 MHz, $CDCl_3$) δ 178.8, 140.4, 138.7, 138.2, 126.1, 125.2, 124.4, 122.0, 120.9, 120.2, 119.9, 111.7, 109.89, 45.6, 18.7.

## 11. NMR spectra

**6-Chloro-α-methyl-9*H*-carbazole-2-acetic acid 3 (Carprofen)** **$^{1}$H-NMR** (400 MHz; $CDCl_3$)

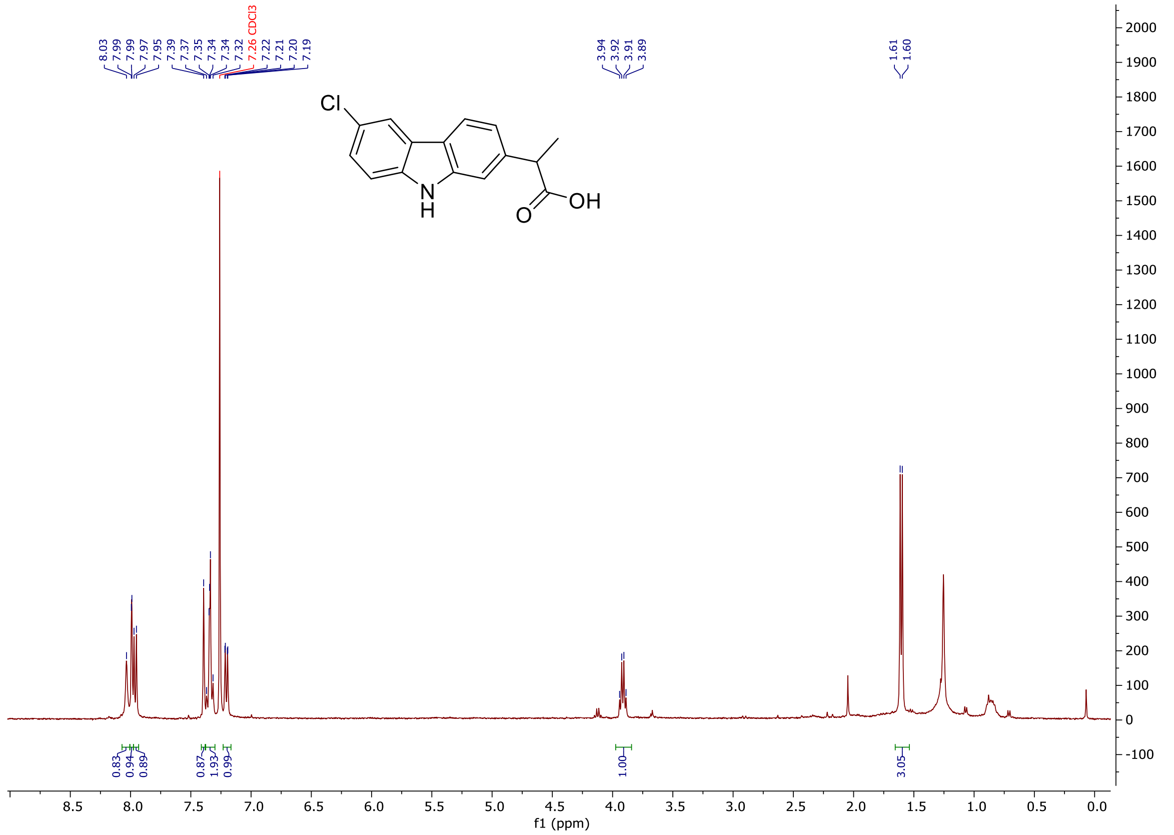

**$^{13}$C-NMR** (101 MHz; $CDCl_3$)

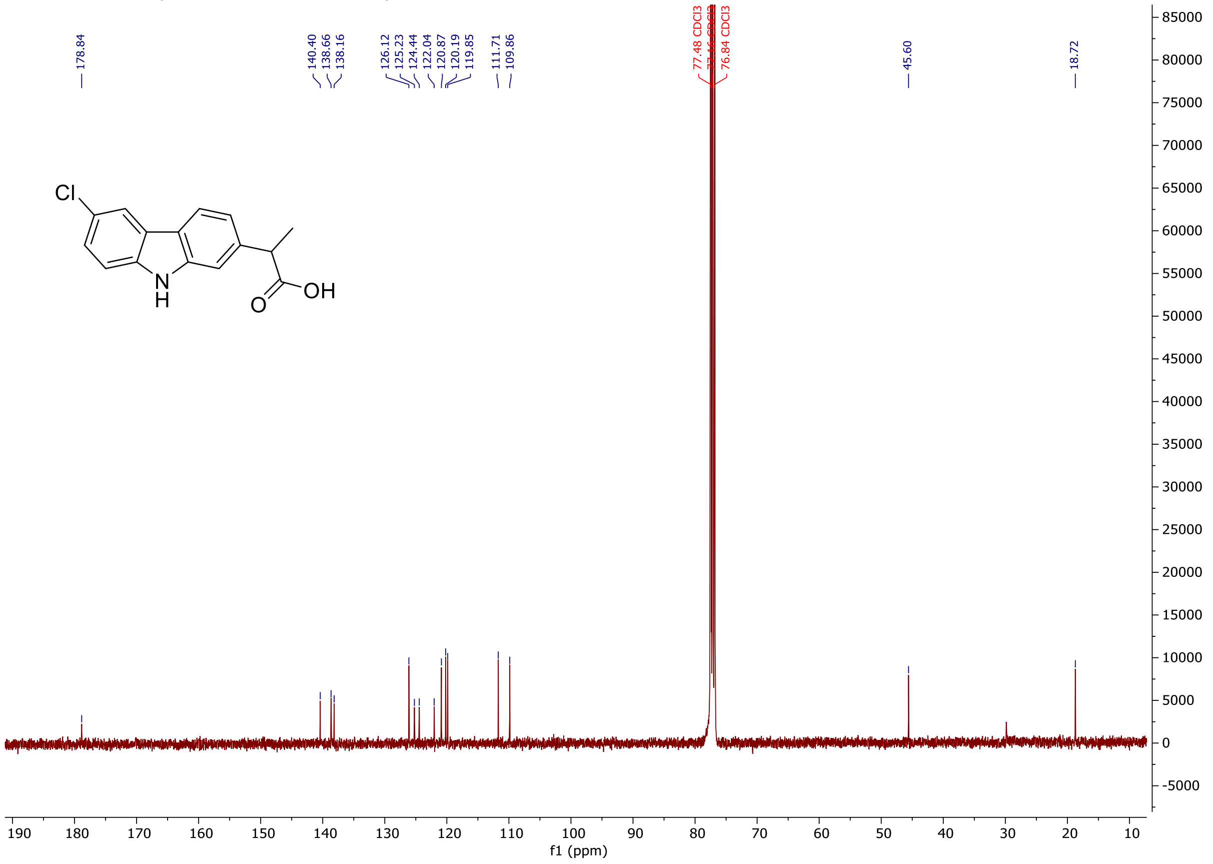

## 2-(4-((2-oxocyclopentyl)methyl)phenyl)propanoic acid 5 (Loxoprofen)

**$^1$H-NMR** (300 MHz; $CDCl_3$)

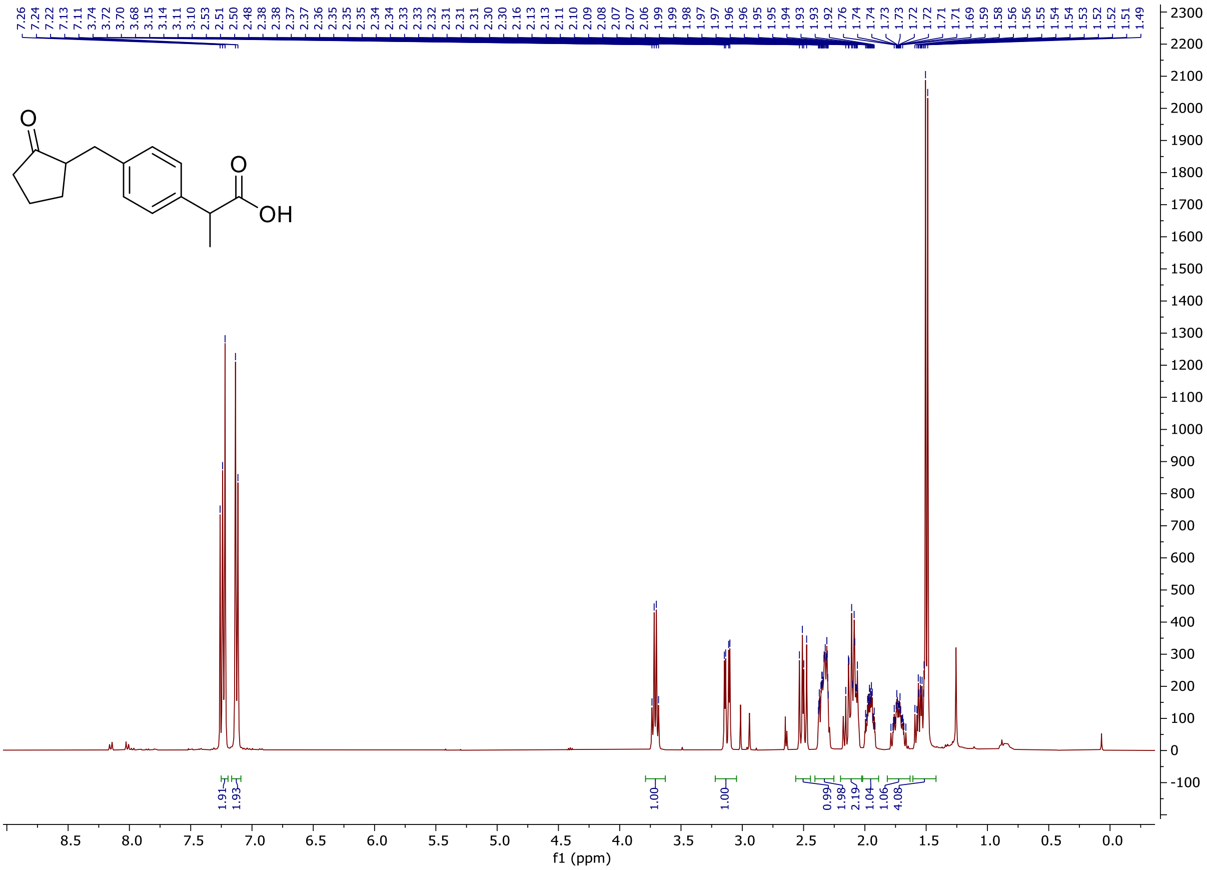


**$^{13}$C-NMR** (75 MHz; $CDCl_3$)

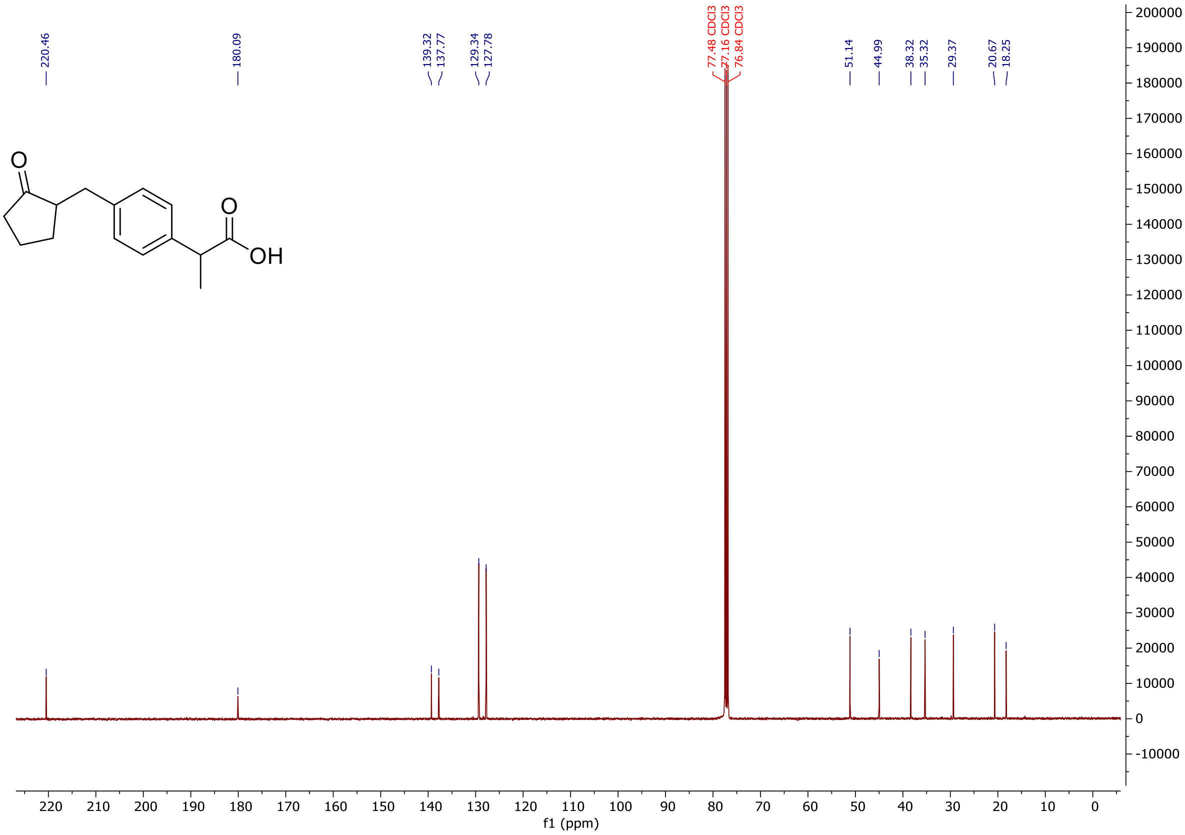

**2-chloro-1-(*p*-tolyl)propan-1-one (6)** **$^1$H-NMR** (300 MHz; $CDCl_3$)

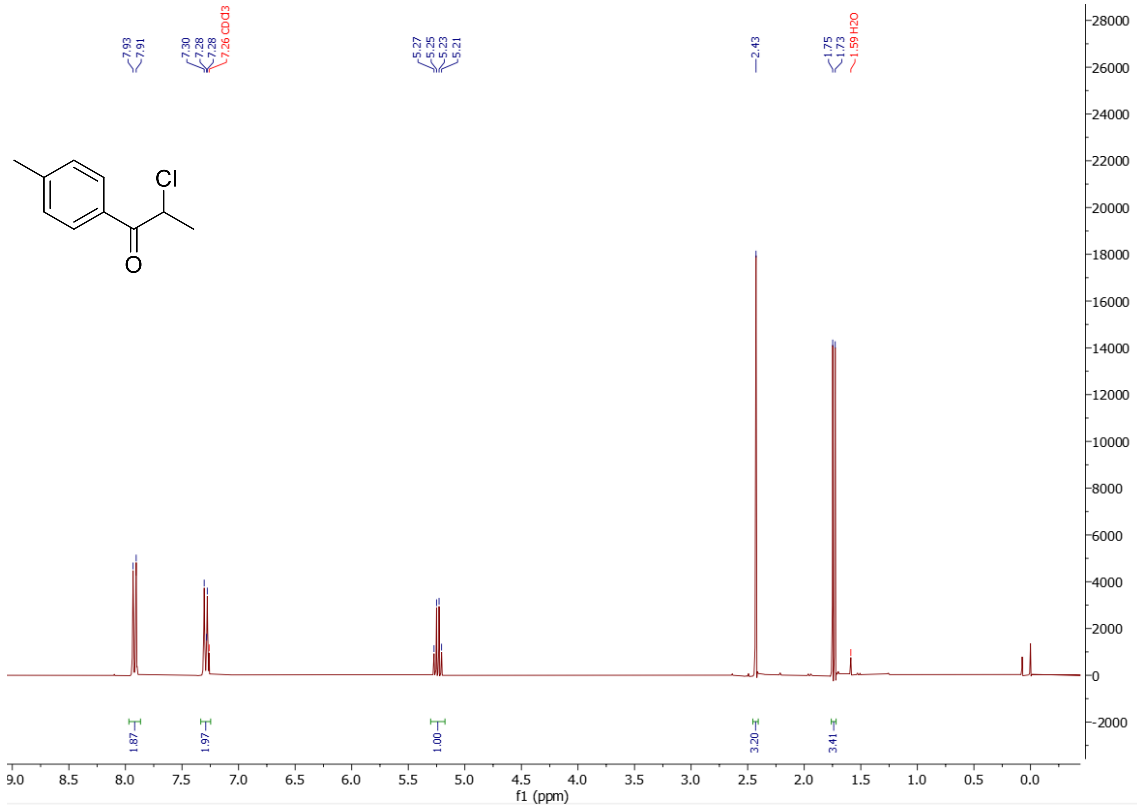


**2-(*p*-tolyl)propanoic acid (7)** **$^1$H-NMR** (300 MHz; $CDCl_3$)

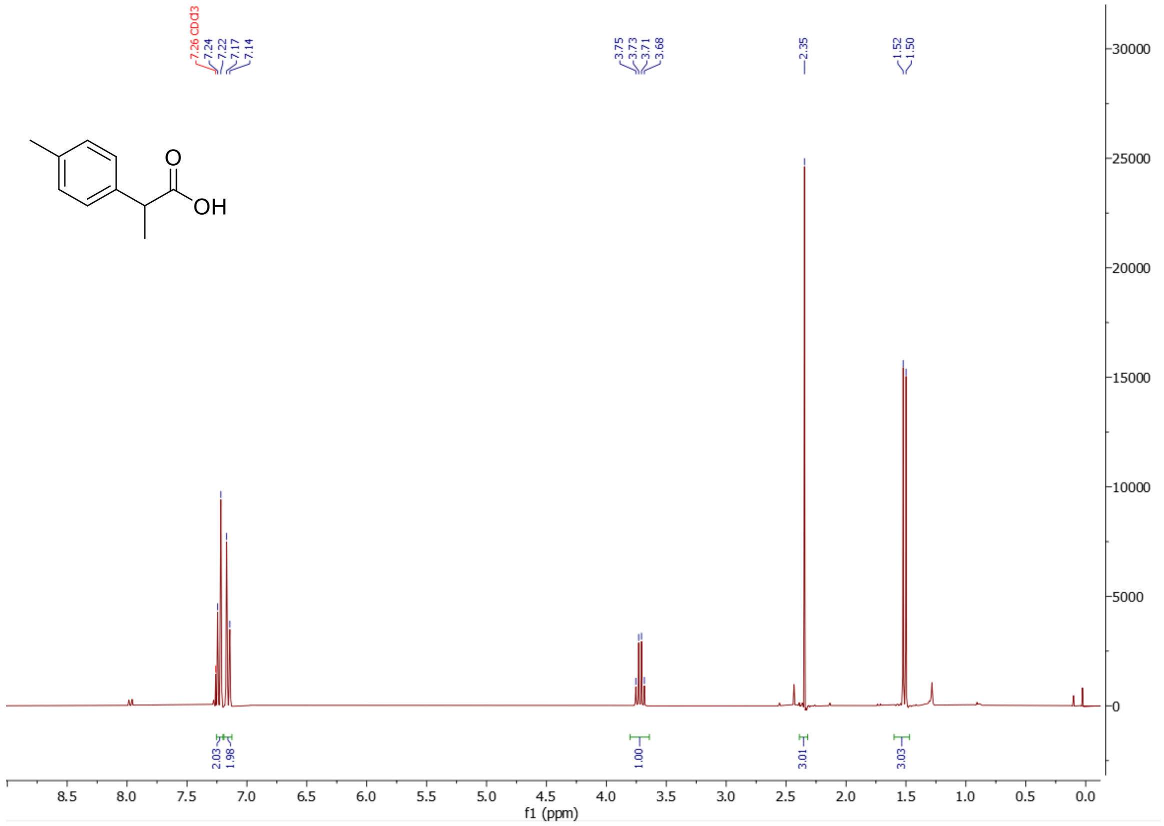

**$^{13}$C-NMR** (75 MHz; $CDCl_3$)

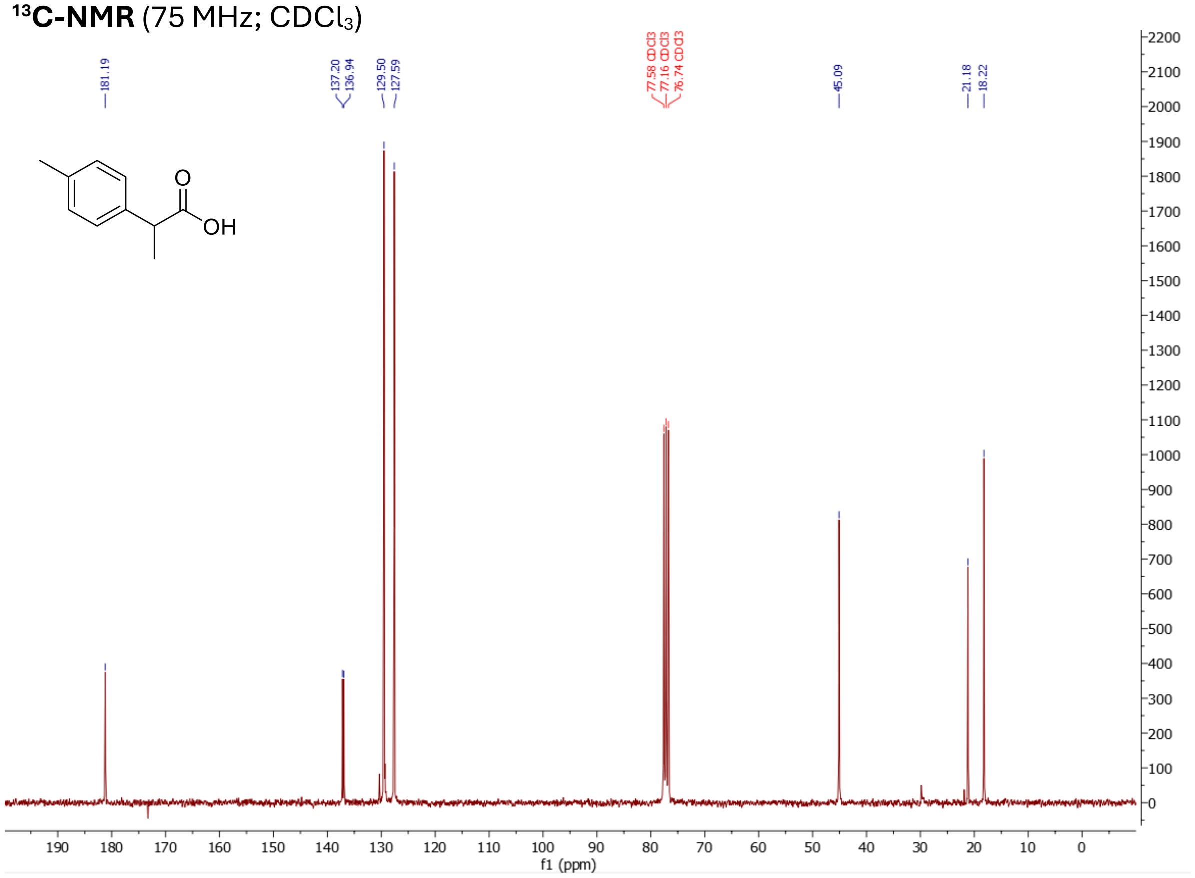


## 2-(4-formylphenyl)propanoic acid (8)

**$^{1}$H-NMR** (300 MHz; $CDCl_3$)

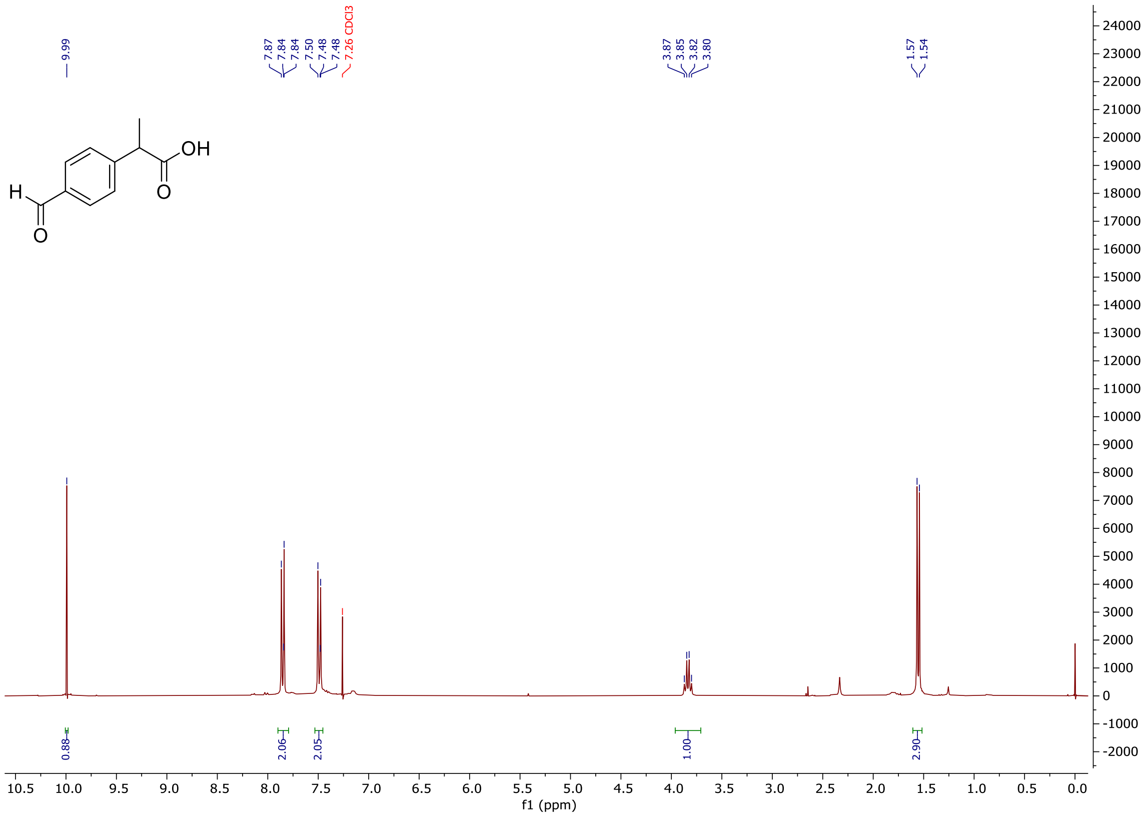

**$^{13}$C-NMR** (75 MHz; $CDCl_3$)

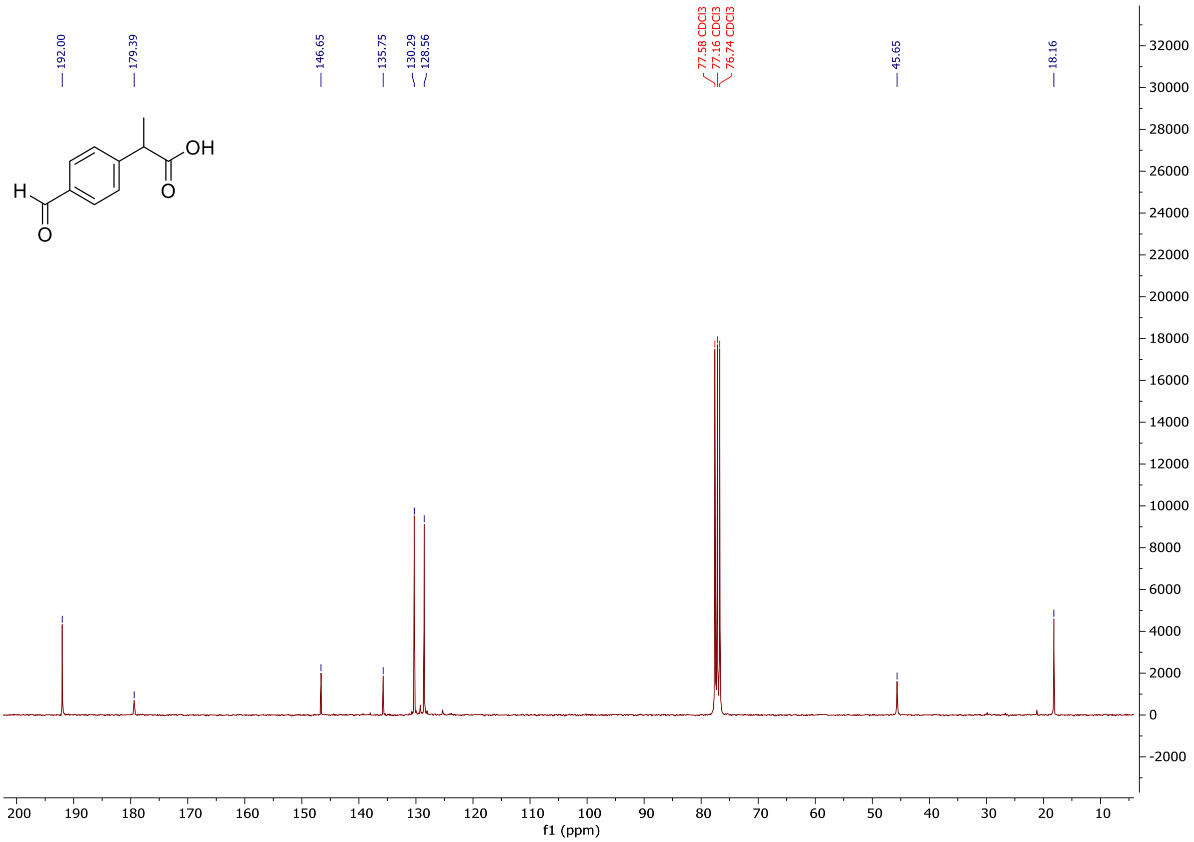


**COSY** ($CDCl_3$)

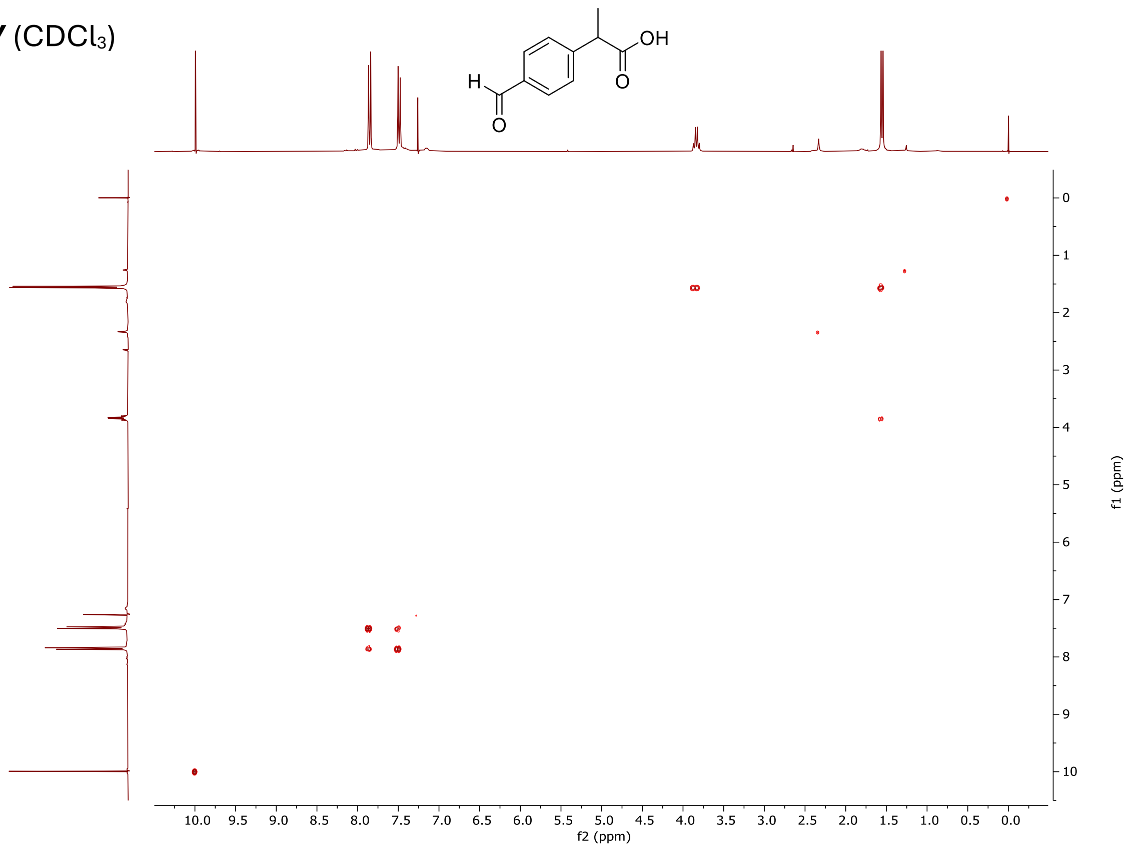

**HSQC** ($CDCl_3$)

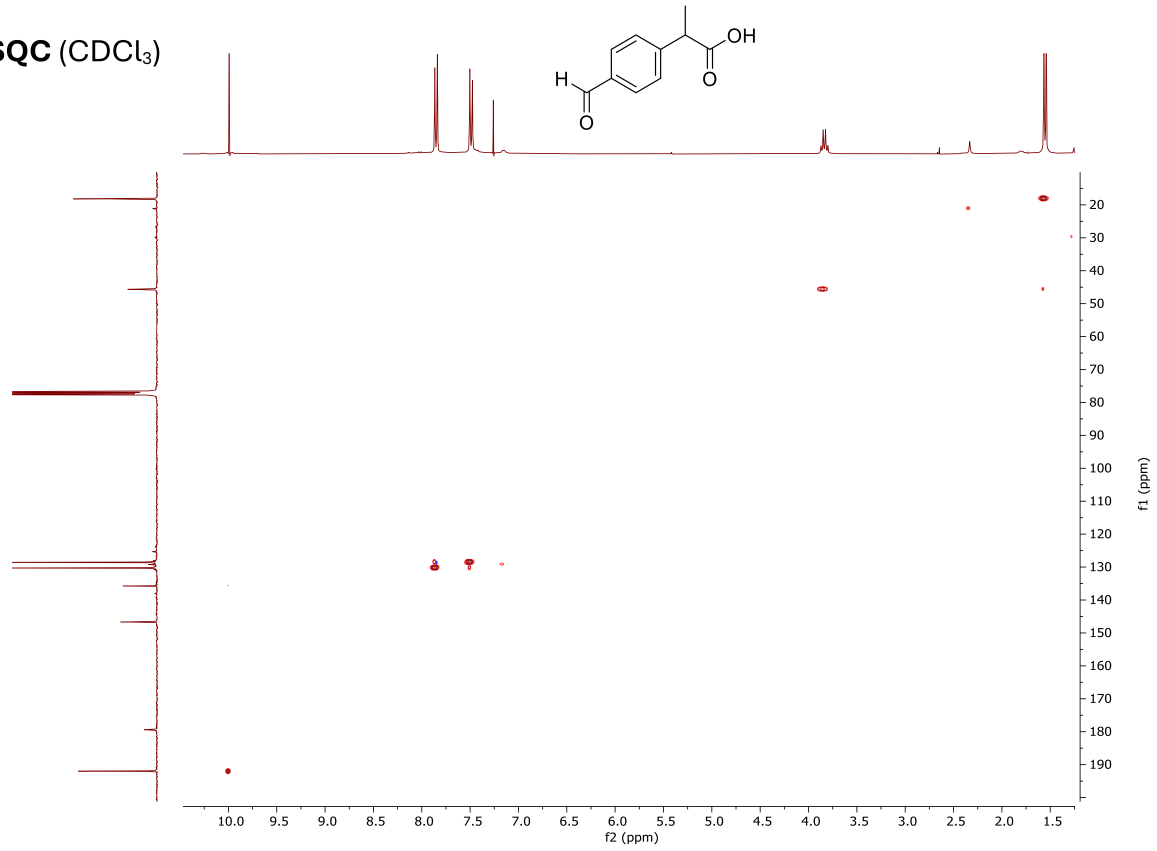


**HMBC** ($CDCl_3$)

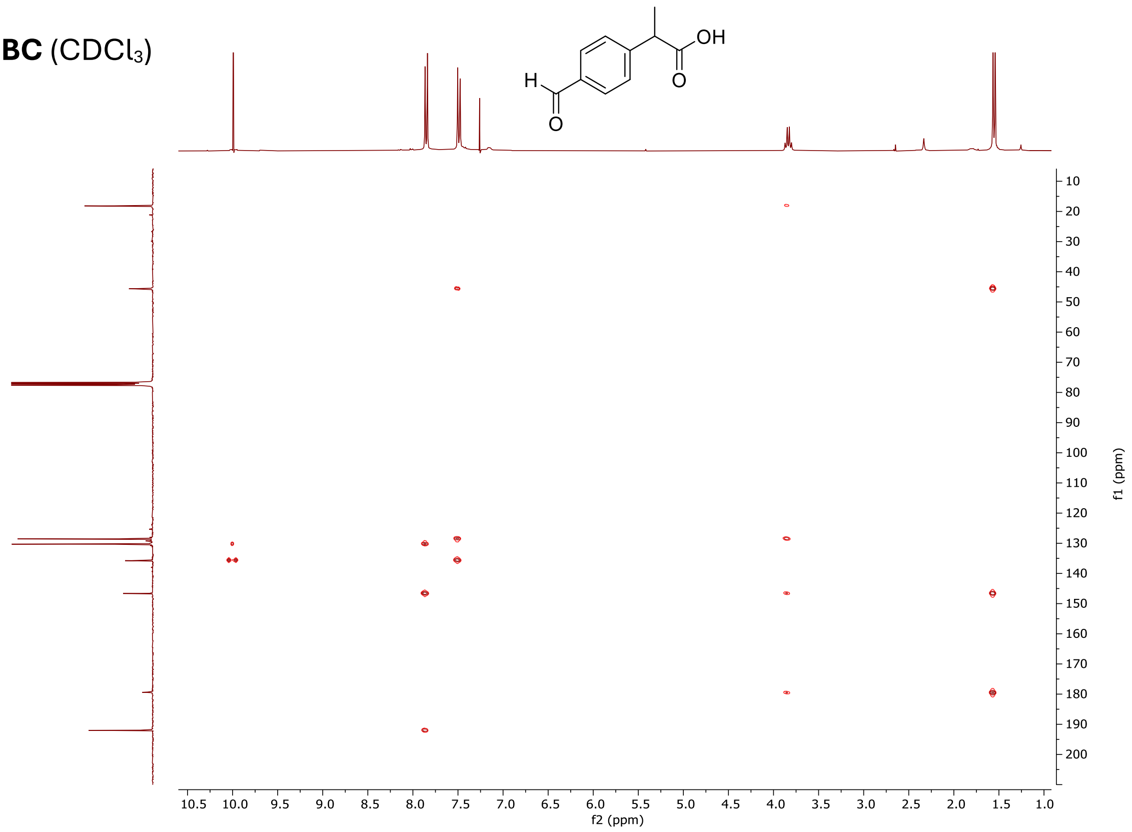

**(*E*)-2-(4-((2-oxocyclohexylidene)methyl)phenyl)propanoic acid 9 (Pelubiprofen)**

**$^1$H-NMR** (300 MHz; $CDCl_3$)

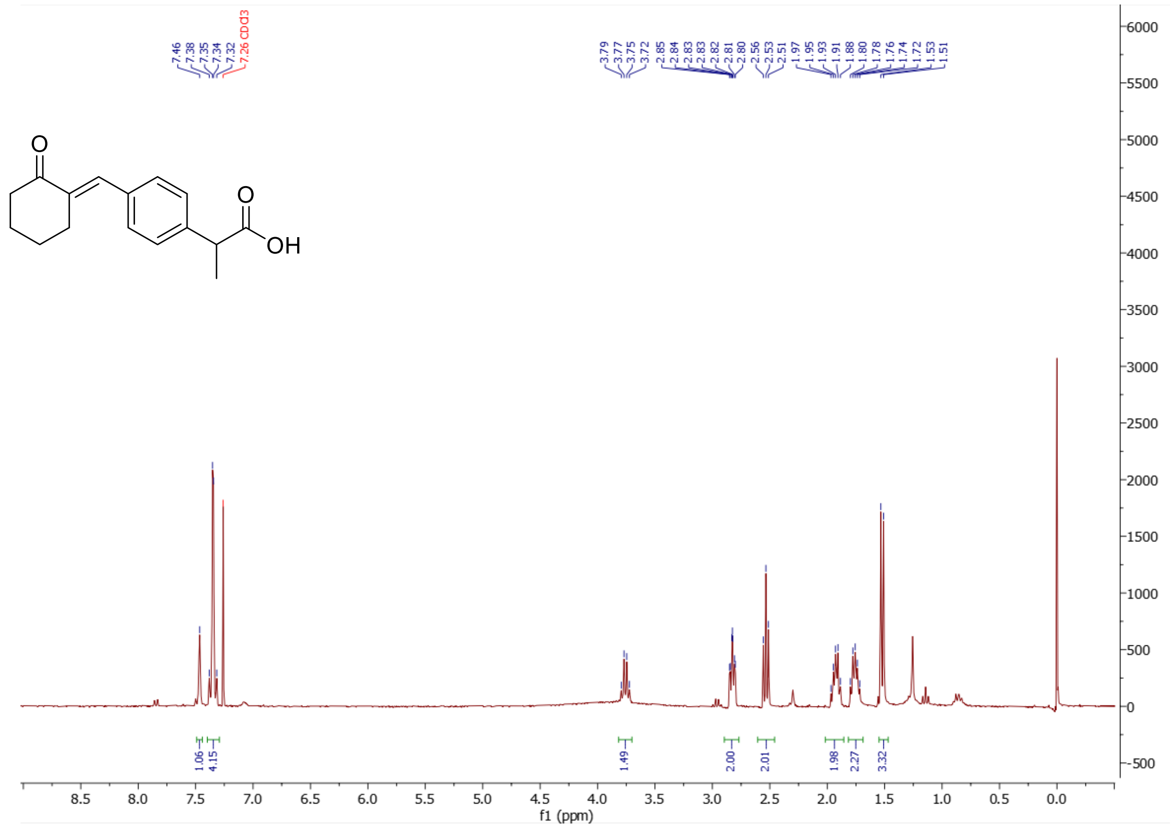

**$^{13}$C-NMR** (75 MHz; $CDCl_3$)

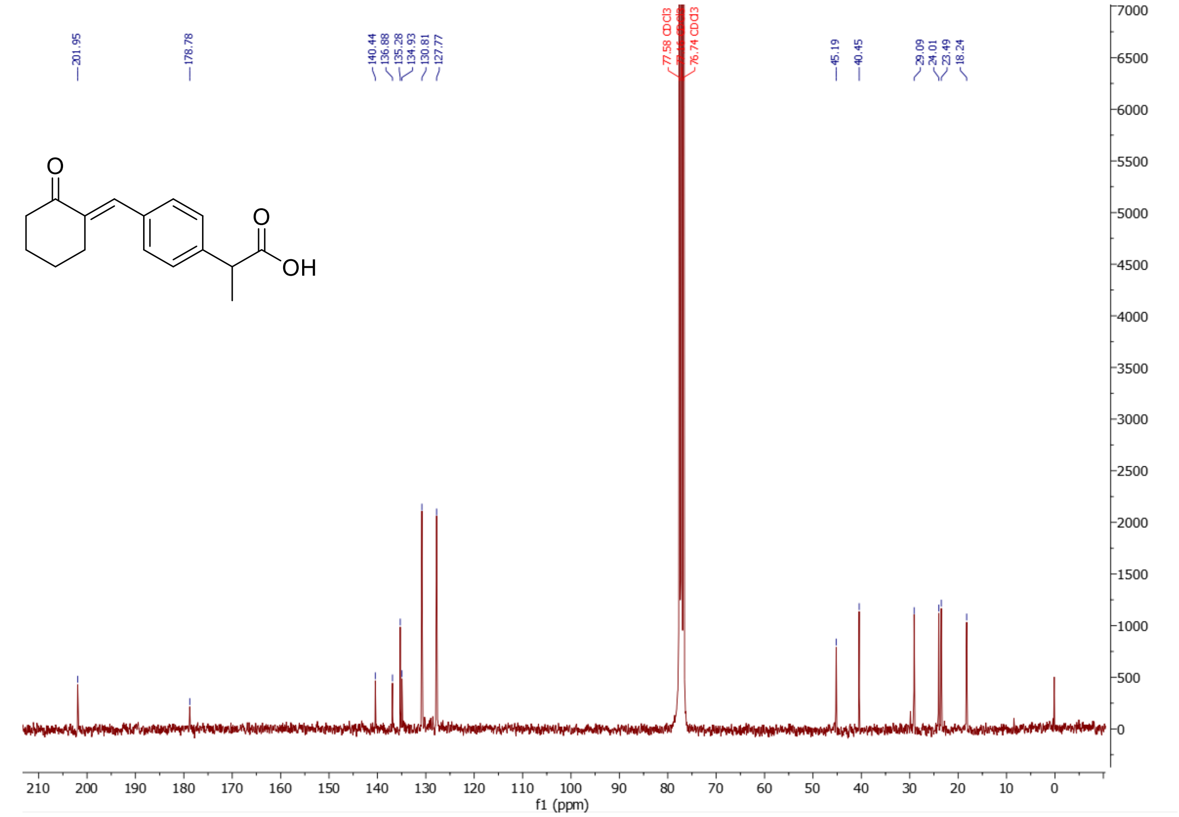

**(*E*)-2-(4-((2-oxocyclopentylidene)methyl)phenyl)propanoic acid (10)** **$^{1}$H-NMR** (300 MHz; $CDCl_3$)

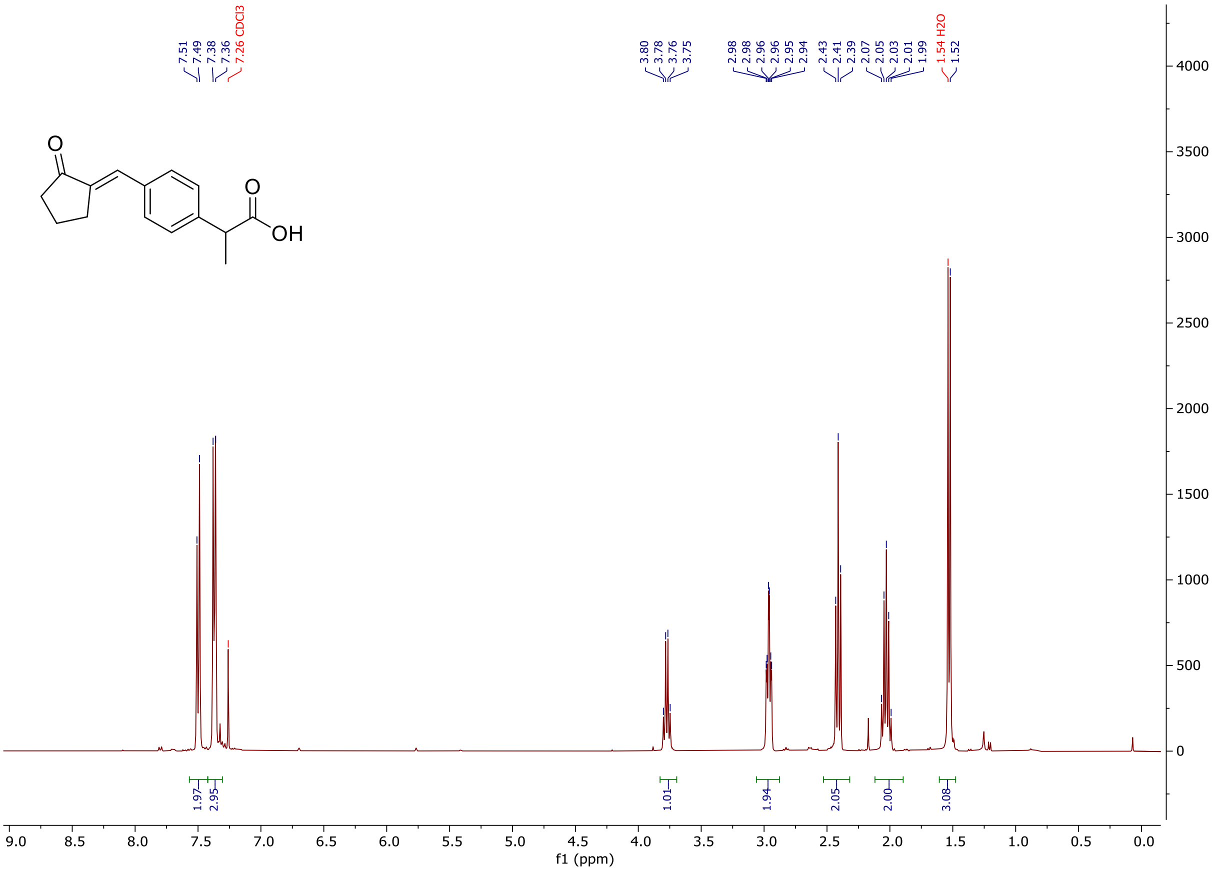


**$^{13}$C-NMR** (75 MHz; $CDCl_3$)

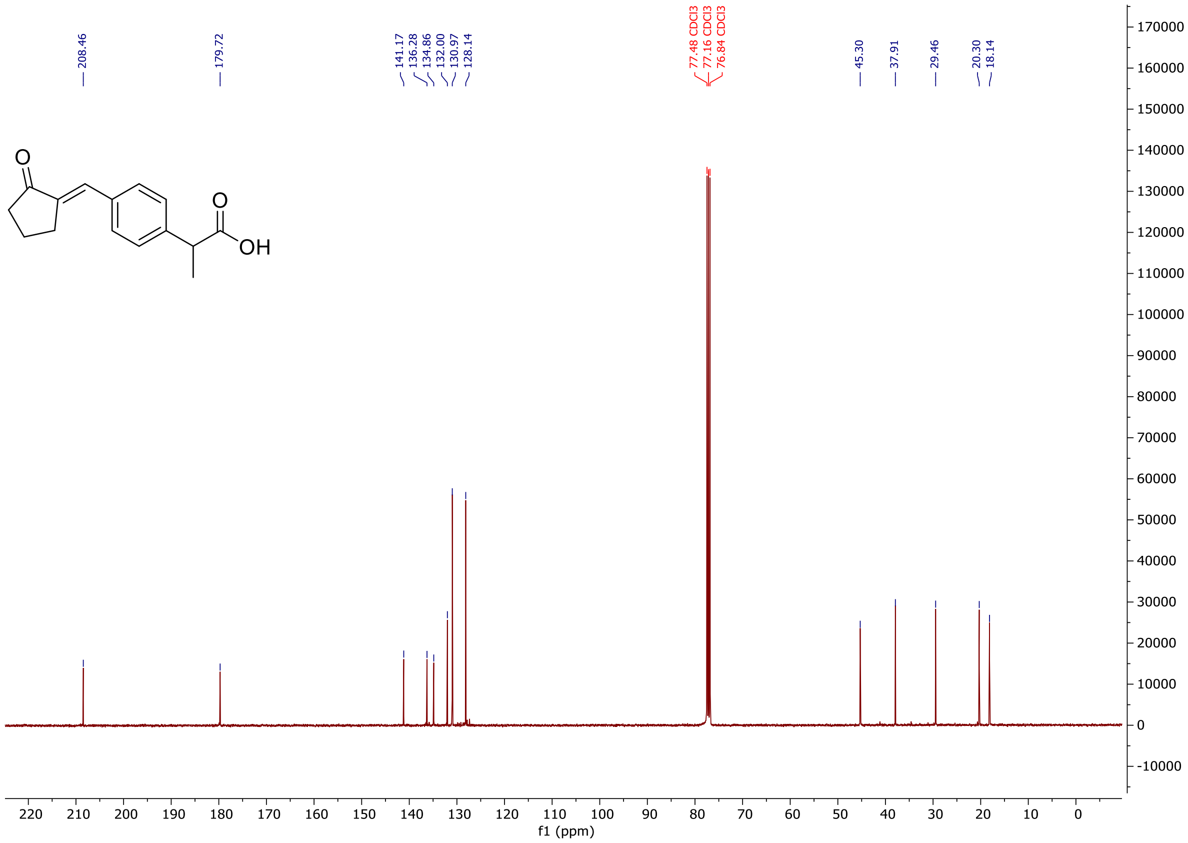

**2-(9-acetyl-6-chloro-9H-carbazol-2-yl)propanoic acid (12)**

**$^{1}$H-NMR** (300 MHz; $CDCl_3$)

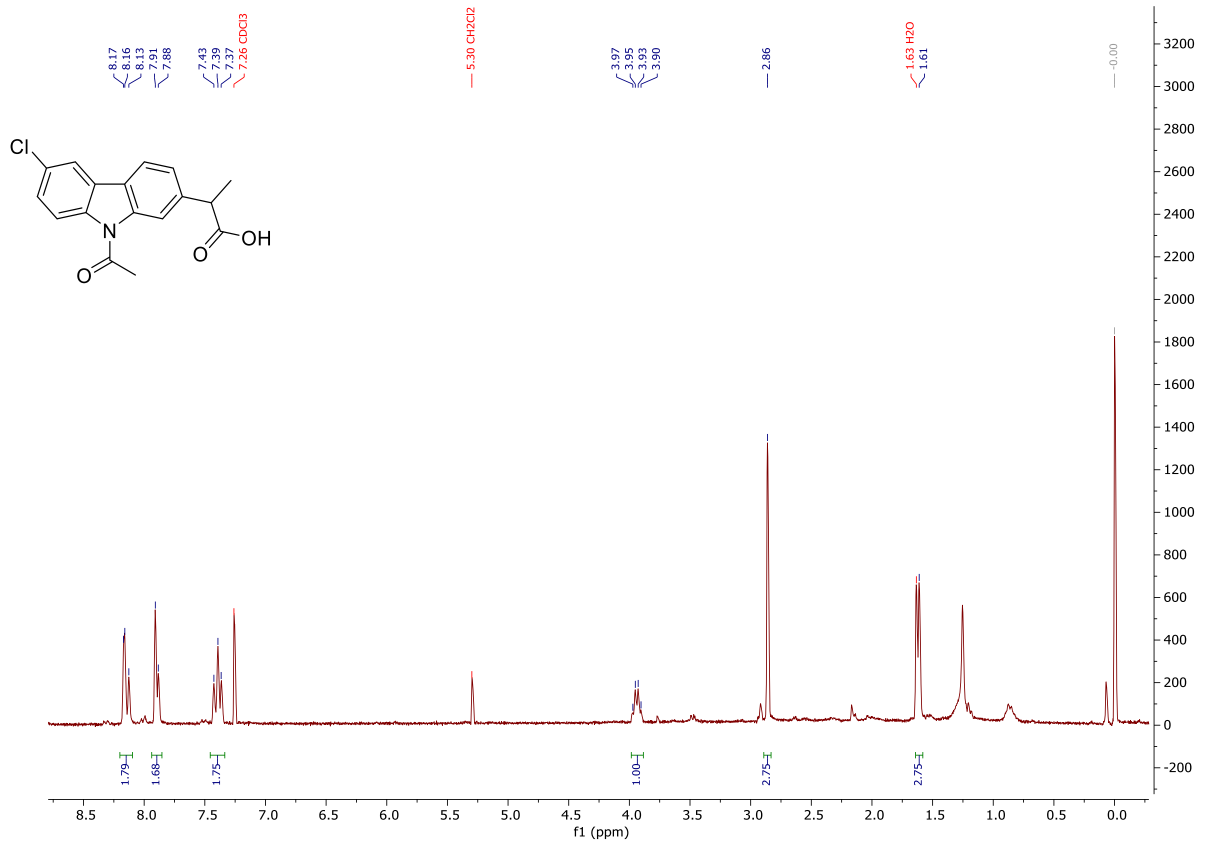


**$^{13}$C-NMR** (75 MHz; $CDCl_3$)

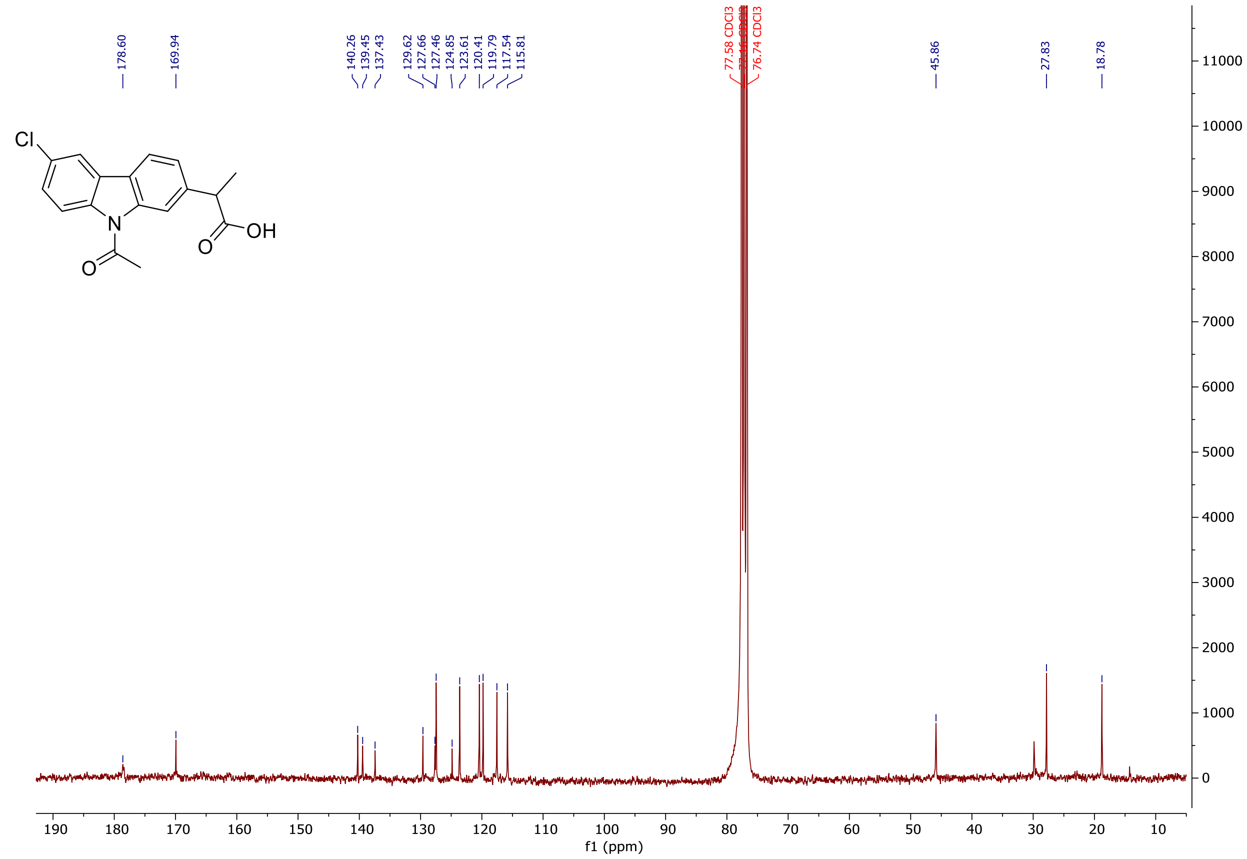

**COSY** ($CDCl_3$)

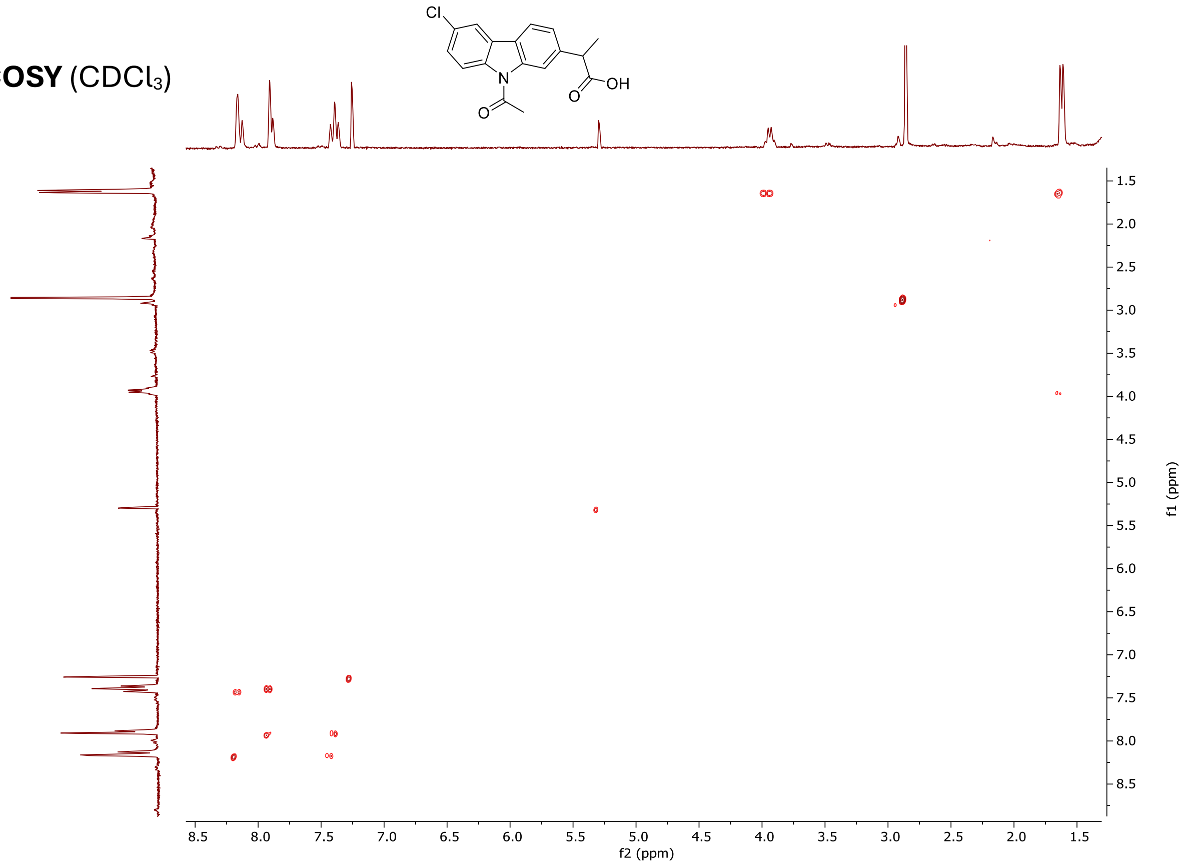


**HSQC** ($CDCl_3$)

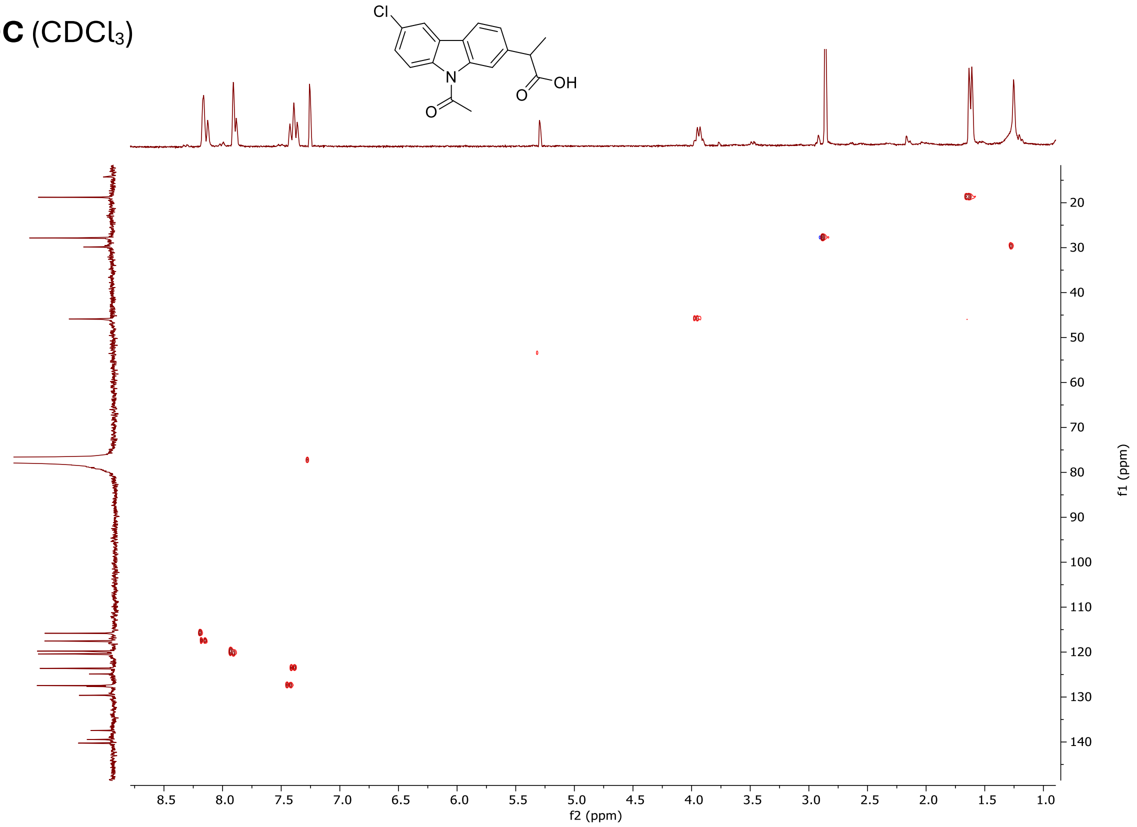

**HMBC** ($CDCl_3$)

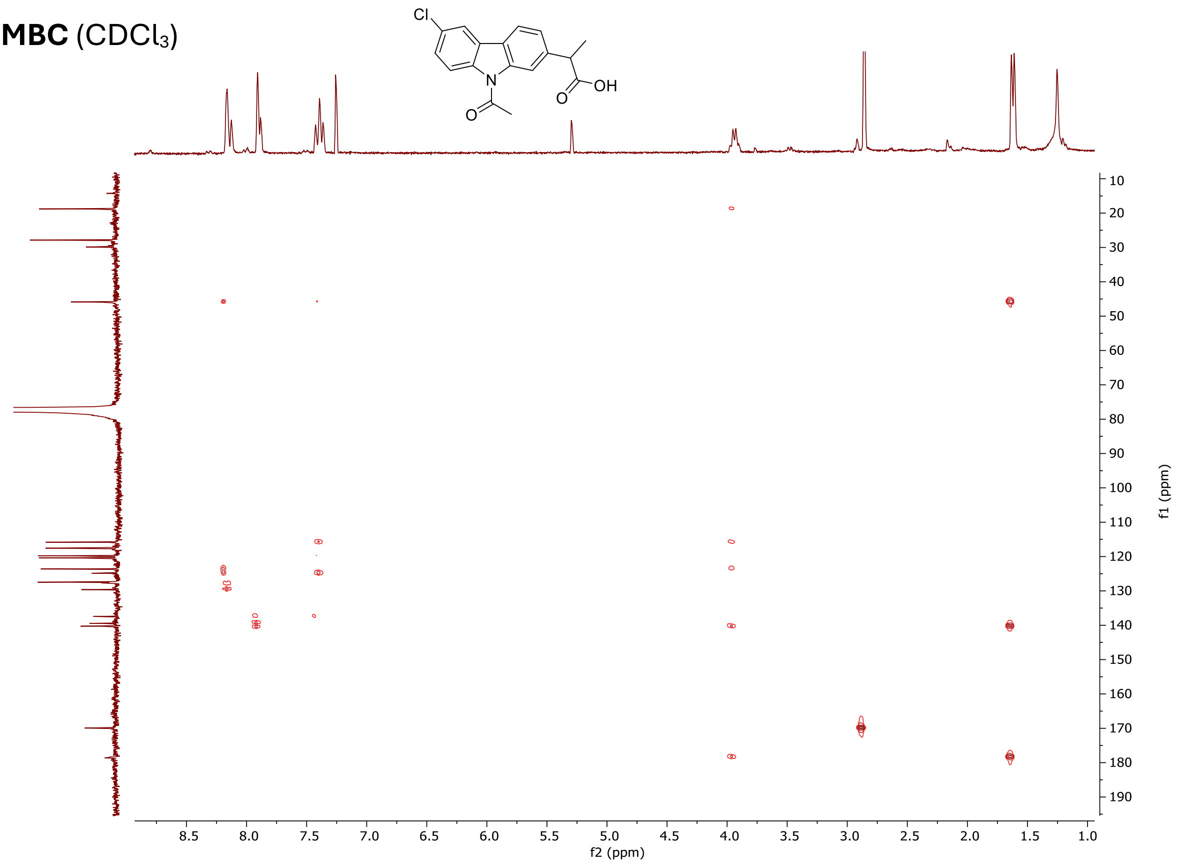


**(*E*)-2-benzylidenecyclohexan-1-one (A)**

**$^1$H-NMR** (300 MHz; $CDCl_3$)

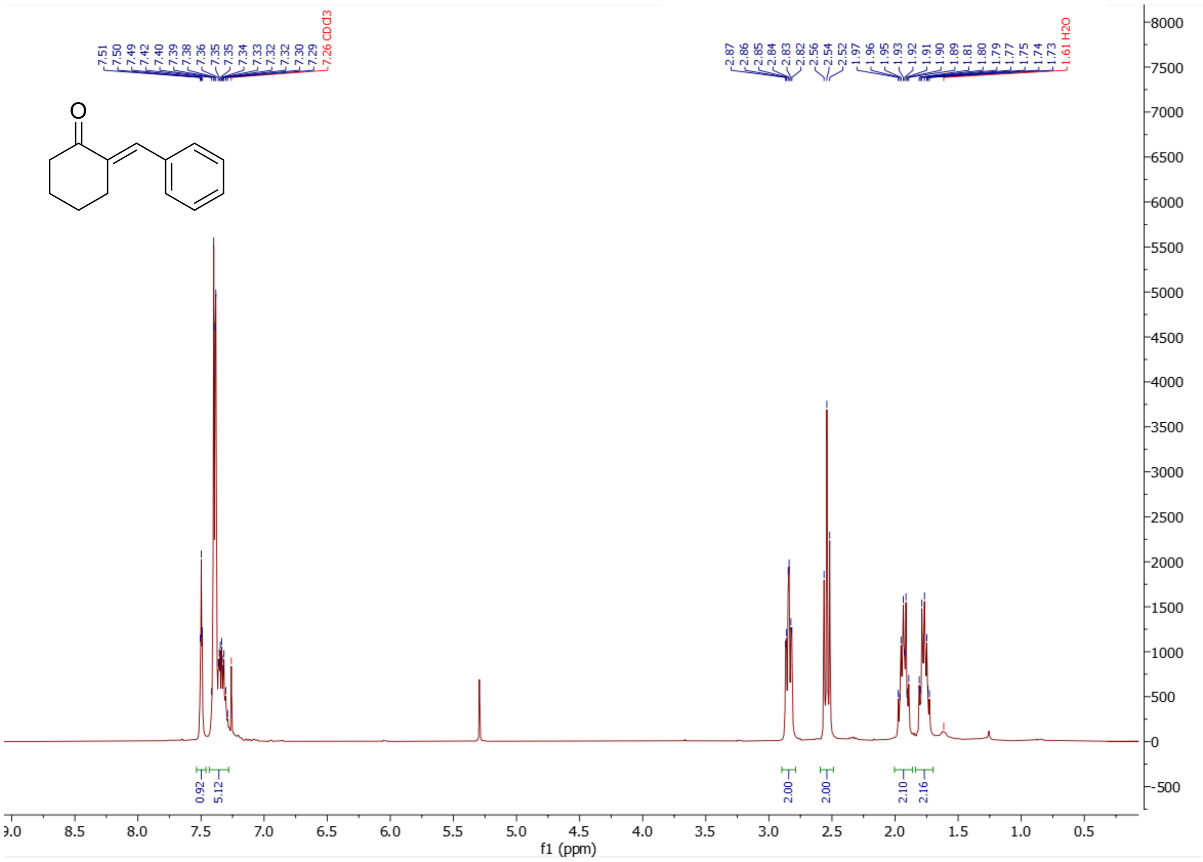

## (*E*)-2-(4-(2-chloropropanoyl)benzylidene)cyclohexan-1-one (B)

**$^{1}$H-NMR** (300 MHz; $CDCl_3$)

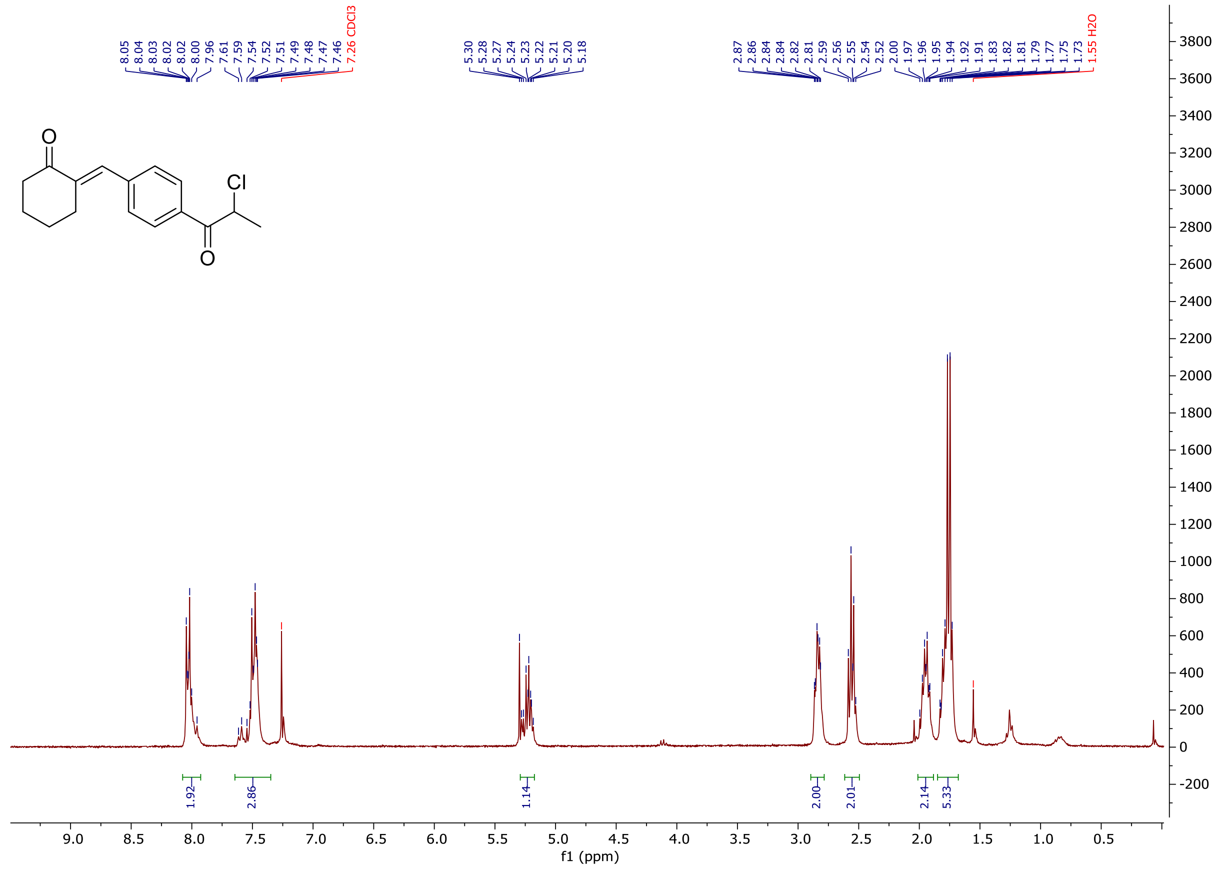


**$^{13}$C-NMR** (75 MHz; $CDCl_3$)

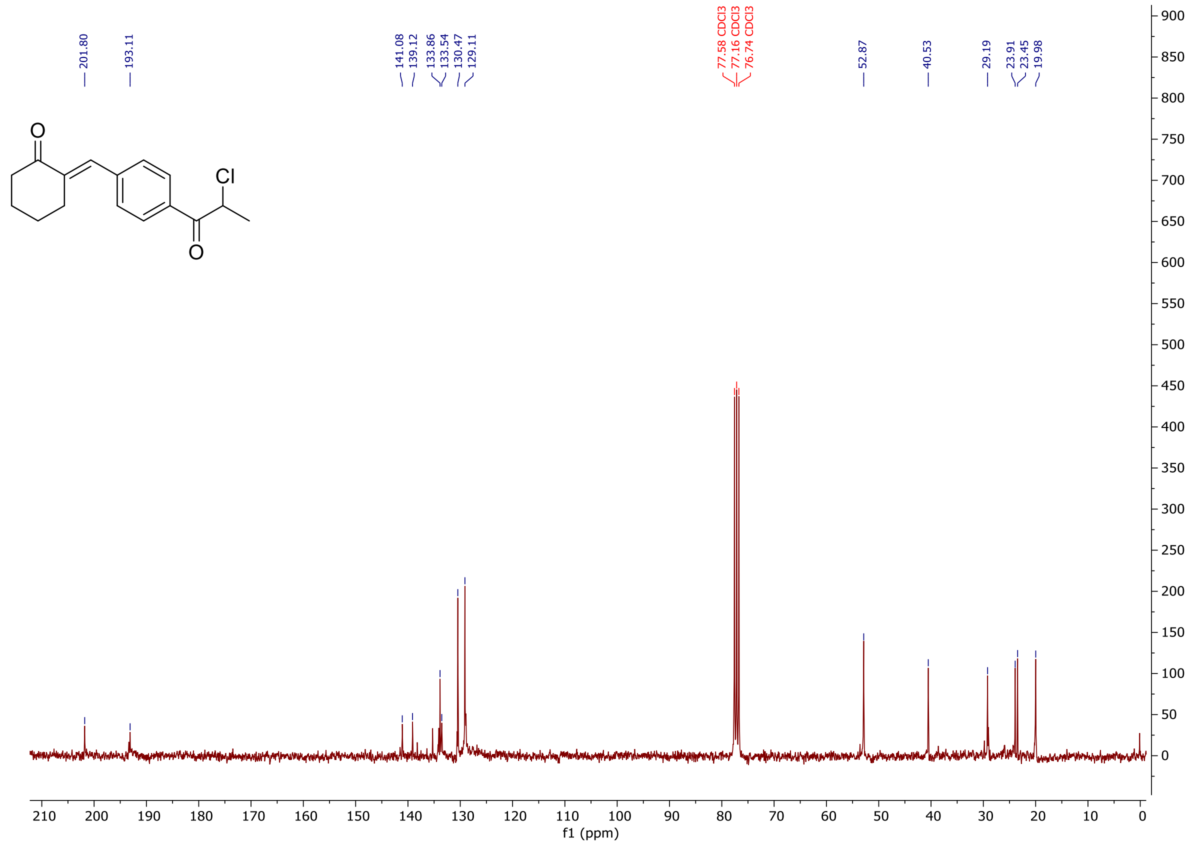

**COSY** ($CDCl_3$)

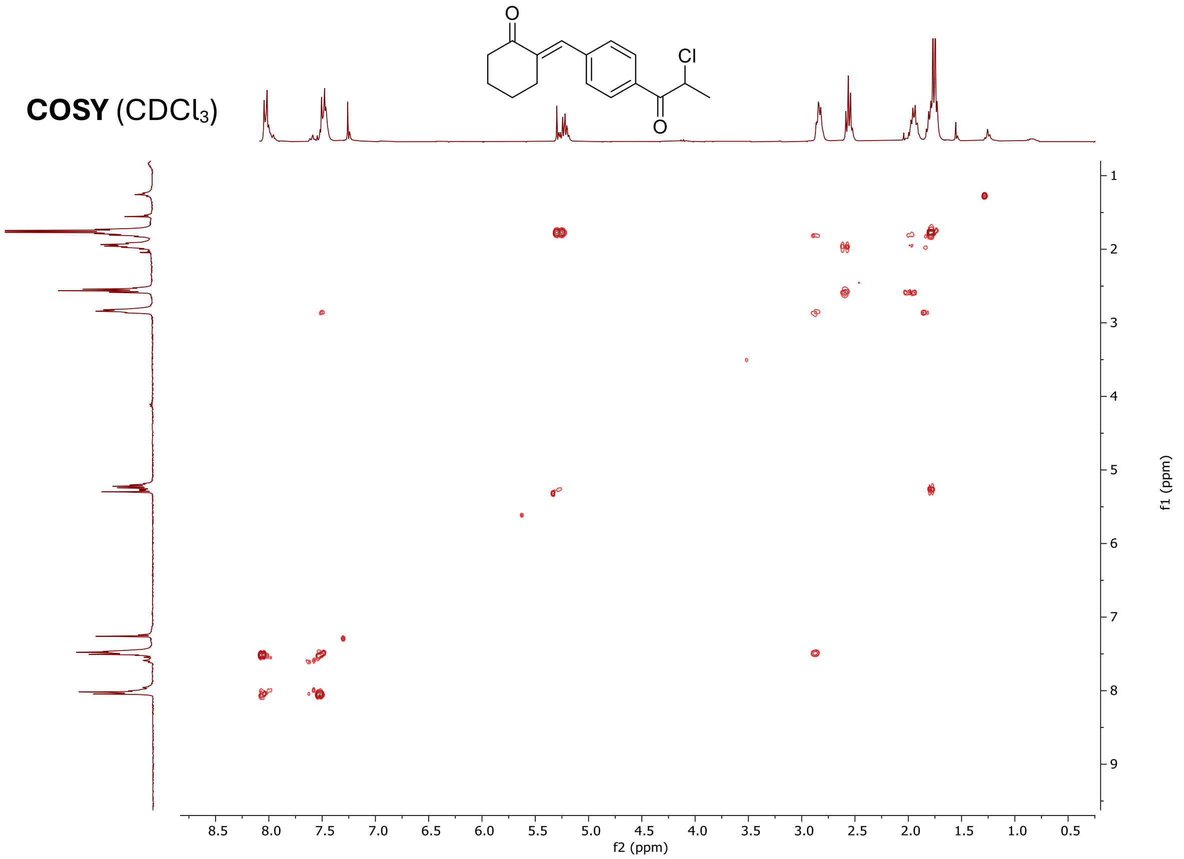


**HSQC** ($CDCl_3$)

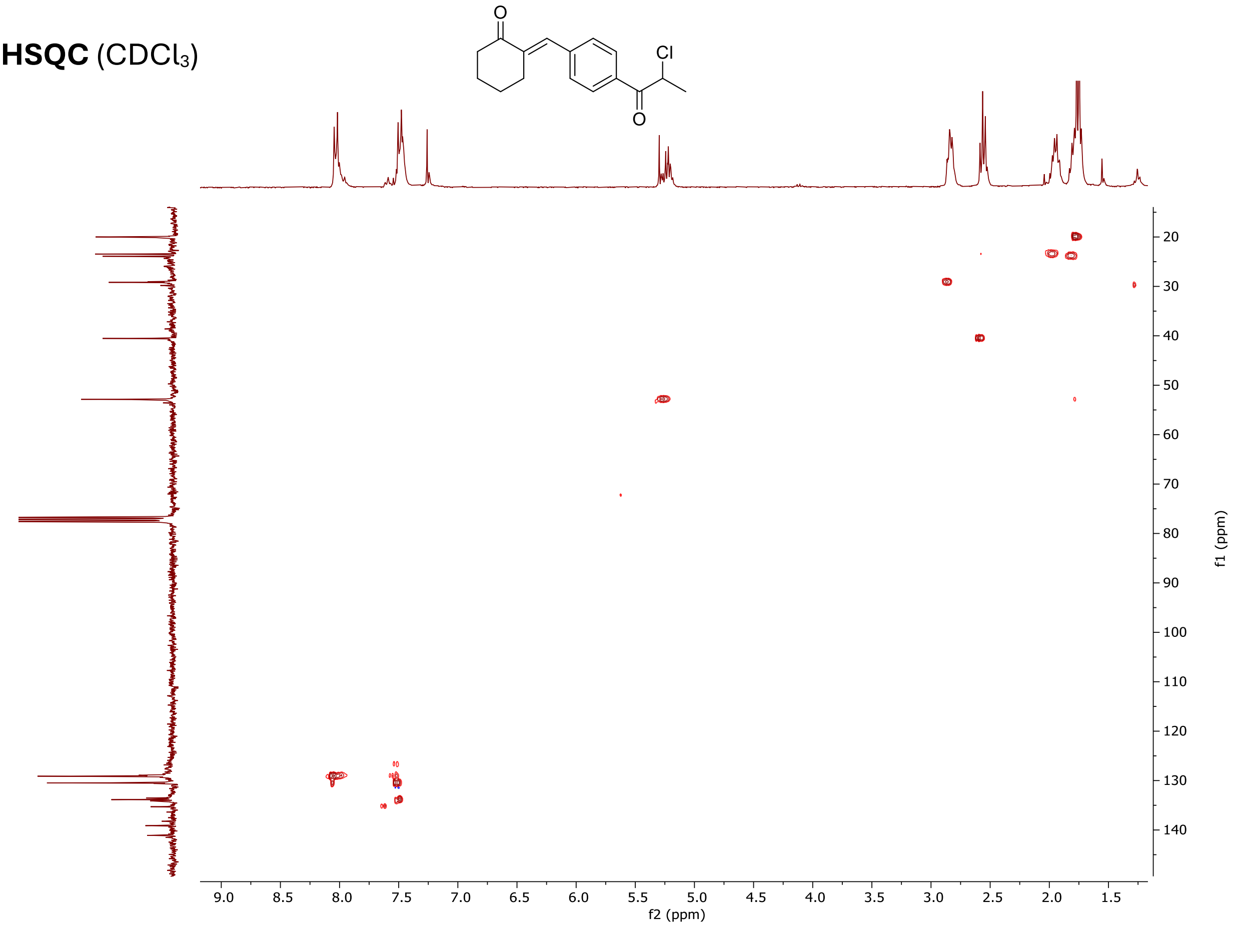

**HMBC** ($CDCl_3$)

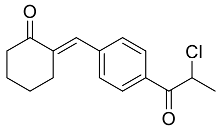


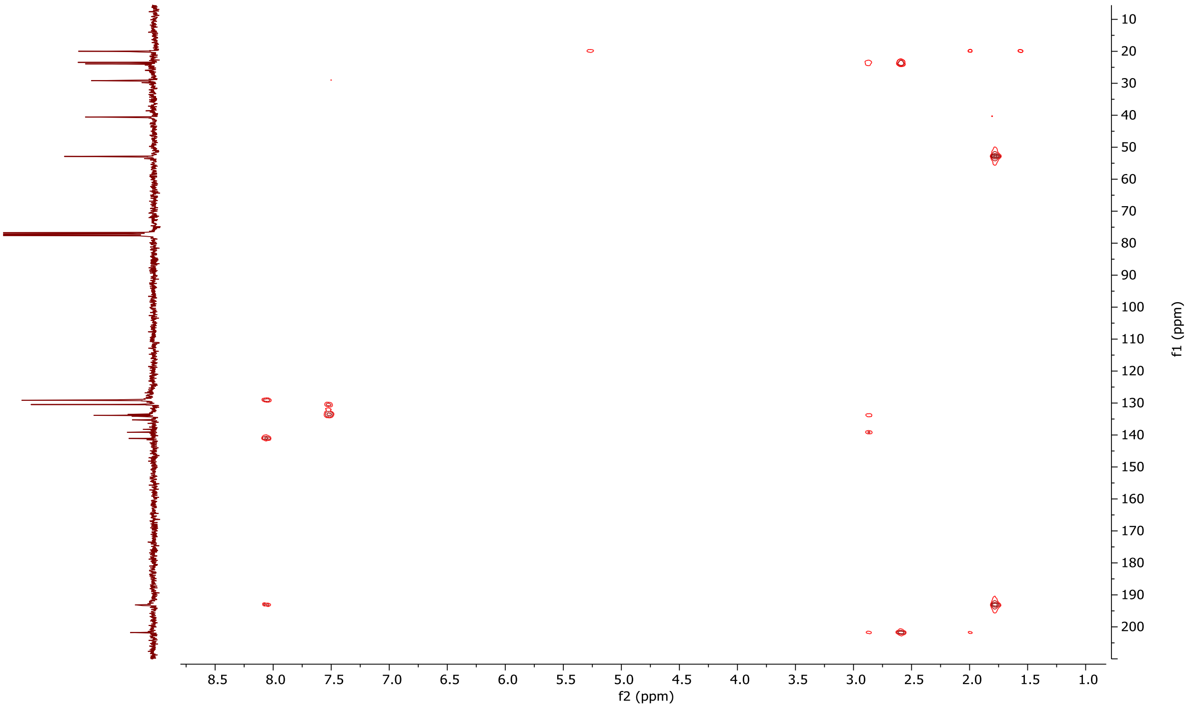


## (*E*)-1-oxo-1-(4-((2-oxocyclohexylidene)methyl)phenyl)propan-2-yl acetate (B’)

**$^{1}$H-NMR** (300 MHz; $CDCl_3$)

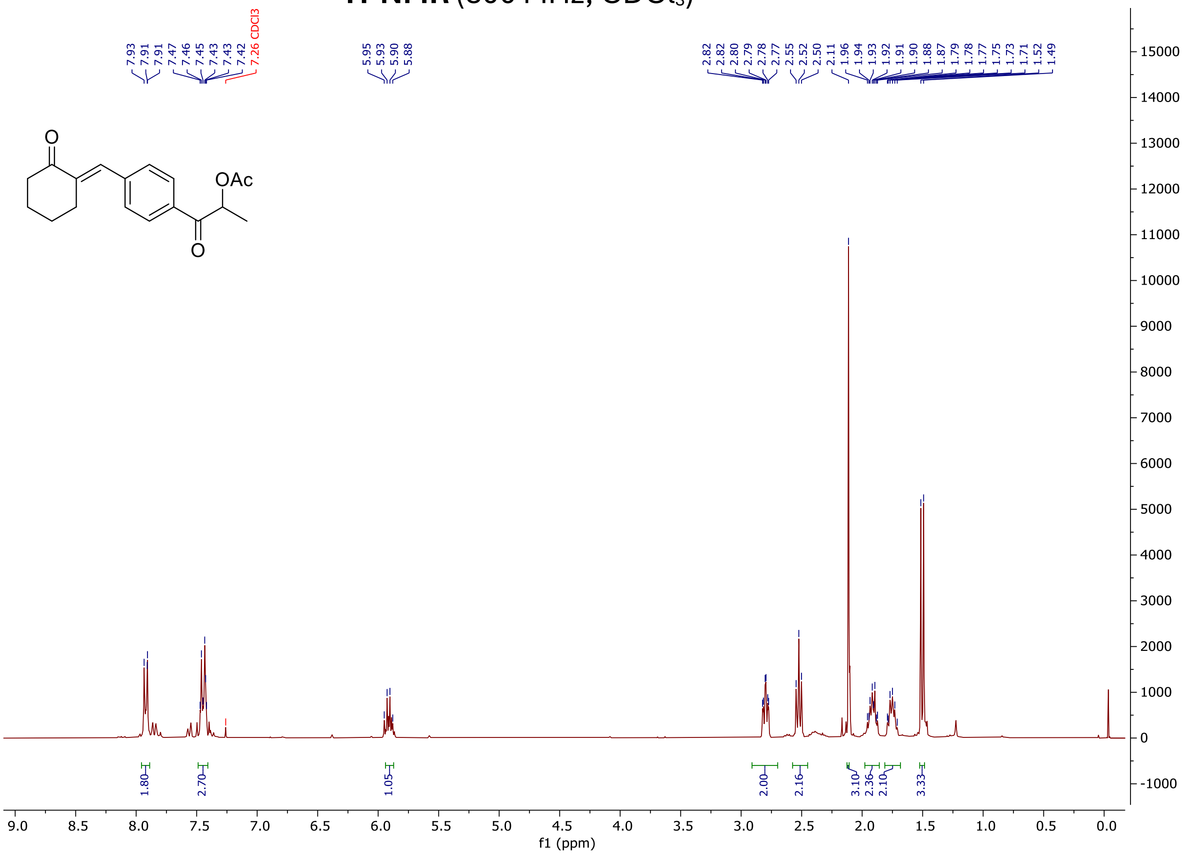

**$^{13}$C-NMR** (75 MHz; $CDCl_3$)

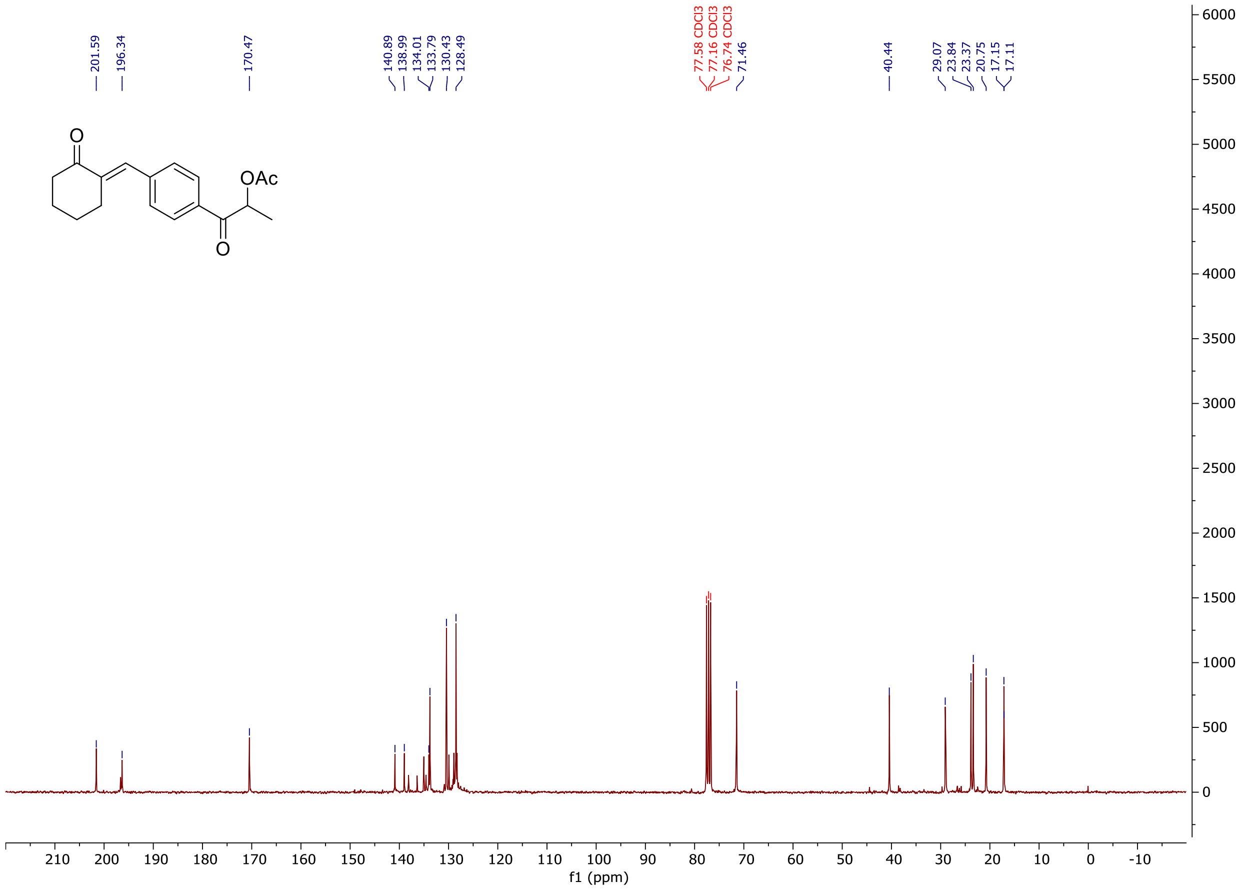


**COSY** ($CDCl_3$)

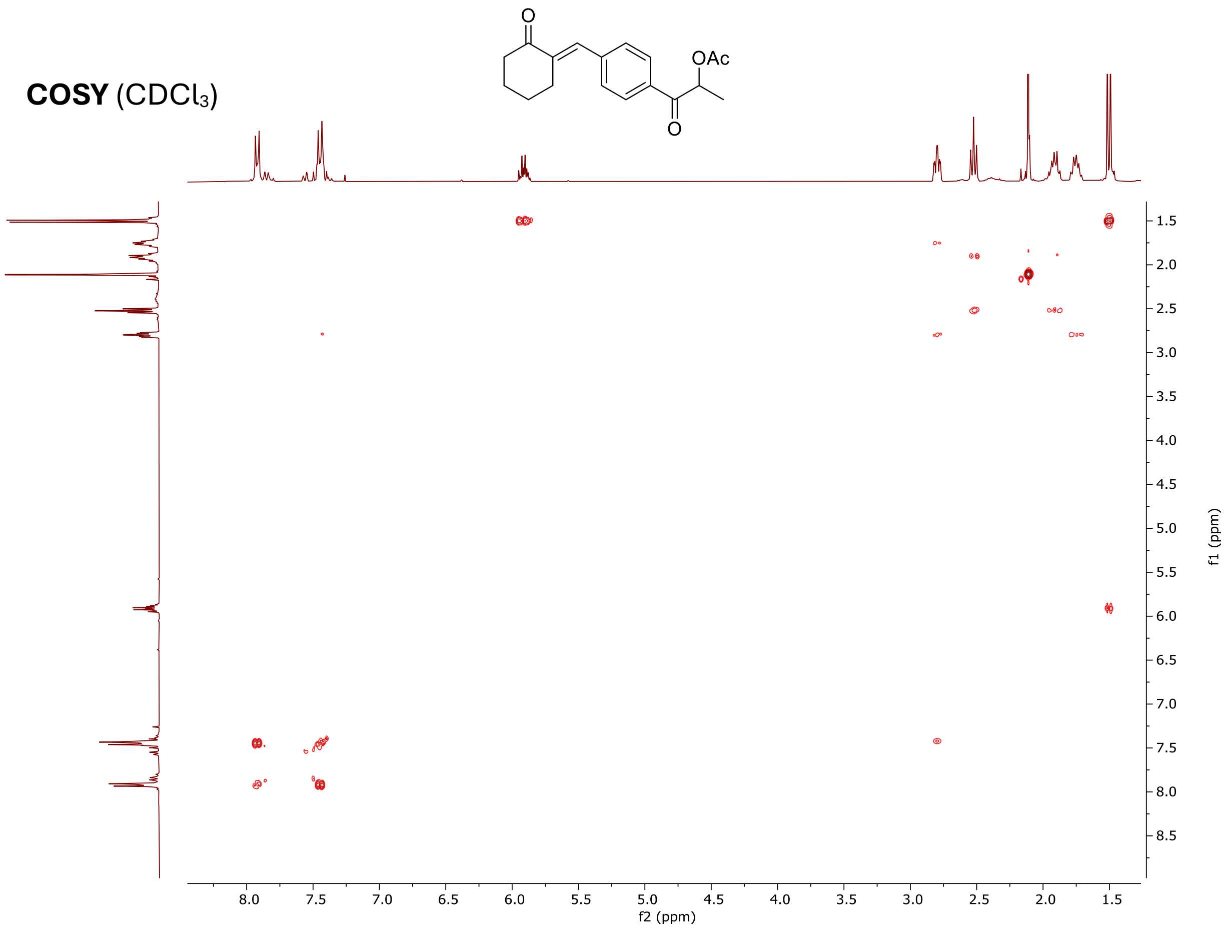

**HSQC** ($CDCl_3$)

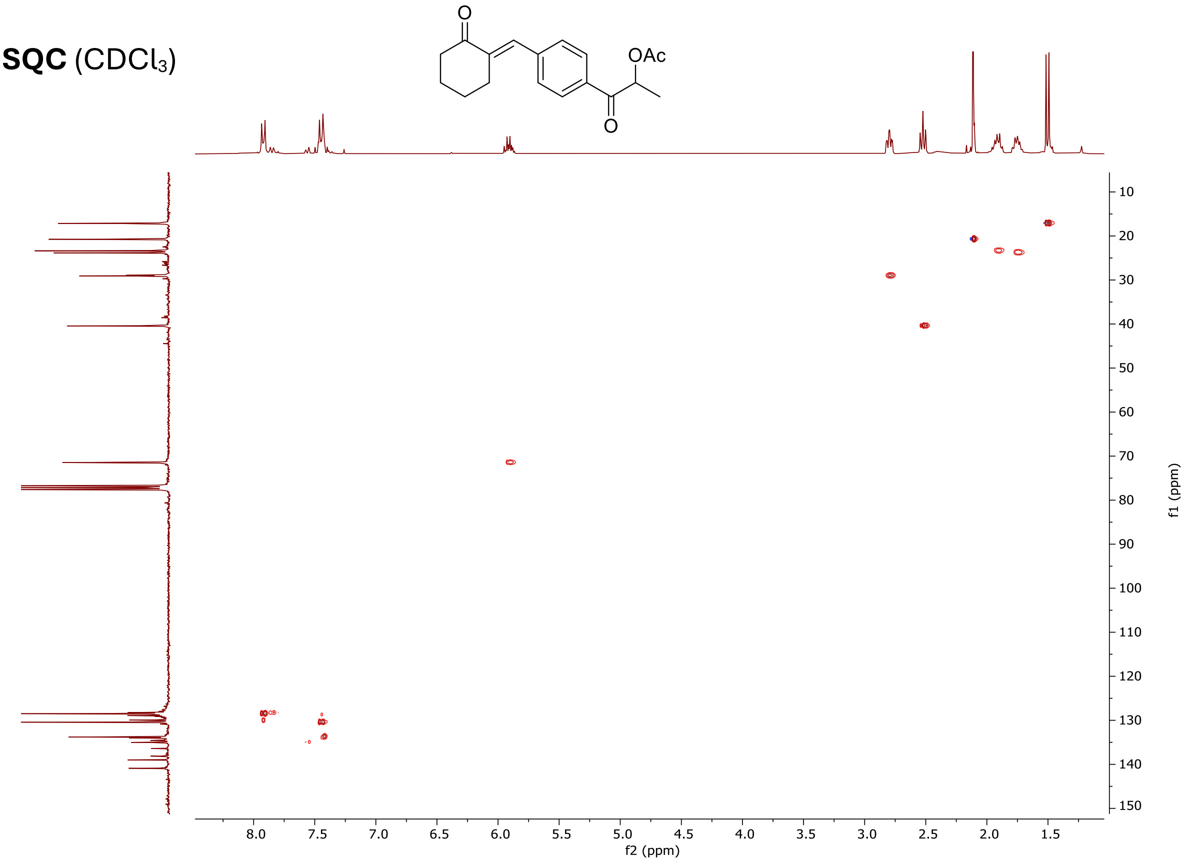


**HMBC** ($CDCl_3$)

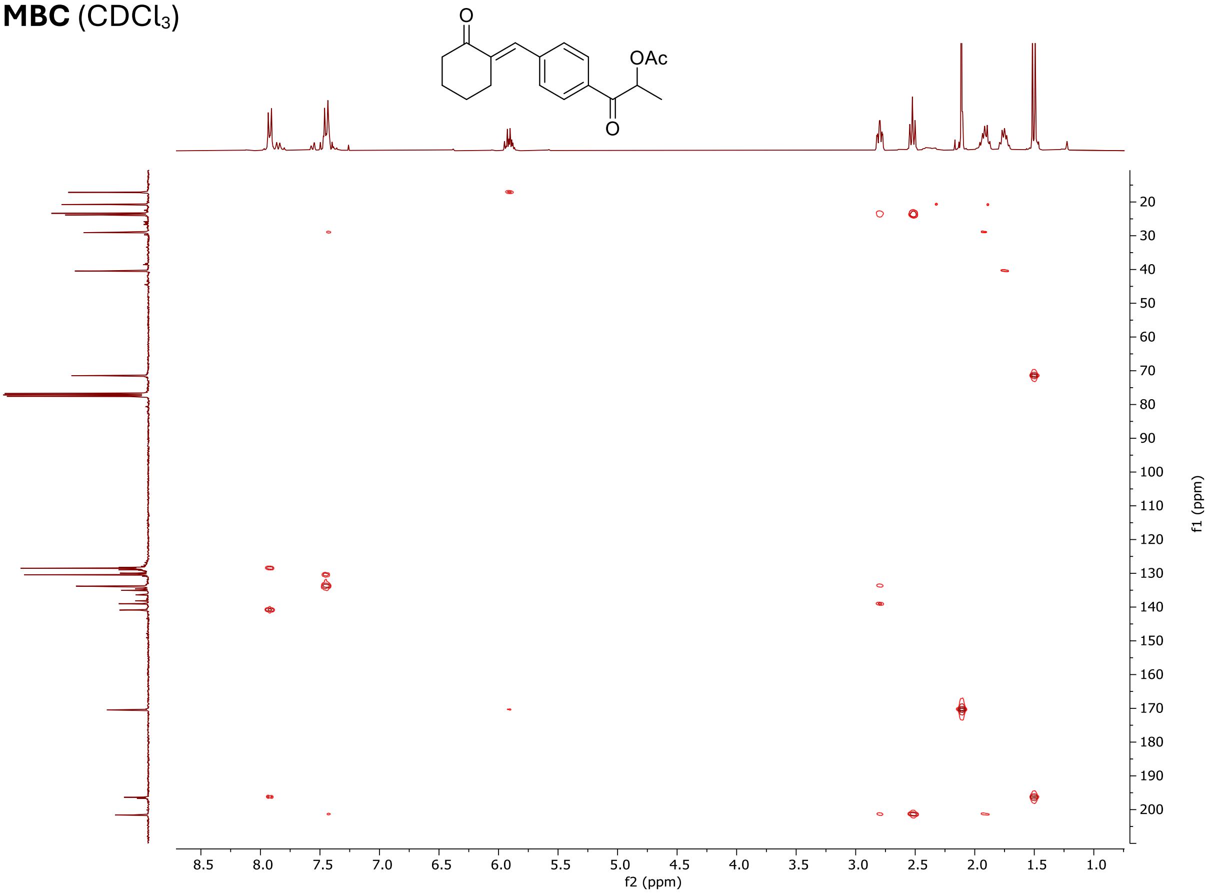

## 12. UV-Vis spectra

Carbazole intermediate **11** was kindly provided by Cosma S.p.A. and used without further purification.
The UV-Vis spectrum of **11** was recorded using a Nicolet Evolution 500 (a ThermoFisher spectrophotometer) and quartz cuvettes with path length of 1 mm. Acetonitrile was used as solvent, at a concentration of $4 \cdot 10^{-4}$ M; a bandwidth of 1 nm and a scan rate of 120 nm · $min^{-1}$ were used as analysis parameters.
The UV-Vis spectrum is reported in **Figure S5**.

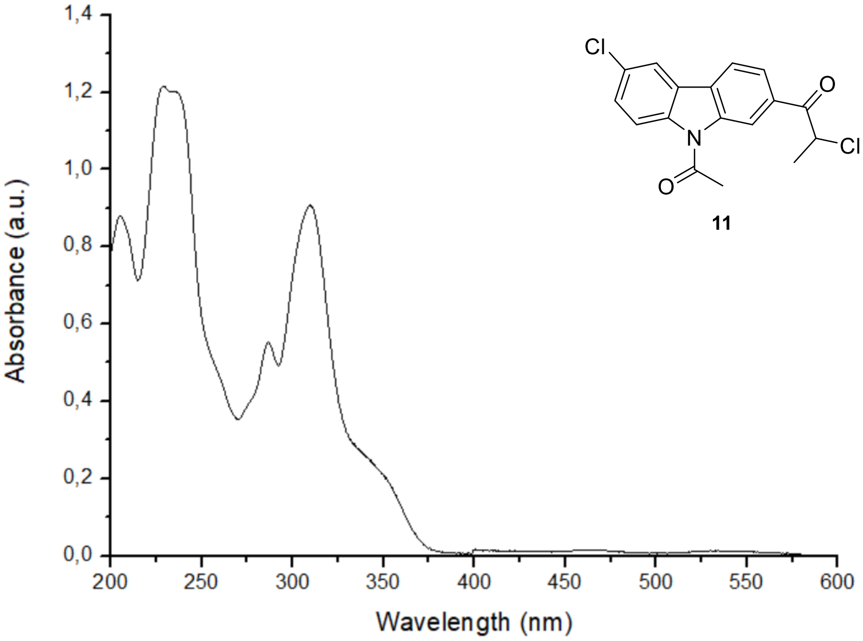


**11**

**Figure S5**: UV-Vis spectrum of compound **11**.

## 13. References

[1] Piccolo, O.; Spreafico, F.; Visentin, G.; Valoti, E. Zinc Salt Catalyzed Rearrangement of Acetals of Optically Active Aryl 1-Chloroethyl Ketones: Synthesis of Optically Active 2-Arylpropionic Acids and Esters. *J. Org. Chem.* **1987**, *52*, 10–14.

[2] Mayer, F. P.; Wimmer, L.; Dillon-Carter, O.; Partilla, J. S.; Burchard, N. V.; Mihovilovic, M. D.; Baumann, M. H.; Sitte, H. H. Phase I Metabolites of Mephedrone Display Biological Activity as Substrates at Monoamine Transporters. *British Journal of Pharmacology* **2016**, *173*, 2657–2668.

[3] Tassano, E.; Faber, K.; Hall, M. Biocatalytic Parallel Interconnected Dynamic Asymmetric Disproportionation of α-Substituted Aldehydes: Atom-Efficient Access to Enantiopure (*S*)-Profens and Profenols. *Adv Synth Catal* **2018**, *360*, 2742–2751.

[4] Gaster, E.; Kozuch, S.; Pappo, D. Selective Aerobic Oxidation of Methylarenes to Benzaldehydes Catalyzed by N-Hydroxyphthalimide and Cobalt(II) Acetate in Hexafluoropropan-2-ol. *Angew Chem Int Ed* **2017**, *56*, 5912–5915.

[5] Li, W-L.; Wu, Y.; Shi-Jun, L.; Yi-Qian, J.; Li, Y.-L.; Lan, Y.; Xia, J.-B. Boryl Radical Activation of Benzylic C–OH Bond: Cross-Electrophile Coupling of Free Alcohols and $CO_2$ via Photoredox Catalysis. *J. Am. Chem. Soc.* **2022**, *144*, 8551−8559.

[6] Liu, X.; Han, Z.; Wang, Z.; Ding, K. SpinPhox/Iridium(I)-Catalyzed Asymmetric Hydrogenation of Cyclic α-Alkylidene Carbonyl Compounds. *Angew Chem Int Ed* **2014**, *53*, 1978–1982.

[7] Wang, Y.; Yang, G.; Xie, F.; Zhang, W. A Ferrocene-Based NH-Free Phosphine-Oxazoline Ligand for Iridium-Catalyzed Asymmetric Hydrogenation of Ketones. *Org. Lett.* **2018**, *20*, 6135–6139.

[8] Peters, B. B. C.; Jongcharoenkamol, J.; Krajangsri, S.; Andersson, P. G. Highly Enantioselective Iridium-Catalyzed Hydrogenation of Conjugated Trisubstituted Enones. *Org. Lett.* **2021**, *23*, 242–246.

[9] Ruberg, L.; Small, L. Amino Alcohols Derived from Carbazole. *J. Am. Chem. Soc.* **1941**, *63*, 736–741.

[10] Berndsen, R.; Cunningham, T.; Kaelin, L.; Callender, M.; Boldog, W. D.; Viering, B.; King, A.; Labban, N.; Pollock, J. A.; Miller, H. B.; Blackledge, M. S. Identification and Evaluation of Brominated Carbazoles as a Novel Antibiotic Adjuvant Scaffold in MRSA. *ACS Med. Chem. Lett.* **2022**, *13*, 483−491.
